# Effect of Antimicrobial Consumption and Production Type on Antibacterial Resistance in the Bovine Respiratory and Digestive Tract

**DOI:** 10.1371/journal.pone.0146488

**Published:** 2016-01-28

**Authors:** Boudewijn Catry, Jeroen Dewulf, Dominiek Maes, Bart Pardon, Benedicte Callens, Mia Vanrobaeys, Geert Opsomer, Aart de Kruif, Freddy Haesebrouck

**Affiliations:** 1 Healthcare-Associated Infections and Antimicrobial Resistance, Scientific Institute of Public Health (WIV-ISP), Brussels, Belgium; 2 Faculty of Veterinary Medicine, Ghent University, Merelbeke, Belgium; 3 Animal Health Care Flanders (DGZ), Torhout, Belgium; Ross University School of Veterinary Medicine, SAINT KITTS AND NEVIS

## Abstract

The aim of this study was to investigate the relationship between antimicrobial use and the occurrence of antimicrobial resistance in the digestive and respiratory tract in three different production systems of food producing animals. A longitudinal study was set up in 25 Belgian bovine herds (10 dairy, 10 beef, and 5 veal herds) for a 2 year monitoring of antimicrobial susceptibilities in *E*. *coli* and *Pasteurellaceae* retrieved from the rectum and the nasal cavity, respectively. During the first year of observation, the antimicrobial use was prospectively recorded on 15 of these farms (5 of each production type) and transformed into the treatment incidences according to the (animal) defined daily dose (TI_ADD_) and (actually) used daily dose (TI_UDD_). Antimicrobial resistance rates of 4,174 *E*. *coli* (all herds) and 474 *Pasteurellaceae* (beef and veal herds only) isolates for 12 antimicrobial agents demonstrated large differences between intensively reared veal calves (abundant and inconstant) and more extensively reared dairy and beef cattle (sparse and relatively stable). Using linear mixed effect models, a strong relation was found between antimicrobial treatment incidences and resistance profiles of 1,639 *E*. *coli* strains (p<0.0001) and 309 *Pasteurellaceae* (p≤0.012). These results indicate that a high antimicrobial selection pressure, here found to be represented by low dosages of oral prophylactic and therapeutic group medication, converts not only the commensal microbiota from the digestive tract but also the opportunistic pathogenic bacteria in the respiratory tract into reservoirs of multi-resistance.

## Introduction

Associations between antimicrobial use and the prevalence of resistance in commensal faecal *E*. *coli* of cattle have been documented [1–3] but that detailed records on the antimicrobial regimens (e.g. dose) are limited. Such detailed records retrieved from swine production have allowed to show that many applied antimicrobial regimens deviate from leaflet instructions [4], which on their turn influence the occurrence of antimicrobial resistance in faecal *Enterobacteriaceae* [5]. No such information is currently available with regard to bovine livestock.

Large variations in antimicrobial resistance profiles of *Pasteurellaceae* between different bovine herds complicate empirical antimicrobial therapy of bovine respiratory disease [6,7]. Inclusion of these opportunistic pathogenic bacteria in studies exploring relationships with antimicrobial use might aid to explain different stakeholders of the clinical relevance of antimicrobial resistance.

The purpose of the present multi-centre study was to find associations between antimicrobial consumption data and the occurrence of antimicrobial resistance profiles in the bovine digestive (*E*. *coli*) and upper respiratory tract (*Pasteurellaceae*). The unit of measurements applied are derived from the Defined Daily Dose (DDD) methodology, which is recommended by the WHO to ensure comparison of selection pressure over time exerted between different niches [4].

## Materials and Methods

### Selection of the herds

An intensive monitoring programme on antimicrobial drug use was set up in 25 cattle herds in Belgium (Flanders) from April 2002 until January 2005. The participating herds were selected from the Belgian identification and registration system for livestock (SANITEL) and from the clients of the ambulatory clinic of the Faculty of Veterinary Medicine (Ghent University), and consisted of 10 dairy herds with a minimum of 40 lactating cows (Holstein Friesian), 10 beef herds (Belgian White Blue) with at least 30 newborn calves a year, and 5 veal calf farms. A key inclusion criterion was the willingness of the farmer to cooperate at the initiation of the survey. Breeds were Holstein Friesian and Belgian Blue in the dairy and the beef herds, respectively, except for two dairy herds where approx. 15% of the animals were Belgian Blue or mixed breeds. In most of the veal herds, several age groups (organised in pens) of predominantly male Holstein Friesian calves were present of which only one group was monitored. The size of the monitored groups varied between 144 and 594 animals. A further inclusion criterion consisted in the absence of other livestock animal species (e.g. swine, poultry) bred by the selected farms, so that interference of resistance selection due to antibiotic use for these animals was excluded. Herds taking part in the study were quality label certified.

### Study design

During the two year longitudinal survey, the 10 beef herds (B1-B10) were visited 4 times (every six months; time points I, II, III, IV) while the 10 dairy herds (D1-D10) were visited 6 times (every three months; time points I, II, III, IV, V, VI). The five veal herds (V1-V5) were monitored twice during one production cycle of approximately six months, at approximately 4 (T1) and 24 weeks (T2) after arrival at the farm (deviation in days ± 5). An overview of the study design with respect to sampling, number of animals, and their distribution over the different herd types is given in Table 1. Rectal samples consisted of taking approximately 5 g fresh faeces in a sterile recipient, while nasopharyngeal samples were obtained by means of cotton swabs (Venturi Transsystem^®^, Copan). All samples were stored at 5 +/- 3°C and bacteriological investigations were set up within 24 hours after arrival in the laboratory.

**Table 1:** Overview of the study design with respect to sampling scheme to monitor antimicrobial resistance in target bacteria.

Herd type	N herds	N sample periods (intensity)	N Animals	Target bacteria (ecosystem)
**Dairy herds**	10	6 (every 3 months)	50%^a^	*E*. *coli* (digestive tract)
**Beef herds**	10	4 (every 6 months)	50%^a^	*E*. *coli* (digestive tract)
				*P*. *multocida/M*. *haemolytica* (respiratory tract)
**Veal calves**	5	2 (start and end of production cycle)	20%	*E*. *coli* (digestive tract)
				*P*. *multocida/M*. *haemolytica* (respiratory tract)

^a^ On each sample occasion 50% of animals within following age categories were sampled: 0–0.5; 0.5–1; 1–2, >2 years. *E*: *Escherichia; P*: *Pasteurella; M*: *Mannheimia*

During the first year of monitoring in the 5 dairy and 5 beef herds, and during the full production cycle of the 5 veal calf herds, farmers were asked to continuously keep detailed antimicrobial consumption records, which consisted of the date of treatment, identification of the treated animal (ear tag), indication of treatment, body weight of the treated animal (weighed, estimated or determined by means of breed specific growth tables, product name, dose, administration route, and duration. The collected data were validated by checking treatment records supplied by the respective herd veterinarians. In case more than 10% deviations were noticed during this comparative control procedure, antimicrobial consumption records were considered insufficient en not retained for further analysis.

### Processing of antimicrobial consumption records

**Fig 1:**
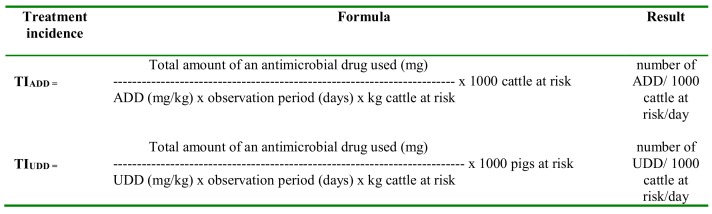
Formulae for the calculation of the treatment incidence (TI) based on the animal defined daily dose (ADD; TI_ADD_) and used daily dose (UDD; TI_UDD_).

Antimicrobial consumption records were converted into treatment incidences per farm (and per sample moment on the veal calves) based on the (animal) Defined Daily Dose (ADD) and used daily dose (UDD) by means of the formulae presented in Fig 1 [8]. ADD was defined as the average maintenance dose per day per kg animal of a drug for its main indication (equals animal defined daily dose or ADD [9]. DDD values were estimated based on the dosage recommendations of the Belgian compendia for veterinary drugs and are included in Tables 2–4. The data on drug use presented in this study were classified according to the ATCvet classification system [10]. The DDD were expressed per kg animal, except for the intramammary and intra-uterine formulations. For these preparations, the unit dose (UD) was used [11], where one UD equals the number of applications to be administered per 24 hours per quarter (expressed in mg) in the case of mastitis treatment, 4 applicators (expressed in mg) in the case of dry cow therapy, and the recommended 24 hour dose in the case of intra-uterine treatments. For other long-acting preparations, the recommended dosage was transferred into a 24 hours dose. The range of recommended doses was limited, except for ampicillin, amoxicillin, trimethoprim-sulphonamide combinations, and tetracycline for intra-uterine use (Tables 2–4). In contrast to the ADD which is based on the leaflet of a certain product for its main indication (national registration), the UDD reflects the actually administered dose per day per kg of animal based on the (individual) animal records.

**Table 2:** Treatment incidences (min-max) based on the animal unit dose (UD; TI_UD_) and prescribed daily dose (UDD; TI_UDD_) of the topical antimicrobial drugs in the different herd types.

Active substance	ATCvet^a^	Indication	UD (mg)	Dairy herds		Beef herds	
Intramammary drugs				TI_UD_	TI_UDD_	TI_UD_	TI_UDD_
Procaine penicillin+ neomycin	QJ51RC	M^d^	500	0.07	0.03	-	-
				(0.00–0.43)	(0.00–0.22)		
Cloxacillin	QJ51CF	M	100	0.23	0.06	-	-
				(0.00–1.14)	(0.00–0.31)		
Cloxacillin	QJ51CF	D^e^	2200	0.55	0.55	-	-
				(0.00–1.11)	(0.00–1.11)		
Nafcillin+benzylpenicillin+DHS^b^	NA^c^	M	100	0.59	0.45	0.06	0.06^g^
				(0.00–1.42)	(0.00–1.21)	(0.00–0.49)	(0.00–0.49)
Nafcillin+benzylpenicillin+DHS	NA	D	400	0.24	0.24	-	-
				(0.00–1.22)	(0.00–1.22)		
Cefquinome	QJ51DA	M	150	0.43	0.60	-	-
				(0.00–1.02)	(0.00–0.97)		
Cefalexin	QJ51DA	M	400	0.35	0.39	-	-
				(0.00–2.05)	(0.00–2.31)		
Lincomycin+neomycin	QJ51RF	M	660	0.17	0.29	0.03	0.03^g^
				(0.00–0.43)	(0.00–0.71)	(0.00–0.30)	(0.00–0.30)
Pirlimycin	QJ51FF	M	50	0.21	0.10	-	-
				(0.00–1.03)	(0.00–0.51)		
**Total**				**2.83**	**2.71**	**0.09**	**0.09**
				(1.81–3.70)	(0.94–3.83)	(0.00–0.79)	(0.00–0.79)
**Intra-uterine drugs**							
Cefapirin	QG01DA	V^f^	500	0.17	0.16	<0.01	<0.01
				(0.00–0.78)	(0.00–0.77)	(0.0–0.02)	(0.0–0.02)
Chlortetracycline hydrochloride	QG01AA	V	1500	0.25	0.16	0.78	1.07
				(0.00–0.52)	(0.00–0.34)	(0.36–1.08)	(0.39–1.62)
**Total**				**0.42**	**0.32**	**0.78**	**1.07**
				(0.00–1.27)	(0.00–1.11)	(0.38–1.08)	(0.39–1.62)
**Grand total topical use**				**3.25**	**3.03**	**0.87**	**1.16**

^a^Anatomical Therapeutic Chemical classification system for Veterinary medicinal products

^b^dihydrostreptomycin

^c^not available

^d^M: mastitis

^e^D: dry cow therapy

^f^V: variable (prophylactic and/or therapeutic)

^g^ TI_UD_ values were used to calculate the total TI_UDD_

**Table 3:** Average treatment incidences (min-max) based on animal defined daily dose (ADD; TI_ADD_) and used dose (UDD; TI_PDD_) of the parenteral antimicrobial drugs in the three different herd types.

Active substance	ATCvet^a^	ADD	Dairy herds		Beef herds		Veal herds	
		(mg/kg)	TI_ADD_	TI_UDD_	TI_ADD_	TI_UDD_	TI_ADD_	TI_UDD_
Penethamaat hydroiodide	QJ01CE	12.5	0.04	0.03	-	-	-	-
			(0.00–0.23)	(0.00–0.18)				
Procaine benzylpenicillin	QJ01CE	12	0.58	0.15	2.12	0.87	-	-
			(0.00–1.63)	(0.00–0.27)	(0.00–4.39)	(0.00–1.70)		
Procaine benzathine benzylpenicillin LA^b^	QJ01CE	3	0.32	0.17	0.54	0.29	-	-
			(0.00–0.72)	(0.00–0.38)	(0.00–1.35)	(0.00–0.79)		
Procaine benzylpenicillin+DHS^c^	QJ01RC	8	0.52	0.25	0.04	0.02	0.04	0.01
			(0.00–3.24)	(0.00–1.58)	(0.00–0.42)	(0.00–0.12)	(0.00–0.30)	(0.00–0.10)
Procaine benzylpenicillin+neomycin	QJ01RC	10	0.25	0.21	0.84	0.74	0.05	0.03
			(0.00–0.99)	(0.00–0.96)	(0.03–1.94)	(0.03–1.82)	(0.00–0.28)	(0.00–0.17)
Ampicillin	QJ01CA	28	<0.01	<0.01	<0.01	<0.01	-	-
			(<0.01)	(<0.01)	(<0.01)	(<0.01)		
Ampicillin LA	QJ01CA	11.25	<0.01	<0.01	<0.01	<0.01	-	-
			(<0.01)	(0.00–0.08)	(<0.01)	(<0.01)		
Amoxicillin	QJ01CA	9	<0.01	<0.01	<0.01	<0.01	0.09	0.06
			(<0.01)	(<0.01)	(0.00–0.01)	(<0.01)	(0.00–0.22)	(0.00–0.14)
Amoxicillin LA	QJ01CA	6.25	-	-	-	-	0.32	0.28
							(0.00–0.71)	(0.00–0.72)
Amoxicillin+clavulanic acid	QJ01CA	7	-	-	0.05	0.04	-	-
					(0.00–0.15)	(0.00–0.12)		
Ceftiofur	QJ01DA	1	0.30	0.15	0.01	0.01	2.32	1.27
			(0.00–0.81)	(0.00–0.34)	(0.00–0.05)	(0.00–0.05)	(0.00–5.79)	(0.00–3.07)
Cefquinome	QJ01DA	1	0.06	0.04	-	-	0.60	0.60
			(0.00–0.19)	(0.00–0.13)			(0.00–3.14)	(0.00–3.19)
Tylosin	QJ01FA	7.5	-	-	-	-	0.23	0.15
							(0.00–1.30)	(0.00–0.83)
Tilmicosin	QJ01FA	10	-	-	0.03	0.03	0.64	0.64
					(0.00–0.14)	(0.00–0.11)	(0.00–2.14)	(0.00–2.07)
Lincomycin	QJ01FF	10	-	-	0.02	0.06	-	-
					(0.00–0.05)	(0.00–0.19)		
Lincomycin+spectinomycin	QJ01FF	5	0.01	<0.01	0.03	0.02	0.86	0.87
			(0.00–0.02)	(0.00–0.04)	(0.00–0.27)	(0.00–0.18)	(0.00–2.41)	(0.00–2.76)
Gentamicin	QJ01GB	3.75	0.03	0.04	-	-	0.02	0.03
			(0.00–0.14)	(0.00–0.17)			(0.00–0.11)	(0.00–0.16)
TMP^d^+sulphonamides	QJ01EW	4.25	0.46	0.84	0.04	0.05	-	-
			(0.00–1.74)	(0.00–3.46)	(0.00–0.11)	(0.00–0.18)		
TMP+sulphonamides LA	QJ01EW	1.2	-	-	-	-	0.29	0.29
							(0.00–1.63)	(0.00–1.66)
Oxytetracycline	QJ01AA	3	0.13	0.08	<0.01	<0.01	-	-
			(0.00–0.24)	(0.00–0.22)	(0.00–0.02)	(0.00–0.16)		
Enrofloxacin	QJ01MA	2.5	0.25	0.21	<0.01	<0.01	0.78	0.52
			(0.00–1.54)	(0.00–1.45)	(0.00–0.02)	(0.00–0.06)	(0.00–2.16)	(0.00–2.08)
Danofloxacin	QJ01MA	1.25	0.01	<0.01	0.05	0.05	0.18	0.09
			(0.00–0.03)	(0.00–0.02)	(0.00–0.19)	(0.00–0.19)	(0.00–0.50)	(0.00–0.33)
Marbofloxacin	QJ01MA	2	-	-	-	-	0.09	0.05
							(0.00–0.25)	(0.00–0.28)
Florfenicol	QJ01BA	10	0.03	<0.01	-	-	0.22	0.30
			(0.00–0.08)	(0.00–0.02)			(0.00–1.26)	(0.00–1.68)
Colistin	QJ01XB	50	-	-	-	-	0.01	0.02
							(0.00–0.04)	(0.00–0.04)
**Total (average)**			**3.01**	**2.36**	**3.78**	**2.15**	**6.74**	**5.21**

^a^Anatomical Therapeutic Chemical classification system for Veterinary medicinal products long acting

^b^long acting

^c^dihydrostreptomycin

^d^trimethoprim. Recommended dosages, and thereby DDDs (defined daily doses), according to more recent leaflets and registrations might differ (eg. cephalosporines and fluoroquinolones).

**Table 4:** Average treatment incidences (min-max) based on the animal defined daily dose (ADD; TI_ADD_) and the prescribed daily dose (UDD; TI_UDD_) of the orally administered antimicrobial drugs in the veal herds (entire study period).

Active substance	ATCvet^a^	ADD (mg/kg)	Veal herds	
			TI_DDD_	TI_UDD_
Amoxicillin	QJ01CA	15	10.92	53.35
			(0.00–43.67)	(0.00–106.71)
Tylosin	QJ01FA	16	12.34	271.64
			(0.00–44.88)	(0.00–393.80)
Trimethoprim+sulphonamide	QJ01EW	30	17.09	17.01
			(0.00–30.23)	(0.00–28.34)
Oxytetracycline	QJ01AA	30	62.40	62.89
			(10.08–178.05)	(12.85–143.63)
Doxycycline	QJ01AA	10	21.06	52.86
			(0.00–40.94)	(0.00–76.70)
Colistin	QJ01XB	5	13.75	27.56
			(0.00–32.75)	(0.00–40.94)
Flumequine	QJ01MA	12	6.82	40.94
			(0.00–20.47)	(0.00–40.94)
**Total**			**135.25**	**324.12**

^a^Anatomical Therapeutic Chemical classification system for Veterinary medicinal products

For the dairy and the beef herds, the population at risk was defined as all cattle present in the herd during the observation period due to the (globally) constant animal population throughout the study period in these herds. In the veal herds, only the number of animals in the monitored age group was considered as being at risk. The number of animals present in the herds was available from the Belgian identification and registration system (SANITEL) at the beginning and at the end of the observation period. For the dairy and beef herds, the observation period was the first year of monitoring. To calculate the kg of animals at risk in the dairy and the beef herds, the animals were divided in 4 age categories (<6 months, 6–12 months, 13–24 months and >24 months) and the average number of animals in each age category was multiplied by the average weight of an animal of that specific age category. To determine the kg of animals at risk [9] in the veal herds, the number of animals was multiplied by the average live body weight at the moment of treatment [4,8]. Because the majority of treatments in veal calves were administered as group medication, these treatments were used to determine the average live body weight at the moment of treatment. In the veal herds, a medicated starter ration was defined as the treatment with antimicrobial drugs from the day of arrival on the farm. The relative importance of each administered antimicrobial was expressed by the proportional TI_ADD (UD)_ and the proportional TI_UDD_. These were calculated by dividing the TI_ADD_ or the TI_UDD_ of each antimicrobial by the total TI_ADD_ or the total TI_UDD_, respectively.

### Bacteriology

For the isolation of *E*. *coli*, approximately 1 g faeces was diluted into 9 mL sterile PBS (phosphate buffered saline) and an aliquot was plated onto MacConkey agar (MAC; Oxoid, Basingstoke, UK) for overnight aerobic incubation at 37°C. Per sample, one colony of *E*. *coli* was purified and identified as previously described (Catry et al., 2007a). Out of the nasal swabs, *Pasteurella* (*P*.) *multocida* and *Mannheimia* (*M*.) *haemolytica* (sensu lato) were isolated on a selective medium (Columbia agar (Oxoid) to which 5% sheep blood and 16 μg/mL bacitracine were added) and identified as previously described [6,7]. Strains were stored at -70°C prior to susceptibility testing.

### Antimicrobial susceptibilities

After thawing at room temperature, bacteria were grown up overnight at 37°C on Columbia sheep blood agar (Oxoid) under aerobic conditions and checked for purity. The antimicrobial resistance profile was determined for each isolate by means of the Kirby-Bauer disk diffusion method for following antimicrobial agents (abbreviation, content in μg): ampicillin (AMP, 33), amoxicillin + clavulanic acid (AMC, 30+15), ceftiofur (CEF, 30), oxytetracycline (TET, 80), trimethoprim-sulphonamides (TMPS, 5.2 + 240), neomycin (NEO, 120), gentamicin (GEN, 40), spectinomycin (SPT, 200), nalidixic acid (NAL, 130), flumequine (FLU, 30) and enrofloxacin (ENR, 10). In addition, for *Pasteurellaceae* florfenicol (FLO, 30) was tested and for *E*. *coli* streptomycin (STR, 100). CLSI guidelines (NCCLS-2002 M31-A2) were followed during the test procedure for standardisation of the inoculum (0.5 McFarland), incubation conditions, and internal quality control organism (*Escherichia coli* ATCC 25922). Media used were Mueller Hinton II (Oxoid) for *E*. *coli*, and Mueller Hinton II with 5% sheep blood (Oxoid) for *Pasteurellaceae* [6,7]. Reading of inhibition zones (in mm) was performed by the semi-automated SIRscan 2000 device (i2a, Montpellier, France), and interpretation was done according to the manufacturer of the disks (Guidelines for the use of Neosensitabs 18^th^ Ed., 2005/2006, www.rosco.dk). An isolate was considered ‘resistant’ if resistance or intermediate resistance was observed for at least one antimicrobial agent tested.

### Statistical analysis

For all bacterial species isolated, the resistance level was quantified by means of antimicrobial resistance index (ARI) which is calculated as the number of antimicrobials against which resistance is detected divided by the total number of antimicrobials tested [12]. Also for these analyses intermediate results were considered resistant. The ARI can vary from 0.00 (0%), when the strain is (fully) susceptible to every tested antimicrobial agent, to 1.00 (100%) when the strain is (pan-) resistant to all tested antimicrobial agent classes. It enables to estimate selection pressure exerted on one bacterium for different antimicrobial agents and can be used for different bacterial species [6,13].

The relationship between the antimicrobial treatment incidences and the ARI was analysed using a linear mixed effect model with herd included as random effect, and herd type and age (in months) included as fixed effects. Hereby total treatment incidences (TI_ADD_ and TI_UDD_) for the individual farms were considered, consisting of the sum of the different treatment incidences for oral, parenteral, and topical use.

Analysis were performed with STATA version 10.0 (Stata Corporation, College Station, TX, USA). The significance level was set P< or = 0.05.

The experimental protocol (sampling methodology) was approved by the local ethics committee (Ethical Committee of the Faculty of Veterinary Medicine).

## Results

### Antimicrobial consumption data

Assessment of the quality of obtained antimicrobial consumption records resulted in a restriction of the analysed period to 1 year in the participating herds, and 1 production cycle in the veal calf farms. Detailed data on antimicrobial use by route of administration for the first year of the study period in the 5 dairy and the 5 beef farms, and the average of the total production cycle (6 months) in the 5 veal calf farms are presented in Tables 2 to 4. On 1 veal farm, the individual parenteral treatments before 16 weeks of age were not recorded.

On the dairy herds, both topical (intramammary and intra-uterine) and parenteral formulations were most commonly administered (Tables 2 & 3). The total TI_UD_ for the intramammary antimicrobial drugs was 2.8, which means that on a total of 1000 cows, 2.8 cows were treated daily with a standard dose of an intramammary antimicrobial drug during the observation period. Overall, the most frequently used intramammary antimicrobial drugs were beta-lactamase resistant penicillins in combination with other antimicrobial drugs, beta-lactamase resistant penicillins and cephalosporins. Their proportional TI_UD_ were 29.3%, 27.6% and 27.4%, respectively. The total TI_UD_ for the intra-uterine antimicrobial drugs was 0.4 (Table 2). The main indications to use intra-uterine cephalosporins and tetracyclines were retained fetal membranes (*retentio secundinarum)* and endometritis.

For the parenterally administered antimicrobials, the total TI_ADD_ was 3.0. A comparison with the TI_UDD_ shows that in reality fewer animals were treated: 2.4 per 1000 on average in the dairy herds (Table 3). For the most frequently injectable antimicrobials, the proportional TI_ADD_ were: narrow-spectrum penicillins (31.2%), narrow-spectrum penicillins in combination with other antimicrobials (25.7%), trimethoprim-sulphonamide combinations (15.1%) and cephalosporins (12.1%). Proportional TI_ADD_ were different from proportional TI_UDD_. Mastitis was the main indication for the use of narrow-spectrum penicillins, and locomotory problems were the main indication for the use of narrow-spectrum penicillins in combination with other antimicrobial drugs.

The total TI_ADD_ on the beef herds was 5.4, whereas the total TI_UDD_ was 4.9. Mainly parenteral antimicrobial drugs were used in the beef herds. Narrow-spectrum penicillins and narrow-spectrum penicillins in combination with other antimicrobial drugs (Table 3) were the most often applied parenteral antimicrobial drugs. Their proportional TI_ADD_ were 70.4% and 23.3%, respectively. The main indication to use these antimicrobial drugs was surgical prophylaxis during caesarean section [14]. Fluoroquinolones were not frequently used (1.3%). The total TI_UD_ for the topical treatments was 0.9, compared to a slightly more frequent use in reality (TI_PDD_ = 1.2). Tetracyclines administered intrauterinely were the most frequently applied topical antimicrobial drugs (88.7%). Surgical prophylaxis during caesarean section and retained fetal membranes were the two main indications. Intramammary antimicrobial drugs were not frequently used in the beef herds (Table 2). The oral treatments consisted of colistin (polymyxin E) (92.2%) and trimethoprim-sulphonamide combinations (7.8%) for the prevention and treatment of diarrhea (metaphylaxis) in calves.

In the veal herds, oral group treatments accounted for the largest amount of antimicrobial drug use. Over the 6 month production cycle, the total TI_DDD_ for the orally administered antimicrobial drugs was 135.25, though in reality many more calves were treated with antimicrobials since the TI_UDD_ was 324.12 (Table 4). Tetracyclines (54.2%) and tylosin (24.2%) were used most often. The main indication was prophylactic or metaphylactic treatment of respiratory problems. On one farm (V1), tylosin was also used to prevent *Clostridium perfringens* enterotoxaemia. The duration of the oral treatments generally varied between 5 and 13 days, but in two occasions, tylosin was administered for 41 and 73 days, respectively. A significant amount of the oral antimicrobial drugs was given as a medicated starter ration (23.0%). Proportionally, colistin (55.2%), oxytetracycline (29.5%) and trimethoprim-sulphonamide combinations (10.1%) were most frequently used for this. Medicated starter rations were applied in all veal herds and their duration varied between 6 and 13 days. For the injectable antimicrobials, the TI_ADD_ was much lower, i.e. 6.7 (Table 3). The proportional TI_ADD_ for the most frequently used injectable antimicrobial drugs were: cephalosporins (43.4%), fluoroquinolones (15.6%), macrolides (12.9%) and lincosamides (12.8%). In contrast with the dairy and beef farms where only small variations were seen for treatment incidences over time, the treatment incidences on the veal calf herds at the beginning and the end of the production cycle differed substantially.

### Prevalence of bacteria and susceptibility profiles

The 10 dairy and 5 veal calf farms all completed the study (6 and 2 sampling moments, respectively). Out of the 10 beef farms, only 9 remained in the study during the second and third sampling moment, whereas only 7 herds completed the full study period (4 sampling moments) (Table 5). Drop out of the study was due to loss of interest/ lack of time (n = 1) and termination of livestock production (n = 2). In addition, the number of animals within several beef herds dropped during the study period. Susceptibility profiles were determined for 4174 *E*. *coli* isolates out of a total of 4552 rectal samples (91.7%) from the different sampling moments (range 84.2–98.7%) on the 10 dairy (2373/2549), 10 beef (1295/1468), and 5 veal calf herds (506/535).

**Table 5:** Overview of resistance percentages of *E*. *coli* during the consecutive sampling periods on the different production types.

Production type	sample moment(N herds)	N. Isolates	ARI^a^	AMP^b^	AMC	CEF	TET	TMP	NEO	GEN	SPT	STR	NAL	FLU	ENR
**Dairy**	I (10)	447	0.04	2.91	0.45	0.45	8.28	4.25	0.67	1.12	0.22	24.83	1.34	0.22	0
	II (10)	396	0.01	2.02	0.25	0	3.79	0.25	1.52	0	0.25	4.55	0.76	0.25	0.25
	III (10)	419	0.02	4.3	0.24	0	4.3	3.58	2.15	0.48	0	7.88	1.19	0.72	0.24
	IV (10)	359	0.01	2.79	0.28	0.28	3.06	0.84	0.84	0	0	4.74	0.28	0.28	0.28
	V (10)	382	0.02	5.24	1.05	0.26	3.66	3.56	0.52	1.05	0.79	8.64	0.79	0.26	0.26
	VI (10)	370	0.02	3.24	0.54	0	5.14	1.89	1.62	1.08	0.54	6.76	1.35	0.54	0.27
**Beef**	I (10)	436	0.03	9.17	1.15	0	6.88	4.13	2.52	0.92	0.69	13.3	2.52	0.46	0.46
	II (9)	346	0.06	12.14	1.45	0.58	17.05	5.49	4.91	2.31	0.87	18.21	8.67	4.33	2.89
	III (9)	298	0.05	9.4	1.34	0.34	12.08	6.71	5.03	1.01	0.34	13.76	5.03	4.36	2.35
	IV (7)	215	0.09	17.21	1.4	0	15.81	6.05	7.45	2.33	1.4	24.19	8.37	5.12	4.19
**Veal**	T1 (5)	276	0.62	93.12	4.71	0.36	94.93	92.75	83.33	45.29	22.46	89.49	79.00	73.13	64.23
	T2 (5)	230	0.32	79.57	2.61	1.74	95.22	65.22	27.83	5.22	5.65	78.26	14.01	6.22	4.12
**Overall**	(25)	4,174	0.09	17.08	1.24	0.26	19.03	13.75	9.67	4.52	2.37	22.02	8.74	6.70	5.58

^a^ ARI: average antimicrobial resistance index (ARI) per sample moment.

^b^ AMP, ampicillin; AMC, amoxicillin + clavulanic acid; CEF, ceftiofur; TET, oxytetracycline; TMP, trimethoprim-sulphonamides; NEO, neomycin; GEN, gentamicin; SPT, spectinomycin; STR, streptomycin; NAL, nalidixic acid; FLU, flumequine; ENR, enrofloxacin.

During the two year study period, antimicrobial resistance among *E*. *coli* was in general relatively stable in the dairy and beef herds (Table 5), with minimum-maximum values for the average ARI per sample period for the dairy and beef herds of 0.00–0.096, and 0.00–0.196, respectively. A slightly higher number of *E*. *coli* was resistant (or intermediate resistant) for at least one antimicrobial agent in the beef herds (20.62%) compared to the dairy herds (12.05%). The highest variability was seen for STR and TET in both herd types. The percentage of multiple resistance (resistance for more than 3 agents) was 22.3% and 6.4% among the beef and dairy isolates, respectively. The majority of multi-resistant strains (67.3%) were retrieved from animals less than 6 months old. The resistance patterns most frequently encountered among the isolates from the dairy herds were TET/STR (16.5%), TET (13.5%), and STR (11.5%). Among the isolates from the beef herds, AMP/TET/STR (15.9%), TET/STR (11.4%), and AMP/STR (9.8%) were the most prevalent.

**Fig 2:**
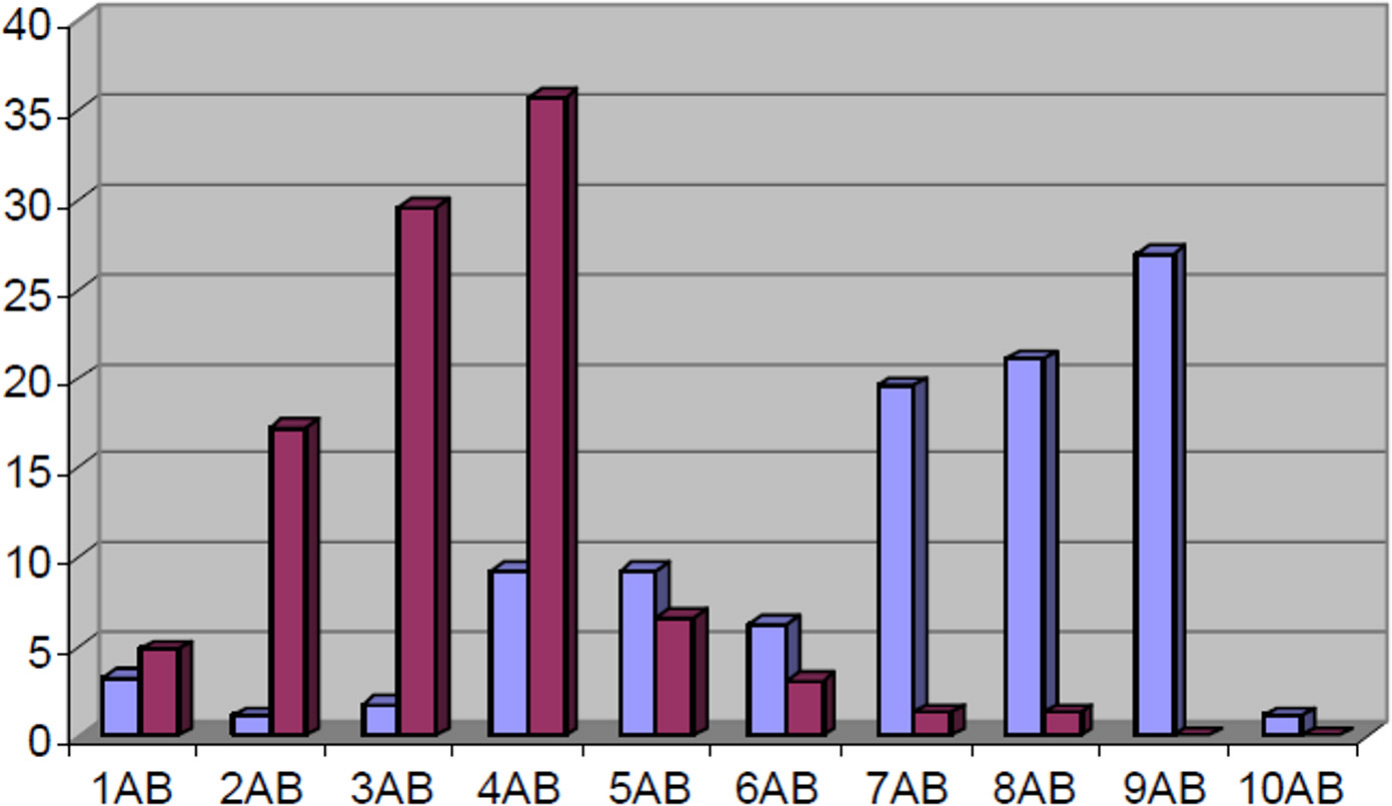
Dynamical distribution of multi-resistance in veal calves. Proportion of strains (*Y*-axis, %) resistant for indicated number of antibiotics (*X*-axis: 1AB; resistance to 1 agent tested, 2AB; resistance to 2 agents tested, etc.) of faecal *E*. *coli* (N = 506) retrieved during the production cycles at T1 (blue bars; 4 weeks +- 5 days after arrival, N = 276) and T2 (red bars; 24 weeks +- 5 days after arrival, N = 230) from 5 veal calves farms.

In the veal calves herds, substantially higher values compared to the dairy and beef herds were found and the minimum-maximum values for the average ARI per sample period were 0.284–0.759. Here, large differences were noticed between the first (T1) and second (T2) sample moment of the 6-month production cycle, especially for AMP, TMPS, NEO, GEN, SPT, STR, NAL, FLU, and ENR (Table 5). Although the overall percentage of faecal *E*. *coli* isolates resistant for at least one compound was highly comparable between T1 (97.83%) and T2 (98.82%), the number of multiple resistant isolates clearly decreased by the end of the production cycle: whereas 68.9% of all strains at T1 were resistant for 7 or more antimicrobial agents, only 12.4% of isolates at T2 were resistant for 5 or more antimicrobial agents. The most prevalent (24.8%) multi-resistance profile at T1 was AMP/TET/STR/NEO/GEN/TMPS/NAL/FLU/ENR. At T2, the most prevalent (27.9%) resistant profile was AMP/TET/STR/TMPS. The dynamical pattern of multiple resistances among *E*. *coli* in the veal calf herds is shown in Fig 2.

A total of 1217 nasal swabs were taken from 10 beef (682) and 5 veal calf (535) herds, resulting in 268 (39.3%) and 206 (38.5%) *Pasteurellaceae* strains from the beef and veal calf herds, respectively, that underwent susceptibility testing. Two hundred and six *P*. *multocida* isolates and 42 *M*. *haemolytica* (*sensu lato*) isolates originated from the beef herds, whereas 163 *P*. *multocida* and 43 *M*. *haemolytica* isolates originated from the veal calves. An overview of the resistance profiles, including the average ARI value per sample period, found for the *Pasteurellaceae* according to the production type of origin is given in Table 6. In the beef farms, the predominant resistance found was SPT (n = 44) which was found only on two farms. In one of the latter herds, it was present during the 4 consecutive sampling periods in both *P*. *multocida* (4 periods, n = 41) and *M*. *haemolytica* (2 periods, n = 2). In the veal calf farms, 83.1% of all resistant (or intermediate resistant) *P*. *multocida* isolates were resistant for at least 4 antimicrobial agents at T1. At T2, multiple resistances had decreased with 58.3% of all strains showing resistance to maximum 2 compounds tested. The most predominant resistance profile of *P*. *multocida* at T1 was GEN/TMS/FLU/NAL (58%). At T2, the latter pattern had decreased to the second most resistance profile (19%) after TET (58%). Among the resistant *M*. *haemolytica* isolates from the veal calves, 80% was resistant for 4 or more antimicrobial agents at T1. Similarly to the situation for *P*. *multocida*, multiple resistance decreased at T2, with 74% of the resistant *M*. *haemolytica* strains showing resistance to 1 or 2 compounds. At T1, resistance towards AMP/TET/GEN/TMPS/NAL/FLU/ENR was most common (30%), whereas at T2 the most frequent combinations of resistance were AMP/TET (35%) and AMP (32%).

**Table 6:** Overview of resistance percentages of *Pasteurellaceae* during the consecutive sampling periods on the different production types.

Production type	sampling moment (N herds)	N. Isolates	ARIndex^a^	AMP^b^	AMC	CEF	TET	TMP	NEO	GEN	SPT	FLO	NAL	FLU	ENR
**Beef**	I (10)	94	0.03	0.00	0.00	0.00	0.00	17.78	1.11	1.11	15.56	0.00	1.11	1.11	0.00
	II (9)	78	0.01	1.49	0.00	0.00	0.00	2.99	0.00	0.00	10.45	0.00	0.00	0.00	0.00
	III (9)	42	0.03	0.00	0.00	0.00	0.00	2.63	0.00	0.00	28.95	0.00	0.00	0.00	0.00
	IV (7)	54	0.02	0.00	0.00	0.00	0.00	0.00	2.04	2.04	24.49	0.00	0.00	0.00	0.00
**Veal**	T1 (5)	93	0.27	8.60	0.00	0.00	38.71	50.54	45.16	43.01	4.30	0.00	48.39	44.09	36.56
	T2 (5)	113	0.15	29.82	0.00	0.00	42.98	29.82	18.42	14.91	1.75	0.00	19.30	16.67	10.53
**Overall**	(15)	474	0.11	9.53	0.00	0.00	18.85	22.17	14.41	13.08	11.09	0.00	15.08	13.53	10.20

^a^ ARI: average antimicrobial resistance index (ARI) per sample moment.

^b^ AMP, ampicillin; AMC, amoxicillin + clavulanic acid; CEF, ceftiofur; TET, oxytetracycline; TMP, trimethoprim-sulphonamides; NEO, neomycin; GEN, gentamicin; SPT, spectinomycin; FLO, florfenicol; NAL, nalidixic acid; FLU, flumequine; ENR, enrofloxacin.

### Relationship between antimicrobial use and resistance

Association between the treatment incidences of antimicrobial agents (TI_ADD_ and TI_UDD_) and antimicrobial resistance (ARI) was tested for a total of 1639 *E*. *coli* isolates from 5 dairy (1year; 3 sampling periods, N = 635), 5 beef (1 year; 2 sampling periods, N = 497), and 5 veal calf herds (6 months; T1; N = 276, T2, N = 231). Over the three production types, a significant relationship was found by multivariate analysis between the ARI for *E*. *coli* with the TI_ADD_ (P<0.001) and TI_UDD_ (P<0.001), with herd type showing a significant effect (P<0.001) but age did not (P>0.35).

The association between treatment incidences and ARI was investigated for 309 *Pasteurellaceae* originating from 5 beef (2 sampling periods, N = 102) and 5 veal calf farms (T1; N = 93, T2; N = 114). At the beef and veal calf herds, the ARI of the *Pasteurellaceae* showed a significant effect with the TI_ADD_ (P = 0.012) and TI_UDD_ (P = 0.002). In these analyses, also the herd type was demonstrated to exert significant effect (P = 0.012).

Thus, analyses of both faecal *E*. *coli* and respiratory *Pasteurellaceae* demonstrated a significant difference in occurrence of antimicrobial resistance directly related to the production type. In addition to this effect, the consumption of antimicrobials also largely influenced the occurrence of antimicrobial resistance in both the digestive (*E*. *coli*) and the respiratory tract (*Pasteurellaceae*).

## Discussion

In the present study, the antimicrobial use was monitored in bovine herds using a multi-centre approach and an intensive sample strategy of the digestive and respiratory tract. The approach was original since it allowed prospectively investigating associations between antimicrobial drug use and antimicrobial resistance simultaneously in two ecosystems. Due to the intensive sampling and registration protocol that required well motivated farmers it was not possible to select the herds at random. Another limitation of the study design was information bias in the herds due to incomplete or inaccurate consumption records. Also, the here applied treatment incidences only give indirect information on the duration (length in days) of therapy [4], while this contributing factor is not taken into account during the analysis. For the susceptibility testing the disk contents (μg active antimicrobial agent in tablets) deviated from CLSI standards and the diffusion test for *Pasteurellaceae* is known to have clear limitations for certain antimicrobial compounds [7]. The here applied primary variable of interest was the antimicrobial resistance index (ARI) [12]. It is a straightforward way to describe multidrug resistance across different bacterial species, and enables to model a historical selection pressure within a certain ecological niche [6]. The ARI is function of related resistance mechanisms over different compounds tested (e.g. the quinolones; cross-resistance) and can be strongly influenced by linked resistance genes (e.g. plasmids; co-resistance). To exclude a major influence of related resistance determinants inherent to our selection of antimicrobial agents, the analysis between TI and antimicrobial resistance among faecal *E*. *coli* was repeated ([13]–S1 File). Therefore we redefined the ARI upon only 7 antimicrobial compounds (AMP, TET, ENR, GEN, SPT, NEO, TMP) instead of 12, and the analysis confirmed a similar significant association with the TI_PDD_ (P<0.001) and with the TI_DDD_ (P<0.001), suggesting the bias due to the choice of antimicrobial agents was not substantial.

In relation to the reliability of our antimicrobial consumption records, no large discrepancies were seen in our antimicrobial consumption data with regard to main indications and antimicrobial classes compared to previous reports on 110 randomly selected dairy herds [15], on 105 selected beef herds [16] and on 6 [17] and 15 [8] veal calf farms. Our results also demonstrated substantially large differences in antimicrobial consumption patterns between the herd types. This was illustrated by means of both the ADD and the UDD methodology. From the TI_UDD_ calculations, it was shown that the largest deviations from the recommended regimens according to the leaflets were mainly restricted to the veal calves. About 2.5 times more veal calves were treated with antimicrobial agents than the number of calves for which that amount -present on the farm- was intended (Table 4), resulting in 88.0% of oral administrations that were underdosed. A similarly high degree of underdosing was also found in 50 randomly selected Belgian swine herds and was also seen for oral group treatments [4]. On the other hand, in the porcine study injectable antimicrobial agents were mostly overdosed, which is in line with the here presented bovine data. As these factors are of relevance for the development of specific guidelines for a judicious use of antimicrobial agents [18], the underlying reasons for choosing between oral and injectable antimicrobial regimens have been discussed before in detail [4].

For long *Escherichia coli* has been used in order to measure antimicrobial resistance in a variety of ecological niches, including human and animal settings. In adult dairy cattle, resistance among *E*. *coli* was sparse and in agreement with an Australian study by Jordan and colleagues [19]. The age related decline in overall resistance of enteric *E*. *coli*, irrespective of antimicrobial use [2, 20–21], was also reflected here on the extensively reared herds. Detailed studies have suggested that this might be the consequence of an effect of milk diet (in particular a vitamin D component) to select for multi-resistant *E*. *coli* in calves [22]. The here found remarkable dynamical shift in resistance profiles over the production cycle in the veal calves might reflect this effect of the milk diet, combined with what has been postulated based on similar observations in swine [21], namely that following oral administration of antimicrobial agents a relatively large number of resistant strains occurs in the faeces rather than the dominance of a limited number of resistant clones. Before arrival in the veal herds, calves might have been fed waste milk from intramammary treated dairy cows at the herd of origin. This practice might also have exerted a selection pressure. At the time of the investigations, veal (white) calves were usually raised solely on a milk diet throughout the entire production cycle, whereas other calves are weaned at the first half of the same age period.

The sampling strategy for faecal *E*. *coli* was very intense compared to other surveillance or monitoring programmes, in which resistance percentages of cattle isolates were always found to be in favour compared to swine and poultry [23–25]. Our results are in line with a distinction in resistance abundance at slaughter between veal calves [26] and less intensively reared beef or dairy cattle [3,23], which is relevant for public health surveillance. Compared to our observations, Sawant et al. found a very high frequency of resistant *E*. *coli*’s among adult dairy cattle [27]. This is likely the consequence of the use of selective media containing antimicrobials procedure known to substantially increase the sensitivity of rating antimicrobial resistance among *Enterobacteriaceae*.

Of the many studies dealing with resistance of *E*. *coli* in cattle, the number of studies including detailed antimicrobial consumption records is sparse. In agreement with our study, Berge et al. experimentally found that in-feed antimicrobials were associated with higher levels of multiple antimicrobial resistance whereas individual antimicrobial therapy was associated with increased but transient resistance [1]. Similarly, Di Labio et al. [28] also found a protective effect of antimicrobial injection upon arrival versus in milk medication (oral) for antimicrobial resistance in Swiss veal calves. Recent research has also highlighted the difference of route of administration with regard to number of both resistant bacteria and genes in the intestinal tract [29–30]. The marked differences in ARI between intensively reared veal calves and the other production type also results likely from the different predominant route of administration across these settings. The selection pressure exerted by a certain therapy on a certain microbiome, indeed, is influenced by pharmacokinetics like absorption rate, protein binding, metabolisation, elimination half-life, tissue distribution, and enterohepatic cycling.

In human medicine, observations in *E*. *coli* have revealed that treatment incidences (TI_DDD_), for one particular compound or related agents, are not always associated with resistance for that compound, while in general total antimicrobial consumption (TI_DDD_) can correlate well with the occurrence of antimicrobial resistance [31]. In agreement with the latter study, we found the ARI derived from *E*. *coli* to be significantly associated with the two consumption parameters TI_ADD_ and TI_UDD_. A relationship between the consumption of a certain antimicrobial compound and the occurrence of resistance in the digestive tract (or co-selected traits by linked resistance genes), has been documented for particular molecules in different animal species for *E*. *coli* and for other commensal bacteria like enterococci [1,32].

A significant association was found between antimicrobial consumption and the occurrence of resistance in the respiratory tract, through screening of *Pasteurellaceae*, among the most important bovine—and in a larger context veterinary—respiratory pathogens. Malhotra-Kumar et al. have shown in a randomised controlled clinical trial that a selection pressure exerted by oral antimicrobial therapy also is reflected in the resistance situation of commensal oral streptococci, and the authors refer to these bacteria as a source of resistance genes for respiratory pathogens [33]. Our observational study design comparing exposed versus non exposed animals was able to demonstrate that opportunistic respiratory pathogenic bacteria themselves can act as a reservoir of resistance. In view of the similarities of respiratory disease over the different animal species, with *Pasteurellaceae* (e.g. *Haemophilus influenzae*) or other secondary invaders being involved frequently, it seems plausible to believe that identical processes are taking place when a certain critical antimicrobial consumption level is attained.

A high population density combined with cross-infection and co-selection are suspected to increase the risk for the spread and persistence of antimicrobial resistance, as seen in human medicine for intensive care units [34]. In livestock, similar conditions have been found in intensive production systems like industrialised poultry [32] and swine [5], and our results indicate this is also applicable to densely housed veal calves. Antimicrobial resistance selection plus specific persistence over consecutive production cycles [32] can explain the intense accumulation of resistance in these production systems. In every instance, the high infection pressure among young (immunity-impaired) transported animals from a diverse geographical origin [17] implicates that in the near future and in these settings antimicrobial therapy remains necessary to minimise economic losses by bacterial infections.

In conclusion, large deviations from registered dosing regimens were found in the veal calves for oral antimicrobial group treatments. A significant association between antimicrobial use and resistance was confirmed for enteric *E*. *coli* and simultaneously demonstrated for respiratory pathogenic *Pasteurellaceae*. The results provide strong evidence that conditions in the veal calf industry are able to generate reservoirs of resistance among both commensal and animal pathogens. Therefore, in our opinion and in accordance with the Dutch surveillance system [25], intensively reared veal calves deserve special attention in antimicrobial resistance monitoring and intervention programmes.

## Supporting Information

S1 File [Antimicrobial use and resistance in cattle–development of a surveillance system at herd level]. Publication for the Federal Public Service for Health, Food Chain Safety, and Environment, Contractual Research, Brussels, Belgium (ISBN 97890558641298).
(PDF)
