# Supplementary material for: Effect of Antimicrobial Consumption and Production Type on Antibacterial Resistance in the Bovine Respiratory and Digestive Tract
Source: PLoS One. 2016 Jan 28;11(1):e0146488. doi: 10.1371/journal.pone.0146488 (PMC4731056; doi:10.1371/journal.pone.0146488)
Supplement: S1 File — Publication for the Federal Public Service for Health, Food Chain Safety, and Environment, Contractual Research, Brussels, Belgium (ISBN 97890558641298). (PDF) [file pone.0146488.s001.pdf]

federale overheidsdienst
VOLKSGEZONDHEID, VEILIGHEID VAN DE VOEDSELKETEN EN LEEFMILIEU

ANTIBIOTICUMGEBRUIK EN ANTIMICROBIËLE RESISTENTIE BIJ RUNDVEE
ONTWIKKELING VAN EEN SURVEILLANCESYSTEEM OP BEDRIJFSNIVEAU

B. Catry, J. Dewulf, G. Opsomer, M. Vanrobaeys,
A. Decostere, F. Haesebrouck, A. de Kruif

Faculteit Diergeneeksunde, Universiteit Gent
DGZ-Vlaanderen v.z.w.

2007

.be
BELGIË

Colofon

Universiteit Gent
Faculteit Diergeneeskunde
Vakgroep Voortplanting, Verloskunde en Bedrijfsdiergeneeskunde
Vakgroep Pathologie, Bacteriologie en Pluimveeziekten
Salisburylaan 133, 9820 MERELBEKE

Dierengezondheidszorg Vlaanderen v.z.w.
Industrielaan 29, 8820 TORHOUT

DEZE PUBLICATIE WERD OPGESTELD IN OPDRDACHT VAN:

FOD VOLKSGEZONDHEID, VEILIGHEID VAN DE VOEDSELKETEN EN LEEFMILIEU
Dienst Contractueel Onderzoek
Zelfbestuurstraat 4, 1070 BRUSSEL

ISBN-NUMMER : 9789058641298

EAN : 9789058641298

Overname van gegevens van deze publicatie voor persoonlijk gebruik is toegestaan mits duidelijke bronvermelding. Elk ander gebruik valt onder de beperkingen van het auteursrecht en hiervoor is de schriftelijke toestemming van de auteurs vereist.

VOORWOORD

Wanneer bacteriën resistent zijn tegen antibiotica, wordt de behandeling van de ziekte die ze veroorzaken, erg bemoeilijkt. Deze resistentie kan bovendien spreiden van dier naar mens. Het is dus belangrijk om niet alleen in de geneeskunde, maar ook in de diergeneeskunde het niveau van antibioticumresistentie zo laag mogelijk te houden.

In de westerse wereld bestaat in de meeste landen een bewakingsprogramma, dat zowel het antibioticumgebruik als het resistentieniveau bij diverse bacteriën registreert. De bekomen gegevens zijn echter niet optimaal, enerzijds door een gebrek aan precieze informatie over het antibioticagebruik, en anderzijds door de beperkingen van de courant gebruikte agar diffusietest bij de bepaling van de antibioticaresistentie.

Voortbouwend op een onderzoek dat in 2002 werd opgestart, had het hier voorgestelde project tot doel om een systeem te ontwikkelen dat toelaat om het antibioticumresistentieniveau op een bedrijf nauwkeurig te bepalen, en maatregelen voor te stellen om de resistentieontwikkeling onder controle te houden. Als model werd hierbij het rundveebedrijf gebruikt, waarbij zowel melkvee-, vleesvee- als vleeskalverenbedrijven in de studie werden betrokken.

In eerste instantie werd een gedetailleerd overzicht van het antibioticumgebruik in Vlaamse rundveebedrijven bekomen. Verder werd accurate informatie verzameld betreffende de antibioticumresistentie aanwezig bij kiemen afkomstig uit het spijsverterings- en ademhalingsstelsel. De aangetroffen bacteriën werden zorgvuldig geïdentificeerd met behulp van moleculaire technieken, om de waarde van de gebruikelijke indicatorbacteriën beter te kunnen inschatten. Daarnaast werden de resistentieprofielen aan het begin en het einde van de productieronde met elkaar vergeleken. Op basis van deze gegevens konden de verbanden tussen het antibioticumgebruik en de aanwezige resistenties worden nagegaan. Tot slot werd er een voorstel van bemonsteringsstrategie ontwikkeld, die moet toelaten op een precieze, efficiënte en betaalbare manier de antibioticaresistentie op een bedrijf te beoordelen.

Dit onderzoek heeft nuttige en waardevolle resultaten opgeleverd voor de resistentiebewakingsprogramma's, waarvan de beschikbare middelen zo doelmatig mogelijk moeten worden ingezet.

Dr. D. VANDEKERCHOVE, MSc, PhD
WETENSCHAPPELIJK ADVISEUR
FOD Volksgezondheid, Veiligheid van de Voedselketen en Leefmilieu
Afdeling Contractueel Onderzoek

Voorwoord
Inhoudstafel
Lijst met afkortingen

1. INLEIDING & DOELSTELLINGEN

2. MONITORING VAN ANTIBIOTICUMGEBRUIK
- 2.1. Studiepopulatie
- 2.2. Gebruiksparameters
- 2.3. Kwantitatieve resultaten
- 2.4. Kwalitatieve resultaten (Dosering)
- 2.5. Discussie

3. MONITORING VAN ANTIBIOTICUMRESISTENTIE
- 3.1. Studiepopulatie
- 3.2. Microbiota van het spijsverteringsstelsel
- 3.3. Microbiota van het ademhalingsstelsel
- 3.4. Discussie

4. ACCURAATHEID VAN RESISTENTIEMONITORING
- 4.1. Bacteriële identificatie
- 4.2. Gevoeligheidsbepalingen
- 4.3. Correcties van resistentiemonitoring in functie van testkarakteristieken
- 4.4. Discussie

5. VERBANDEN TUSSEN ANTIBIOTICUMGEBRUIK EN RESISTENTIE
- 5.1. Studiepopulatie
- 5.2. Het spijsverteringsstelsel: *E. coli*
- 5.3. Het ademhalingsstelsel: *Pasteurellaceae*
- 5.4. Voorkomen van resistentie in twee ecosystemen
- 5.5. Discussie

6. MINIMAAL BEMONSTERINGSPROTOCOL
- 6.1. Reductie van het aantal stalen
- 6.2. Reductie van het aantal te testen antibiotica
- 6.3. Discussie

Samenvatting/Résumé
Referenties
Lijst met publicaties i.v.m. antibioticumgebruik en -resistentie bij rundvee
Bijlage

Lijst met afkortingen

ADD	animal defined daily dose
ARI	antimicrobiële resistentie index
ATC	anatomical therapeutic chemical classification
BAPCOC	*Belgian Antibiotic Policy Coordination Committee*
CA	category agreement
CI	confidentie interval
CLSI	*Clinical and Laboratory Standards Institute*
DDD	defined daily dose
DHS	dihydrostreptomycine
IMM	intramammair
IU	intrauterien
LA	*long acting* (langwerkend)
M.	*Mannheimia*
mE	minor error
ME	major error
MIC	minimale inhibitorische concentratie
MPS	minor pathogene stafylokokken
NCCLS	*National Committee for Clinical Laboratory Standards* (USA)
ND/NC	not determined/calculated (niet berekend)
p	significantie niveau
P.	*Pasteurella*
PBS	phosphate buffered saline
PCR	polymerase chain reaction
PE	parenteraal
PFGE	pulsed-field gel electrophoresis
PO	per os (oraal)
RPV	resistent voorspellende waarde (resistant predictive value)
s.l.	sensu lato
SPV	gevoelig voorspellende waarde (susceptible predictive value)
s.s.	sensu stricto
SD	standaarddeviatie
subsp., ss.	subspecies
tDNA-PCR	tRNA-intergenic spacer PCR
TI	treatment incidence (behandelingsincidentie)
tRNA	transfer ribonucleic acid
UD	unit dose
UDD	used daily dose
VME	very major error
WHO	wereld gezondheidsorganisatie (world health organisation)

1. INLEIDING & DOELSTELLINGEN

De toenemende verworven antibioticumresistentie, zowel in de humane geneeskunde als in de diergeneeskunde, vormt een ernstige bedreiging voor de werking van antibiotica. Therapiefalen, met een soms fatale afloop, maakt dit onderwerp prioritair voor verschillende internationale gezondheidsorganisaties (Bager, 1999; Mevius et al., 2000; Wray en Gnanou, 2000; Avorn et al., 2001). Er wordt tevens melding gemaakt van gelijkenissen tussen de stijging van de antibioticumresistentie bij nutsdieren -de rundveehouderij inbegrepen- en de resistentietoename in de geneeskunde (Schwarz et al., 2001; Catry et al., 2003). Problemen kunnen bij de mens ontstaan door de directe overdracht van resistente zoönotische bacteriën, maar ook door de indirecte overdracht van resistentiegenen naar pathogenen voor de mens. Deze resistentiegenen kunnen afkomstig zijn van dierlijke pathogenen, maar ook van de commensale microbiota (micro-flora) van dieren (Helmuth and Protz, 1997; Witte, 2000; Nollet et al., 2006).

Alhoewel een oorzakelijk verband tussen het antibioticumgebruik en de selectie voor resistentie reeds veelvuldig bewezen werd, is het niet steeds duidelijk in welke mate het gebruik van antibiotica invloed uitoefent op de selectie, verspreiding en persistentie van resistentie. Tegenstrijdigheden in de richtlijnen ter controle van antibioticumresistentie zijn er het gevolg van.

In de meeste Europese landen en Noord-Amerika zijn monitoringprogramma's lopende met als bedoeling de evolutie van resistenties te kunnen volgen. Hierin wordt enerzijds het humaan en veterinair antibioticumgebruik globaal geregistreerd en anderzijds wordt het antibioticumresistentie niveau bepaald bij pathogenen, (commensale) indicatorbacteriën en zoönotische bacteriën (Bager, 1999; Wray & Gnanou, 2000). Indicatorbacteriën zijn kiemen die aanwezig zijn in de commensale micro-flora van dieren, en onder invloed van een antibioticum een resistentie-selectiedruk ondergaan (Dewulf et al., 2006). Hun resistentiepatroon wordt beschouwd als een goede parameter of indicator voor de heersende resistentie-selectiedruk in een bepaalde nutsdierenpopulatie (van den Bogaard & Stobberingh, 2000). Bij de aanvang van dit project waren dergelijke cijfers met betrekking tot de Belgische rundveesector onbestaande. Bovendien worden in de meeste monitoringsprogramma's geen gedetailleerde gegevens over het antibioticumgebruik (o.a. dosering, toedieningswijze en incidentie) bijgehouden, en ook de oorsprong van de onderzochte kiemen (kuddegegevens, o.a. leeftijd van het dier) is niet bekend. Experts zijn overtuigd dat dit gebrek aan gedetailleerde informatie een belangrijke reden is waarom de epidemiologie van antibioticumresistentie niet geheel opgehelderd geraakt (Huovinen, 1999; Bywater, 2000; Mevius et al., 2000).

Daarenboven is over antibioticumresistentie bij rundvee, in vergelijking met bijvoorbeeld de varkenshouderij en pluimveehouderij, zeer weinig bekend. Dit is gedeeltelijk te wijten aan de lage prevalentie bij deze diersoort van fecale *Enterococcus* spp., de internationaal gehanteerde Gram-positieve indicatorbacteriën (Devriese et al., 1992; Wray & Gnanou, 2000). In tegenstelling tot het spijsverteringsstelsel, werd het ademhalingsstelsel bovendien zo goed als verwaarloosd in studies met betrekking tot antibioticumresistentie. Theoretisch vormt de commensale micro-flora van de bovenste ademhalingswegen echter evengoed een reservoir van resistentiegenen (Barbosa & Levy, 2000).

De centrale doelstelling van dit pilootproject was om een gedetailleerd beeld te krijgen van het antibioticumgebruik op Belgische rundveebedrijven, en om het voorkomen van resistentie bij zoönotische, commensale, en facultatief pathogene bacteriën aanwezig in de normale microbiota van runderen te beschrijven. Hierbij werden zowel melkvee-, zoogkoeien- als vleeskalverenbedrijven opgenomen in het onderzoek. Analyses werden verricht teneinde het verband aan te tonen tussen het antibioticumgebruik en het voorkomen van resistentie. Tenslotte werd getracht om een minimaal bemonsteringsprotocol te ontwikkelen, dat moet toelaten om met een minimum aan dieren, stalen en testen een nauwkeurig bewakingssysteem op te bouwen. De resultaten van voorgaande onderzoeken zullen bijgevolg toelaten om adviezen te formuleren betreffende het opzetten van een nationaal monitoringsysteem op rundveebedrijven.

2. MONITORING VAN ANTIBIOTICUMGEBRUIK

2.1. Studiepopulatie en registratieprocedure

Tien zoogkoeienbedrijven en 10 melkveebedrijven werden geselecteerd, voornamelijk uit het klantenbestand van de buitenpraktijk van de Vakgroep Voortplanting, Verloskunde, Bedrijfsdiergeneeskunde van de faculteit diergeneeskunde. Melkveebedrijven met een veestapel van minimaal 40 lacterende dieren, en zoogkoeienbedrijven met jaarlijks minimaal 30 kalvingen, kwamen in aanmerking voor de studie vanwege hun representatief karakter voor de hedendaagse rundveehouderij in België. Daarnaast werden 5 vleeskalverenbedrijven *at random* geselecteerd o.b.v. SANITEL-gegevens (coördinaten veehouder en bedrijfsdierenarts, beslagnummer, bedrijfstype). Bijkomende selectiecriteria waren de bereidheid mee te werken en de stiptheid van zowel de veehouder als de bedrijfsdierenarts. Bij de aanvang van de studie werden supplementaire bedrijfsgegevens verzameld (bedrijfstype, kuddegrootte, managementgegevens).

Gedurende 2 jaar werd het antibioticumgebruik op de melkvee- en vleesvee-bedrijven gedetailleerd bijgehouden aan de hand van een geneesmiddelen-registratiekaart. Op de vleeskalverenbedrijven werd één afmestperiode van 6 maanden gevolgd. Van elke toediening van een antibioticum werden volgende gegevens bijgehouden: de identificatie van het dier, het (geschatte) lichaamsgewicht, de indicatie, de benaming van het toegediende product, de persoon die het product heeft toegediend, de hoeveelheid (ml), de toedieningswijze, de wachttijd, en de datum van toediening.

PROJECT FACULTEIT DIERGENEESKUNDE-BUITENPRAKTIJK

GENEESMIDDELEN – REGISTRATIEKAART MELKVEE

Bedrijf: Naam &Voornaam:……………………… Beslagnummer (SANITEL): …………………
Straat & nr.:……………………………… Bedrijfsdierenarts: …………………………….
Gemeente:……………………………….. Periode van…………….tot……………………

Identificatie dieren (1)	Kg	Vermoedelijke diagnose(*)/ indicatie (2)	Product®	Wie (3)	Wijze (4)	Dosis (ml)	Wachttijd	Datum

(1): Sanitelnummer

(2): 1: mastitis (K/S/D#)
2: diarree
3: longontsteking
4: klauwproblemen
5: andere
(*) bevestigd door een extern laboratorium

(3) M = melker
BD = bedrijfsdierenarts
AD = andere dierenarts
A = andere

(4) IM= intramusculair
IMM = intramammair
SC = subcutaan
PO = per oraal
IV = intraveneus
IP = intraperitoneaal

Noodnummer: 09/2647530

#K: Klinische mastitis, S: subklinische mastitis, D: droogzettherapie

De indicaties werden bijgehouden omdat voor sommige producten de dosering en de behandelingsduur afhankelijk zijn van de indicatie. De wachttijd en de persoon die de behandeling toediende tenslotte, werden respectievelijk opgenomen ter attentie van de veehouder en ter controle van de stiptheid van de studie.

Om de vier maanden werden de geneesmiddelen-registratiekaarten verzameld bij een bezoek aan de bedrijven, en gecontroleerd aan de hand van de wettelijk verplichte toedienings- en verschaffingsdocumenten (bedrijfsdierenarts).

2.2. Gebruiksparameters

2.2.1 Behandelingsincidentie op basis van Animal Defined Daily Dose cattle: $TI_{ADDcattle}$

De $TI_{ADDcattle}$ of het aantal dieren dat dagelijks behandeld wordt met een aanbevolen dosis van een bepaald antibioticum, werd met onderstaande formule berekend (Timmerman et al., 2006):

$$\frac{\text{hoeveelheid geneesmiddel toegediend tijdens observatieperiode (mg)}}{\text{ADDcattle (mg/kg) x observatieperiode (days) x kg cattle at risk}} \text{ x 1000 risicokoeien} = \text{ADDcattle/1000 risicokoeien/ dag} \quad (1)$$

De ADD_{cattle} wordt daarbij gedefinieerd als de aanbevolen gemiddelde onderhoudsdosis per dag van een geneesmiddel voor wat betreft zijn hoofdindicatie uitgedrukt per kg (WHO, 2002a; b). De ADD_{cattle} voor de verschillende antibiotica werden berekend op basis van het compendium (Anoniem 2001; 2003). Deze waarden zijn dus nationale ADD waarden, daar er nog geen internationaal aanvaarde ADD's beschreven zijn in de diergeneeskunde. Als de aanbevolen dosis voor hetzelfde actief bestanddeel verschilde tussen de farmaceutische firma's, dan was de toegekende ADD_{cattle} een gemiddelde van 2 of meer aanbevolen dosissen.

De range van aanbevolen dosissen was beperkt behalve voor ampicilline, amoxicilline, trimethoprim-sulfonamide combinaties en tetracyclines voor intra-uterien gebruik. De toegekende ADD's voor de verschillende antibiotica kan men terugvinden in Tabellen 2-4. De ADD_{cattle} werden uitgedrukt per kg, behalve voor de intramammaire en de intra-uteriene antibiotica. Voor deze preparaten werd gebruik gemaakt van een andere unit, nl. De Unit Dose (UD), waarbij 1 UD overeenkomt met het aantal intramammaire tubes (uitgedrukt per mg) dat per kwartier moet toegediend worden per 24 uur in het geval van mastitisbehandeling, 4 intramammaire tubes (uitgedrukt in mg) bij droogzettherapie en de aanbevolen 24 uur dosis bij intra-uteriene therapie. In geval van combinatie-preparaten werd gewerkt met het hoofdbestanddeel van het preparaat om de ADD_{cattle} te berekenen, met uitzondering van producten die de combinatie trimethoprim-sulfamide bevatten: in dit geval werd de ADD_{cattle} berekend op basis van de hoeveelheid trimethoprim. Voor long-acting preparaten werd de aanbevolen dosis omgezet in een 24 uur dosis.

Voor de melkveebedrijven en de zoogkoeienbedrijven werd de "populatie at risk" gedefinieerd als alle dieren aanwezig op het bedrijf tijdens de observatieperiode. Op de vleeskalverenbedrijven werden enkel de kalveren uit de leeftijdsgroep die opgevolgd werd, geïncorporeerd in de populatie at risk. SANITEL lijsten werden gebruikt om het aantal dieren aanwezig op het bedrijf aan het begin en aan het einde van de observatieperiode te bepalen.

Om vervolgens het aantal kg at risk te berekenen op de melkvee – en zoogkoeienbedrijven, werden de dieren in 4 verschillende leeftijdscategorieën (< 6 maanden, 6-12 maanden, 1-2 jaar, > 2 jaar) verdeeld en het gemiddeld aantal dieren per leeftijdscategorie werd vermenigvuldigd met het gemiddeld gewicht van een dier in die leeftijdscategorie. Dit gemiddeld gewicht werd bepaald aan de hand van specifieke groeitabellen voor Holstein Friesian koeien (Heinrichs en Hargrove, 1987) en Belgisch Wit Blauw (Anoniem, 1994; 1997). Om het aantal kg at risk te bepalen op de vleeskalverenbedrijven, werd het aantal kalveren in 1 leeftijdsgroep vermenigvuldigd met het gemiddeld lichaamsgewicht op moment van behandeling (Chauvin en Madec, 2004), wat 81 kg bedroeg. Aangezien groepsbehandelingen de overgrote meerderheid vormen op deze bedrijven, werden enkel deze behandelingen geïncorporeerd bij het bepalen van het gemiddeld gewicht op moment van behandeling.

2.2.2 Incidentie op basis van Used Daily Dose cattle: $TI_{UDDcattle}$

Doordat zowel het lichaamsgewicht van het dier als de toegediende hoeveelheid product geregistreerd werd, was het tevens mogelijk de werkelijk toegediende dosis te berekenen. Aldus werd de Used Daily Dosis cattle (UDD_{cattle}) bekomen. Waar de ADD_{cattle} gebaseerd is op de registratie van een bepaald product in een bepaald land, geeft de UDD weer hoeveel werkelijk werd toegediend per kg dier. De incidentie gebaseerd op UDD_{cattle} werd als volgt berekend (Timmerman et al., 2006):

$$\frac{\text{hoeveelheid geneesmiddel toegediend tijdens observatieperiode (mg)}}{\text{UDDcattle (mg/kg) x observatieperiode (days) x kg cattle at risk}} \text{ x 1000 risicokoeien} = \text{UDDcattle/1000 risicokoeien/ dag} \quad (2)$$

Het lichaamsgewicht van de behandelde dieren werd door de veehouder geschat of bepaald aan de hand van rasspecifieke groeitabellen (Heinrichs en Hargrove, 1987; Anoniem, 1994; 1997). De proportionele incidenties werden berekend door de $TI_{ADDcattle}$ en de $TI_{UDDcattle}$ van een bepaald antibioticum respectievelijk te delen door de totale $TI_{ADDcattle}$ en totale $TI_{UDDcattle}$ (Grave et al., 1999).

2.2.3 De UDD_{cattle}/ADD_{cattle} ratio en de UDD_{cattle}/UD_{cattle} ratio

De derde en laatste parameter is de UDD_{cattle}/ADD_{cattle} ratio of de UDD_{cattle}/UD_{cattle} ratio. Deze ratio vergelijkt de werkelijk toegediende dosis (UDD_{cattle}) met de aanbevolen dosis. Wanneer men veronderstelt dat correct doseren, doseren is volgens de richtlijnen en het compendium, kan men deze ratio gebruiken om onder– en overdoseren te detecteren. Aangezien er voor sommige producten een grote variatie bestaat in aanbevolen dosis, werd een afwijking van 20% aanvaard als correct gedoseerd. Indien de ratio dus lager was dan 0,8 of hoger dan 1,2 werd besloten dat er respectievelijk onder – en overgedoseerd werd.

2.3. Kwantitatieve resultaten

2.3.1 Inleiding

Op verschillende bedrijven van de oorspronkelijke studiepopulatie bleek het niet mogelijk om gedurende de volledige vooropgestelde observatieperiode het antibioticumgebruik gedetailleerd bij te houden. Redenen hiervoor waren het stopzetten van het bedrijf (2), onnauwkeurigheid inzake rapportering (7), of weigering van de veehouder om zijn medewerking verder te verlenen (1). Uiteindelijk werd op 15 bedrijven het antibioticumgebruik zorgvuldig bijgehouden en/of lieten de externe controlemogelijkheden (toedienings- en verschaffingsdocumenten) toe om de gegevens zorgvuldig aan te vullen. De ATC codering (Anatomical Therapeutic Chemical classification) voor de verschillende gebruikte antibiotica, die door de WHO wordt aangemoedigd om vergelijkingen van gebruikscijfers te standaardiseren (WHO, 2002a), werd eveneens ingesloten in de overzichtstabellen (ATCvet).

2.3.2 Melkveebedrijven

Op de melkveebedrijven werden zowel topicale (intramammaire en intra-uteriene) als parenterale antibiotica regelmatig toegediend (Tabel 1). De totale $TI_{UDcattle}$ voor de intramammaire producten bedroeg 2,8. Dit wil zeggen dat 2,8 koeien per 1000 dagelijks behandeld werden met een standaarddosis van een antibioticum tijdens de observatieperiode. De meest gebruikte intramammaire antibiotica waren beta-lactamase resistente penicillines, alleen of in combinatie met andere antibiotica, en cefalosporines (Tabel 1). De proportionele incidenties bedroegen respectievelijk 27,6%, 29,3% en 27,4%. Voor de behandeling van mastitis werden cefalosporines (38,0%) en beta-lactamase resistente penicillines in combinatie met andere antibiotica (28,9%) het vaakst gebruikt. Voor droogzettherapie werden slechts 2 producten gebruikt: beta-lactamase resistente penicillines alleen (62,0%) of in combinatie met andere antibiotica (38,0%). De totale $TI_{UDcattle}$ voor de intra-uteriene antibiotica bedroeg 0,4. De hoofdindicaties voor het gebruik van intra-uteriene cefalosporines en tetracyclines waren *retentio secundinarum* en *endometritis*.

De totale $TI_{ADDcattle}$ voor de parenterale behandelingen bedroeg 3,0. Wanneer deze incidentie vergeleken wordt met de incidentie gebaseerd op de UDD_{cattle} ($TI_{UDDcattle}$), kunnen we constateren dat in werkelijkheid minder dieren behandeld werden: nl. 2,4 per 1000 in totaal (Tabel 2). De proportionele incidenties voor de meest gebruikte antibiotica waren: nauw-spectrum penicillines (31,2%), nauw-spectrum penicillines in combinatie met andere antibiotica (25,7%), trimethoprim sulfonamide combinaties (15,1%) en cefalosporines (12,1%). Proportionele $TI_{ADDcattle}$ verschilden van de proportionele $TI_{UDDcattle}$. Mastitis was de hoofdindicatie voor het gebruik van nauw-spectrum penicillines, en locomotorische problemen waren de hoofdindicatie voor het gebruik van nauw-spectrum penicillines in combinatie met andere antibiotica.

2.3.2 Zoogkoeienbedrijven

De totale $TI_{ADDcattle}$ op de zoogkoeienbedrijven bedroeg 5,4 terwijl de totale $TI_{UDDcattle}$ 4,9 bedroeg. Op deze bedrijven werden voornamelijk parenterale antibiotica gebruikt. Nauw-spectrum penicillines alleen, of in combinatie met andere antibiotica werden hier het frequentst voor gebruikt (Tabel 2). De proportionele $TI_{ADDcattle}$ bedroegen 70,4% en 23,3% respectievelijk. De hoofdindicatie voor deze produkten was chirurgische prophylaxie tijdens de keizersnede. Fluoroquinolones werden niet frequent gebruikt op dit bedrijfstype (1,3%).

De totale $TI_{ADDcattle}$ voor de topicale behandelingen bedroeg 0,9. Wanneer we deze waarde vergelijken met de $TI_{UDDcattle}$ zien we dat in werkelijkheid iets meer dieren behandeld werden, nl. 1,2 per 1000 in totaal. Tetracyclines waren de meest gebruikte topicale antibiotica (88,7%). De hoofdindicatie was chirurgische prophylaxie bij de keizersnede, maar ze werden ook gebruikt voor de behandeling van *retentio secundinarum.* Intramammaire antibiotica werden niet frequent gebruikt op de zoogkoeienbedrijven. De $TI_{UDcattle}$ bedroeg 0,1 (Tabel 1). Oraal werd er behandeld met colistine (92,2%) en trimethoprim sulfonamide combinaties (7,8%) ter preventie van neonatale diarree bij kalveren.

2.3.3 Vleeskalverenbedrijven

Op de vleeskalverenbedrijven maken de groepsbehandelingen het grootste deel uit van het antibioticumgebruik. De totale $TI_{ADDcattle}$ voor de oraal toegediende antibiotica bedroeg 97,8. Maar in werkelijkheid werden veel meer kalveren oraal behandeld daar de $TI_{UDDcattle}$ 266,8 bedroeg (Tabel 3). Tetracyclines (54,2%) en tylosine (24,2%) werden het frequentst gebruikt. De hoofdindicatie voor het gebruik van deze producten was de prophylactische of metaphylactische behandeling van ademhalingsproblemen. Op 1 vleeskalverenbedrijf werd tylosine ook gebruikt voor de preventie van *Clostridium prefringens* enterotoxemie. De duur van de orale behandelingen varieerde tussen de 5 en 13 dagen, maar in 2 gevallen werd tylosine gedurende 41 en 73 dagen toegediend. Een groot deel van deze orale behandelingen werd toegediend als startkuur (23,0%). Colistine (55,2%), oxytetracycline (29,5%) en trimethoprim sulfonamide combinaties (10,1%) werden het frequentst gebruikt als startkuur. De duur van deze startkuren varieerde tussen de 6 en 13 dagen en ze werden op alle vleeskalverenbedrijven toegepast.

Voor de parenterale antibiotica was de $TI_{ADDcattle}$ veel lager, nl. 6,7. De proportionele $TI_{ADDcattle}$ voor de meest gebruikte antibiotica waren: cefalosporines (43,4%), fluoroquinolones (15,6%), macroliden (12,9%) en lincosamiden (12,8%). De indicatie voor het gebruik van cefalosporines op deze bedrijven betrof ademhalingsproblemen.

Tabel 1. Incidentie ($TI_{UDcattle}$ en $TI_{UDDcattle}$) (min-max) van de topicaal toegediende antibiotica op de verschillende bedrijfstypes.

Actieve substantie	ATCvet[a]	Indicatie[b]	UD (mg)	Melkveebedrijven		Zoogkoeienbedrijven	
				$TI_{UDcattle}$	$TI_{UDDcattle}$	$TI_{UDcattle}$	$TI_{UDDcattle}$
**INTRAMAMMAIRE ANTIBIOTICA**							
Procaine penicilline+ neomycine	QJ51RC	M	500	0.07 (0.00-0.43)	0.03 (0.00-0.22)	_	_
Cloxacilline	QJ51CF	M	100	0.23 (0.00-1.14)	0.06 (0.00-0.31)	_	_
Cloxacilline	QJ51CF	D	2200	0.55 (0.00-1.11)	0.55 (0.00-1.11)	_	_
Nafcilline+benzylpenicilline+DHS[c]	NA[d]	M	100	0.59 (0.00-1.42)	0.45 (0.00-1.21)	0.06 (0.00-0.49)	0.06[e] (0.00-0.49)
Nafcilline+benzylpenicilline+DHS	NA	D	400	0.24 (0.00-1.22)	0.24 (0.00-1.22)	_	_
Cefquinome	QJ51DA	M	150	0.43 (0.00-1.02)	0.60 (0.00-0.97)	_	_
Cefalexine	QJ51DA	M	400	0.35 (0.00-2.05)	0.39 (0.00-2.31)	_	_
Lincomycine+neomycine	QJ51RF	M	660	0.17 (0.00-0.43)	0.29 (0.00-0.71)	0.03 (0.00-0.30)	0.03[e] (0.00-0.30)
Pirlimycine	QJ51FF	M	50	0.21 (0.00-1.03)	0.10 (0.00-0.51)	_	_
**Totaal**				**2.83** (1.81-3.70)	**2.71** (0.94-3.83)	**0.09** (0.00-0.79)	**0.09** (0.00-0.79)
**INTRA-UTERIENE ANTIBIOTICA**							
Cefapirine	QG01DA	V	500	0.17 (0.00-0.78)	0.16 (0.00-0.77)	<0.01 (0.0-0.02)	<0.01 (0.0-0.02)
Chlortetracycline hydrochloride	QG01AA	V	1500	0.25 (0.00-0.52)	0.16 (0.00-0.34)	0.78 (0.36-1.08)	1.07 (0.39-1.62)
**Totaal**				**0.42** (0.00-1.27)	**0.32** (0.00-1.11)	**0.78** (0.38-1.08)	**1.07** (0.39-1.62)
**TOTAAL**				**3.25**	**3.03**	**0.87**	**1.16**

[a]ATCvet: *anatomical therapeutic chemical* klassificatie voor veterinaire produkten (WHO, 2002b), [b]M: mastitis; D droogzettherapie; V: variable (prophylactisch en/of therapeutisch), [c]DHS: dihydrostreptomycine, [d] NA: niet beschikbaar (not available), [e] UDD kon niet berekend worden. Daarom werd de TI_{UD} waarde gebruikt om de totale TI_{UDD} te berekenen.

Tabel 2. Incidentie (**$TI_{ADDcattle}$** en **$TI_{UDDcattle}$**) (min.-max) van de parenteraal toegediende antibiotica op de verschillende bedrijfstypes.

**Actieve substantie**	**ATCvet**[a]	**ADD**	**Melkveebedrijf**		**Zoogkoeienbedrijf**		**Vleeskalverenbedrijf**	
		**(mg/kg)**	**$TI_{ADDcattle}$**	**$TI_{UDDcattle}$**	**$TI_{ADDcattle}$**	**$TI_{UDDcattle}$**	**$TI_{ADDcattle}$**	**$TI_{UDDcattle}$**
Penethamaat hydroiodide	QJ01CE	12.5	0.04 (0.00-0.23)	0.03 (0.00-0.18)	–	–	–	–
Procaine benzylpenicilline	QJ01CE	12	0.58 (0.00-1.63)	0.15 (0.00-0.27)	2.12 (0.00-4.39)	0.87 (0.00-1.70)	–	–
Procaine+benzathine benzylpenicilline LA	QJ01CE	3	0.32 (0.00-0.72)	0.17 (0.00-0.38)	0.54 (0.00-1.35)	0.29 (0.00-0.79)	–	–
Procaine benzylpenicilline+DHS	QJ01RC	8	0.52 (0.00-3.24)	0.25 (0.00-1.58)	0.04 (0.00-0.42)	0.02 (0.00-0.12)	0.04 (0.00-0.30)	0.01 (0.00-0.10)
Procaine benzylpenicilline+neomycine	QJ01RC	10	0.25 (0.00-0.99)	0.21 (0.00-0.96)	0.84 (0.03-1.94)	0.74 (0.03-1.82)	0.05 (0.00-0.28)	0.03 (0.00-0.17)
Ampicilline	QJ01CA	28	<0.01 (<0.01)	<0.01 (<0.01)	<0.01 (<0.01)	<0.01 (<0.01)	–	–
Ampicilline LA	QJ01CA	11.25	<0.01 (<0.01)	<0.01 (0.00-0.08)	<0.01 (<0.01)	<0.01 (<0.01)	–	–
Amoxicilline	QJ01CA	9	<0.01 (<0.01)	<0.01 (<0.01)	<0.01 (0.00-0.01)	<0.01 (<0.01)	0.09 (0.00-0.22)	0.06 (0.00-0.14)
Amoxicilline LA	QJ01CA	6.25	–	–	–	–	0.32 (0.00-0.71)	0.28 (0.00-0.72)
Amoxicilline+ clavulaanzuur	QJ01CA	7	–	–	0.05 (0.00-0.15)	0.04 (0.00-0.12)	–	–
Ceftiofur	QJ01DA	1	0.30 (0.00-0.81)	0.15 (0.00-0.34)	0.01 (0.00-0.05)	0.01 (0.00-0.05)	2.32 (0.00-5.79)	1.27 (0.00-3.07)
Cefquinome	QJ01DA	1	0.06 (0.00-0.19)	0.04 (0.00-0.13)	–	–	0.60 (0.00-3.14)	0.60 (0.00-3.19)
Tylosine	QJ01FA	7.5	–	–	–	–	0.23 (0.00-1.30)	0.15 (0.00-0.83)
Tilmicosine	QJ01FA	10	–	–	0.03 (0.00-0.14)	0.03 (0.00-0.11)	0.64 (0.00-2.14)	0.64 (0.00-2.07)
Lincomycine	QJ01FF	10	–	–	0.02 (0.00-0.05)	0.06 (0.00-0.19)	–	–
Lincomycine+spectinomycine	QJ01FF	5	0.01 (0.00-0.02)	<0.01 (0.00-0.04)	0.03 (0.00-0.27)	0.02 (0.00-0.18)	0.86 (0.00-2.41)	0.87 (0.00-2.76)
Gentamicine	QJ01GB	3.75	0.03 (0.00-0.14)	0.04 (0.00-0.17)	–	–	0.02 (0.00-0.11)	0.03 (0.00-0.16)
Trimethoprim+sulpha	QJ01EW	4.25	0.46 (0.00-1.74)	0.84 (0.00-3.46)	0.04 (0.00-0.11)	0.05 (0.00-0.18)	–	–
Trimethoprim+sulpha LA	QJ01EW	1.2	–	–	–	–	0.29 (0.00-1.63)	0.29 (0.00-1.66)
Oxytetracycline	QJ01AA	3	0.13 (0.00-0.24)	0.08 (0.00-0.22)	<0.01 (0.00-0.02)	<0.01 (0.00-0.16)	–	–
Enrofloxacine	QJ01MA	2.5	0.25 (0.00-1.54)	0.21 (0.00-1.45)	<0.01 (0.00-0.02)	<0.01 (0.00-0.06)	0.78 (0.00-2.16)	0.52 (0.00-2.08)

Danofloxacine	QJ01MA	1.25	0.01 (0.00-0.03)	<0.01 (0.00-0.02)	0.05 (0.00-0.19)	0.05 (0.00-0.19)	0.18 (0.00-0.50)	0.09 (0.00-0.33)
Marbofloxacine	QJ01MA	2	–	–	–	–	0.09 (0.00-0.25)	0.05 (0.00-0.28)
Florfenicol	QJ01BA	10	0.03 (0.00-0.08)	<0.01 (0.00-0.02)	–	–	0.22 (0.00-1.26)	0.30 (0.00-1.68)
Colistine	QJ01XB	50	–	–	–	–	0.01 (0.00-0.04)	0.02 (0.00-0.04)
**Totaal**			**3.01**	**2.36**	**3.78**	**2.15**	**6.74**	**5.21**

[a]ATCvet: *anatomical therapeutic chemical* klassificatie voor veterinaire produkten (WHO, 2002b).

Tabel 3. Incidentie ($TI_{DDDcattle}$ en $TI_{UDDcattle}$) (min-max) van de oraal toegediende antibiotica op de vleeskalverenbedrijven

**Actieve substantie**	**ATCvet[a]**	**ADD_{cattle} (mg/kg)**	**Vleeskalverenbedrijven**	
			**$TI_{ADDcattle}$**	**$TI_{UDDcattle}$**
Amoxicillin	QJ01CA	20	3.69 (0.00-32.75)	12.00 (0.00-106.71)
Tylosin	QJ01FA	4	23.61 (0.00-179.52)	121.72 (0.00-393.80)
Trimethoprim+sulphonamide	QJ01EW	30	2.57 (0.00-5.04)	17.01 (0.00-28.34)
Oxytetracycline	QJ01AA	40	38.05 (7.56-133.53)	59.62 (12.85-143.63)
Doxycycline	QJ01AA	10	14.92 (0.00-40.94)	29.94 (0.00-76.70)
Colistin	QJ01XB	4.6	13.75 (0.00-35.60)	22.95 (0.00-40.94)
Flumequine	QJ01MA	18	1.19 (0.00-13.65)	3.57 (0.00-40.94)
**TOTAAL**			**97.78**	**266.81**

[a]ATCvet: *anatomical therapeutic chemical* klassificatie voor veterinaire produkten (WHO, 2002b).

2.4. Kwalitatieve resultaten (Dosering)

2.4.1 Melkveebedrijven

In het algemeen werd 24,7% van de parenterale behandelingen op de melkveebedrijven correct gedoseerd. De overige behandelingen werden onder- of overgedoseerd in respectievelijk 45,0% en 30,3% van de gevallen. Zo werden bijvoorbeeld nauwspectrum penicillines alleen, in combinatie met andere antibiotica, en cefalosporines overgedoseerd in respectievelijk 83,1%, 52,8% en 76,0% van de gevallen. Dit wordt aangegeven door de gemiddelde UDD/ADD ratio die voor deze producten hoger is dan 1,2 (Tabel 4). Deze producten werden correct gedoseerd in respectievelijk 13,6%, 45,1% en 19,4% van de gevallen. Trimethoprim sulfonamide combinaties werden voornamelijk

ondergedoseerd (95,3%), terwijl de quinolones in 53,5% van de gevallen correct gedoseerd werden.

De intra-uteriene preparaten werden op de melkveebedrijven in 48,2% van de gevallen correct gedoseerd. De overige intra-uteriene behandelingen werden onder-en overgedoseerd in respectievelijk 14,3% en 37,5% van de gevallen. Tweeënveertig procent van de intramammaire mastitisbehandelingen werd correct gedoseerd. Over-en onderdosering trad op in respectievelijk 19,9% en 38,7% van de gevallen. Cefalosporines voor de intramammaire behandeling van mastitis werden voornamelijk ondergedoseerd (50,0%) of correct gedoseerd (44,6%). Ook beta-lactamase resistente penicillines voor de behandeling van mastitis werden correct gedoseerd in 71,8% van de gevallen. In het geval van droogzettherapie was de UDDcattle gelijk aan de UDcattle. Dit resulteerde in een UDD/UD ratio van 1 (Tabel 5).

2.4.2 Zoogkoeienbedrijven

In het algemeen werden parenterale antibiotica correct gedoseerd in 40,9% van de gevallen. Over-en onderdosering trad op in respectievelijk 51,4% en 7,7% van de gevallen. Een grote meerderheid van de inta-uteriene antibiotica werd ondergedoseerd, nl. 90,2%. Overdosering van deze producten gebeurde eerder zelden (9,8%).

Nauw-spectrum penicillines voor parenteraal gebruik, de meest gebruikte antibiotica op de zoogkoeienbedrijven, werden voornamelijk overgedoseerd (86,6%). Injecteerbare nauw-spectrum penicillines in combinatie met andere antibiotica werden correct gedoseerd in 79,5% van de gevallen. Onder-en overdosering van dit laatste antibioticum gebeurde veel minder frequent, namelijk in respectievelijk 4,9% en 15,6% van de gevallen.

2.4.3 Vleeskalverenbedrijven

Op deze bedrijven werd 88,0% van de oraal toegediende antibiotica ondergedoseerd. Slechts 10,7% werd correct gedoseerd en 1,3% werd overgedoseerd. Enkel tetracyclines en colistine werden correct gedoseerd in respectievelijk 16,6% en 36,7% van de gevallen. Overdosering van tetracyclines trad op in 3,3% van de gevallen.

Onderdosering van parenteraal toegediende antibiotica gebeurde veel minder frequent (11,4%) dan overdosering (42,6%). Zesenveertig procent van de parenterale behandelingen op vleeskalverenbedrijven werd correct gedoseerd. Injecteerbare macroliden en lincosamiden bijvoorbeeld werden correct gedoseerd in meer dan 50% van de gevallen (repectievelijk 53,3% en 59,5%). Cefalosporines en quinolones werden voornamelijk overgedoseerd (respectievelijk 62,2% en 53,9%)

Tabel 4. Gemiddelde UDDcattle/ADDcattle ratio’s (min-max) voor de orale en de parenterale behandelingen op de verschillende bedrijfstypes.

Actieve substantie	ATCvet[a]	Melkvee-bedrijven	Zoogkoeien-bedrijven	Vleeskalverenbedrijven	
		Parenteraal	Parenteraal	Parenteraal	Oraal
Penethamate hydroiodide	QJ01CE	1.28 (1.23-1.33)	_	_	_
Procaïne benzylpenicilline	QJ01CE	1.93 (0.71-8.33)	2.48 (0.42-6.00)	_	_

Procaïne+benzathine benzylpenicilline LA	QJ01CE	1.89 (1.25-2.40)	1.69 (0.34-3.57)	_	_
Procaïne benzylpenicilline+DHS	QJ01RC	2.05 (1.07-3.13)	2.43 (0.30-5.00)	2.94 (2.74-3.00)	_
Procaïne benzylpenicilline+neomycine	QJ01RC	1.19 (0.67-8.00)	1.12 (0.46-3.38)	1.66 (1.37-1.94)	_
Ampicillin LA	QJ01CA	0.06 [b]	0.44 [b]	_	_
Amoxicillin	QJ01CA	1.67 [b]	2.33 [b]	1.60 (1.39-1.90)	0.31 (0.03-0.69)
Amoxicillin LA	QJ01CA	_	_	1.17 (0.72-3.64)	_
Amoxicillin+clavulanic acid	QJ01CA	_	1.39 (1.00-2.00)	_	_
Ceftiofur	QJ01DA	1.98 (1.00-3.33)	_	1.60 (0.95-2.63)	_
Cefquinome	QJ01DA	1.50 (1.251.67)	_	1.03 (0.86-2.88)	_
Tilmicosin	QJ01FA	_	1.95 (0.81-12.00)	1.00 (0.69-1.14)	_
Tylosin	QJ01FA	_	_	1.57 [b]	0.25 (0.05-0.59)
Gentamicin	QJ01GB	0.82 (0.44-1.33)	_	0.67 (0.57-0.80)	_
Lincomycine+spectinomycine	QJ01FF	2.22 (0.22-4.00)	1.50 (0.75-4.00)	0.99 (0.43-1.68)	_
Trimethoprim+sulfonamide	QJ01EW	0.55 (0.31-0.94)	1.03 (0.54-1.51)	0.98 [b]	0.22 (0.07-0.33)
Oxytetracycline	QJ01AA	1.64 (1.11-3.33)	1.77 (0.11-6.67)	_	0.66 (0.25-1.33)
Doxycyclin	QJ01AA	_	_	_	0.51 (0.25-0.63)
Flumequine	QJ01MA	_	_	_	0..33*
Enrofloxacine	QJ01MA	1.20 (0.80-4.00)	0.36 [b]	1.41 (0.42-4.54)	_
Danofloxacine	QJ01MA	1.58 (1.00-2.00)	_	1.52[b]	_
Marbofloxacine	QJ01MA	_	_	0.94 (0.80-1.03)	_
Florfenicol	QJ01BA	5.11 (5.00-5.25)	_	0.75 (0.29-0.86)	_
Colistin	QJ01XB	_	_	0.98 (0.66-1.114)	0.66 (0.42-1.04)

[a]ATCvet: *anatomical therapeutic chemical* klassificatie voor veterinaire produkten (WHO, 2002b), [b] geen min-max. Deze antibiotica werden slechts eenmalig toegediend of verschillende keren aan dezelfde dosis.

Tabel 5. Gemiddelde UDDcattle/UDcattle ratio's (min-max) voor de topicale antibiotica op de verschillende bedrijfstypes

**Actieve substance**	**Indicatie**	**Melkveebedrijven**		**Zoogkoeienbedrijven**	
		Intramammair	Intra-uterien	Intramammair	Intra-uterien
Procaine penicillin+ neomycin	M[b]	2.00 [a]	–	–	–
Cloxacillin	M[b]	3.64 (2.00-4.00)	–	–	–
Cloxacillin	D [c]	1.00 [a]	–	–	–
Nafcillin+benzylpenicillin+DHS	M[b]	1.29 (1.00-2.00)	–	ND[e]	–
Nafcillin+benzylpenicillin+DHS	D [c]	1.00 [a]	–	–	–
Cefquinome	M[b]	0.71 (0.50-1.50)	–	–	–
Cefalexin	M[b]	0.90 (0.50-1.00)	–	–	–
Cefapirine	V [d]	–	1.04 (1.00-2.00)	–	1.00 [a]
Lincomycin+neomycin	M[b]	0.58 (0.50-1.00)	–	ND[e]	–
Pirlimycin	M[b]	2.00 [a]	–	–	–
Chlortetracycline hydrochloride	V [d]	–	1.57 (0.67-2.00)	–	0.73 (0.67-0.90)

[a] geen min-max. Deze antibiotica werden slechts eenmalig toegediend of verschillende keren aan dezelfde dosis, [b] mastitis; [c] droogzettherapie; [d] variabel (prophylactische en therapeutische intra-uteriene behandeling), [e] UDD/UD kon niet berekend worden

2.5. Discussie

2.5.1 Methodologie

Er werden verschillende veranderingen doorgevoerd aan de DDD zoals deze geïntroduceerd werd in de diergeneeskunde door Grave et al. (1999). In het verleden werd de DDD gedefinieerd als een meeteenheid uitgedrukt voor een koe van 500 kg. In deze studie werd de ADD uitgedrukt per kg zoals vooropgesteld door de WHO (2002a; b). Het gevolg van deze aanpassing is dat het aantal dieren at risk in de formule van Grave et al. (1999) vervangen wordt door het aantal kg dier at risk (kg). Het aantal dieren at risk werd in de studie van Grave et al. (1999) gedefinieerd als louter de totale populatie lacterende dieren. Omdat echter alle individuën binnen een omschreven omgeving, bijvoorbeeld een bedrijf, ecologisch verbonden zijn en daardoor theoretisch aan een zelfde selectiedruk onderhevig zijn (Barbosa & Levy, 2000), werden alle dieren inclusief de kalveren aanwezig op het bedrijf beschouwd als de populatie at risk. De enige uitzondering hierop waren de vleeskalverenbedrijven. Op deze bedrijven zijn vaak verschillende leeftijdsgroepen aanwezig die apart gehuisvest worden. Daarom werden enkel de kalveren van de leeftijdsgroep die gevolgd werd van het begin tot het einde van de productieronde geïncorporeerd in de berekeningen omdat de verschillende productierondes als onafhankelijk van elkaar werden beschouwd.

Een derde verandering die gemaakt werd, is het gebruik van de Unit Dose en deze is gebaseerd op de aanbevelingen van een recent WHO verslag (WHO, 2002b). De Unit Dose werd toegepast om het gebruik van intramammaire en intra-uteriene antibiotica te beschrijven. Bij deze antibiotica is de dosis namelijk onafhankelijk van het lichaamsgewicht en kon de ADD dus niet uitgedrukt worden per kg. In de studie van Grave et al. (1999) werd 1 intramammaire tube gedefinieerd als 1 DDD_{cow}. Maar in het geval van droogzettherapie, die algemeen wordt toegepast in België, klopt dit niet omdat er telkens 4 tubes per koe worden toegediend. In de studie van Grave et al. (1999) vormde dit geen probleem, omdat prophylactisch gebruik van antibiotica bij droogzettherapie niet toegestaan is in Scandinavië. Maar ook voor mastitis klopte de eerdere definitie van Grave et al. (1999) niet, omdat 1 tube niet voor alle producten de aanbevolen 24 uur dosis is. Daarom werd de UD_{cattle} gedefinieerd als het aantal tubes, uitgedrukt in mg, aanbevolen per 24 uur.

Omdat de ADD niet noodzakelijk overeenkomt met de werkelijk toegediende dosis, werd deze unit reeds verfijnd tot de Prescribed Daily Dose (Chauvin et al., 2001). Een nadeel van dit laatste systeem is dat niet alle voorgeschreven antibiotica ook werkelijk gebruikt worden en soms verschilt de voorgeschreven dosis van de werkelijk gebruikte dosis. Daarom werd de Used Daily Dose geïntroduceerd. Informatie om tot deze UDD te komen is moeilijker te bekomen en is meestal niet beschikbaar voor een ganse populatie op lange termijn, maar op bedrijfsniveau geeft deze nieuwe unit veel meer accurate en gedetailleerde informatie die nodig is om het verband tussen het antibioticumgebruik en antibioticumresistentie te onderzoeken.

2.5.2 Consumptiedata

In België gebeurt de verdeling van diergeneesmiddelen voor landbouwhuisdieren voornamelijk door de dierenartsen. Bijgevolg bekomen de veehouders hun diergeneesmiddelen rechtstreeks van de dierenarts en mogen zij hun dieren zelf behandelen onder toezicht van de bedrijfsdierenarts. Op de deelnemende bedrijven werden er beperkte geneesmiddelenregisters bijgehouden om een kwaliteitslabel voor hun melk of vlees te bekomen.

Globaal gezien werden er veel verschillende antibiotica gebruikt op alle bedrijfstypes. Alle toegediende antibiotica waren geregistreerd voor gebruik bij het rund, met uitzondering van lincomycine voor parenterale toediening. Dit product werd op 2 bedrijven gebruikt voor de intraperitoneale behandeling van peritonitis, alhoewel het enkel geregistreerd is voor de behandeling van infecties met gram-positieve en anaërobe kiemen bij varkens. Ook Kaneene en Miller (1992) observeerden off-label gebruik van lincomycine bij rundvee.

Bovendien werden veel verschillen waargenomen tussen bedrijven en de verschillende bedrijfstypes, zowel wat betreft de types antibiotica die gebruikt werden als wat betreft de hoeveelheid. Dit zou te wijten kunnen zijn aan verschillen in voorkeur voor bepaalde producten tussen dierenartsenpraktijken. Anderzijds kunnen ook verschillen in management en andere bedrijfsspecifieke factoren aan de basis liggen van het verschil in antibioticumgebruik tussen de bedrijven (Nielsen et al., 2002; Chauvin en Madec, 2004). Op vleeskalverenbedrijven bijvoorbeeld, worden veel kalveren met verschillende achtergrond van verschillende bedrijven samengebracht op 1 bedrijf. Het gevolg van dit soort productiesysteem is een hoge infectiedruk en bijgevolg een hoog gebruik van antibiotica, vnl. prophylactisch. Problemen die vaak ontstaan bij voedermedicatie of medicatie via het drinkwater of de melk, zijn gebrek aan een bacteriologische diagnose, lage doseringen en langdurige blootstelling aan antibiotica van een groot aantal dieren (Ungemach, 2000; Schwarz et al., 2001). Ook in deze studie werd een groot deel van de orale prophylactische behandelingen (80%) ondergedoseerd.

Prophylaxie werd niet alleen toegepast bij vleeskalveren, maar ook op de andere bedrijfstypes. Op de zoogkoeienbedrijven bijvoorbeeld, worden antibiotica vaak prophylactisch toegediend tijdens de keizersnede aan Belgisch Witblauwe koeien. Bij dit ras wordt keizersnede uitgevoerd in meer dan 90% van de verlossingen (Coopman et al., 2000; 2004). Meestal wordt er dan een standaarddosis van 12.000 tot 30.000 mg nauw-spectrum penicillines toegediend. Bij melkvee worden antibiotica vaak prophylactisch gebruikt om koeien droog te zetten teneinde eventuele subklinische infecties te genezen en ter preventie van nieuwe infecties. In tegenstelling tot sommige Scandinavische landen wordt droogzettherapie algemeen toegepast op Belgische melkveebedrijven. In Denemarken moet de behandeling met een long acting antibioticum bij het droogzetten, gebaseerd zijn op een individuele diagnose van de dierenarts op uierniveau, een positieve bacteriologische cultuur, of een mastitisbehandeling binnen de 30 dagen voor het droogzetten (Bennedsgaard, 2003). In België wordt deze procedure wel aangemoedigd, maar is ze niet verplicht.

Een belangrijke parameter die werd berekend was de UDD/ADD ratio. Deze methode om na te gaan of er correct gedoseerd werd, heeft ook een belangrijke beperking. Theoretisch gezien kan een gemiddelde UDD/ADD ratio van 2 toegediende antibiotica gelijk zijn aan 1, als een toediening voor 50% wordt ondergedoseerd en de andere voor 50% wordt overgedoseerd. Daarom werd ook het percentage behandelingen dat correct onder- of overgedoseerd werd, bepaald voor de belangrijkste produkten.

De verschillen tussen de ADD_{cattle} en de UDD_{cattle} waren voor sommige producten zeer uitgesproken, vnl. voor de orale formulaties op de vleeskalverenbedrijven. Discrepanties tussen de $TI_{ADDcattle}$ en $TI_{UDDcattle}$ waren het gevolg van incorrect doseren. Onvoldoende advies van de dierenarts, verkeerde interpretatie van de bijsluiter en gebruik van een standaardtherapie die niet gebaseerd is op een correcte schatting van het lichaamsgewicht, zijn mogelijke verklaringen voor deze discrepanties. Ze kunnen ook ontstaan wanneer aanbevolen doseringen verschillend zijn voor de verschillende indicaties van een product, of voor de behandeling van een ernstige versus matige ziekte, of voor de behandeling van een jong versus een volwassen dier. Daarom werd een deviatie van 20% van 1 nog beschouwd als correct gedoseerd.

Voor sommige antibiotica bestaan er relatief grote verschillen tussen de aanbevolen dosissen naargelang de farmaceutische firma's. Voor intra-uteriene tetracyclines bijvoorbeeld, varieert de aanbevolen dosis tussen een tablet van 1000 mg en een tablet van 2000 mg. Dit resulteert in een UD_{cattle} van 1500mg, met andere woorden een dosis die zelden of nooit wordt toegediend. Dit heeft dus gevolgen bij de berekening van de UDD/UD ratio. Zelfs wanneer de aanbevelingen van de bijsluiter gevolgd worden, zal de UDD/UD ratio nooit tussen 0.8 en 1.2 liggen. De interpretatie van de UDD/UD ratio's van de intra-uteriene tetracyclines en de andere producten met een wijde range van aanbevolen dosissen moet dus met de nodige voorzichtigheid gebeuren. Verder heeft ook de indicatie (prophylactisch of curatief) een grote invloed op de aanbevolen dosis, vnl. op de dosis van intra-uteriene antibiotica. In het geval van chirurgische prophylaxie bij de keizersnede wordt meestal 1 tablet van 1000 mg tetracycline toegediend, terwijl bij de behandeling van retentio secundinarum 2 of meerdere tabletten worden toegediend. Er kan dus besloten worden dat data betreffende de indicatie essentieel zijn voor de interpretatie van de UDD/UD of de UDD/ADD ratio's.

Incidenties zijn moeilijk te vergelijken met andere studies omwille van de verschillen in methodologie. Bijgevolg kunnen enkel de proportionele incidenties vergeleken worden. In de studie van Grave et al. (1999) waren nauw-spectrum penicillines in combinatie met andere antibiotica de meest frequent gebruikte intramammaire antibiotica. De proportionele incidentie voor deze antibiotica bedroeg 90% in Zweden en 100% in Noorwegen, terwijl ze in onze studie maar 2,7% was. Beta lactamase resistente penicillines in combinatie met andere antibiotica (30,9%), cephalosporines (29,0%) en beta lactamase resistente penicillines (27,1%) waren de meest gebruikte intramammaire antibiotica in deze studie. Een verklaring voor dit verschil is mogelijk het niveau van penicilline resistentie van *Staphylococcus aureus*. In Noorwegen en Zweden zijn de resistentieniveaus voldoende laag (10-15%) om penicilline te gebruiken als eerste keuze antibioticum bij de behandeling van mastitis (Aarestrup en Jensen, 1998). In België daarentegen is het niveau van penicilline resistentie beduidend hoger, nl. ongeveer 50% (Devriese et al., 1997).

We kunnen concluderen dat in deze studie voor het eerst data betreffende het antibioticumgebruik in de rundveesector voorgesteld worden aan de hand van de UDD methodologie. Er werden grote verschillen gevonden tussen de verschillende bedrijven en de verschillende bedrijfstypes, zowel wat betreft het type antibioticum dat gebruikt werd, als in de gebruikte hoeveelheid en dosis. Voor verschillende antibiotica werden grote verschillen waargenomen in de aanbevolen dosis. De impact van deze verschillen op de ontwikkeling van antibioticumresistentie wordt onderzocht in een volgend onderdeel.

3. MONITORING VAN ANTIBIOTICUMRESISTENTIE

3.1. Studiepopulatie

Het objectief van dit onderdeel van de veldstudie was de antibioticumresistentie op rundveebedrijven op te volgen in de tijd, om zo de dynamiek van de resistentiesituatie op bedrijfsniveau te achterhalen. Deze informatie werd dan later gekoppeld aan het antibioticumgebruik op de bedrijven om het verband tussen het antibioticumgebruik en de antibioticumresistentie na te gaan. Om de continuïteit van de resistentiebepalingen op de verschillende bedrijven te waarborgen, werd daarbij gebruik gemaakt van commensale bacteriën voor het ecosysteem van het spijsverteringsstelsel en facultatief pathogene bacteriën voor het ecosysteem van het ademhalingsstelsel. Naast de (potentieel) pathogene bacteriën, ondervinden immers ook de bacteriën behorend tot de normale micro-flora een resistentie-selectiedruk ten gevolge van het gebruik van antimicrobiële middelen. De commensale bacteriën werden 'indicatorbacteriën' genoemd. Zij worden gebruikt voor de evaluatie van de antibioticumresistentie op de verschillende bedrijven. Vandaar dat de studiepopulatie voor het opmeten van het antibioticumgebruik en het opmeten van de antibioticumresistentie, oorspronkelijk dezelfde 25 rundveebedrijven van de antibioticumregistratie omvatte, m.n. 10 melkvee-, 10 vleesvee-, en 5 vleeskalverenbedrijven.

3.2 Microbiota van het spijsverteringsstelsel

<u>3.2.1 Inleiding</u>

In nationale resistentie-monitoringprogramma's worden zowel data opgenomen aangaande het antibioticumgebruik als aangaande het resistentieniveau van zowel pathogene, zoönotische als indicatorbacteriën (Bager, 1999; Wray & Gnanou, 2000). Indicatorbacteriën zijn kiemen die geïsoleerd worden uit de feces van gezonde dieren om het reservoir van mogelijk overdraagbare resistentiegenen in de bacteriële populatie in te schatten. *Escherichia coli* en enterokokken (*Enterococcus (E.) faecalis* and *E. faecium)* werden respectievelijk gekozen als Gram negatieve en Gram positieve indicatorbacteriën, omwille van de hoge prevalentie in de feces van gezonde dieren en omwille van het feit dat ze ontvankelijk zijn voor resistentiegenen (Catry et al., 2003; Helmuth and Protz, 1997; Witte, 2000). Verder moeten indicatorkiemen ook voorkomen in het spijsverteringsstelsel van de mens. Ten eerste om de prevalentie van de resistentie te vergelijken en ten tweede om de mogelijke overgang van resistentiegenen van dier naar mens en vice versa te detecteren (van den Bogaard and Stobberingh, 2000). Een strenger criterium is het aanwezig zijn van een zoönotisch aspect.

Bij runderen echter is de prevalentie van enterokokken in de feces relatief laag. Enkel bij kalveren kunnen enterokokken frequent geïsoleerd worden (Devriese et al., 1992). Bij oudere koeien komt *Streptococcus bovis* veel frequenter voor dan enterokokken (Devriese et al., 1992). Volgens sommige onderzoekers zijn streptokokken weinig ontvankelijk voor resistentiegenen (van den Bogaard & Stobberingh, 2000), maar in de humane geneeskunde werd er onlangs een stijgende resistentie tegen penicilline waargenomen bij *Streptococcus pneumoniae* (McGowan, 2000). Ook in de diergeneeskunde werd de laatste 10 jaar een stijgende prevalentie van resistentie tegen macroliden, lincosamiden en tetracyclines waargenomen bij *Streptococcus suis* (Martel et al., 2001) en tegen macroliden bij *S. bovis* (Teng et al., 2001).

Het doel van deze studie was het opvolgen van het resistentieniveau van de indicatorbacteriën in het spijsverteringsstelsel op 3 verschillende bedrijfstypes, en aan te tonen

dat *S. bovis* meer geschikt is als Gram-positieve indicatorbacterie bij het rund dan *E. faecalis* and *E. faecium.*

3.2.2 Materiaal en methoden

3.2.2.1 Staalname

Gedurende twee jaar werden 10 melkveebedrijven, 10 zoogkoeienbedrijven en 5 vleeskalverenbedrijven regelmatig bemonsterd. Op de melkveebedrijven werden driemaal per jaar meststalen genomen, op de zoogkoeienbedrijven tweemaal per jaar en op de vleeskalverenbedrijven aan het begin en het einde van een productieronde. Op de melkvee –en zoogkoeienbedrijven werden stalen genomen van 50% van de dieren aanwezig op het bedrijf. Op de vleeskalverenbedrijven werden omwille van de grote omvang van deze bedrijven slechts stalen genomen van 20-25% van de dieren. De stalen werden binnen de 4 uur na staalname gekoeld getransporteerd naar het laboratrium. In totaal werden 2549, 1468 en 535 meststalen op de respectievelijke bedrijfstypes onderzocht. In elk staal werd gezocht naar de aanwezigheid van de traditionele indicatorkiemen, *E. coli* en enterokokken. Bijkomend werd *S. bovis* als nieuwe Gram-positieve indicatorbacterie geïntroduceerd en gevalideerd. Tevens werden de fecesstalen van de vleeskalveren onderzocht op de aanwezigheid van *Salmonella* species.

3.2.2.2 Bacteriële identificatie

De meststalen werden vooreerst geënt op 2 verschillende bodems: op een Slanetz and Bartley agar (Oxoid) voor de identificatie van de Gram-positieve indicatorkiemen en op een MacConkey agar (Oxoid) voor de identificatie van de Gram-negatieve indicatorkiemen. De Slanetz and Bartley agars werden gekookt tot het medium roze werd (ongeveer 3 min) en geïncubeerd gedurende 48h bij 37°C in 5% CO_2. De aanwezigheid van *Salmonella* werd onderzocht door middel van een rechtstreekse (Briljant Groen Agar, BGA) en selectieve aanrijkingsprocedure (Seleniet Cysteine broth) gevolgd door isolatie op BGA, telkens onder aërobe omstandigheden bij 37°C gedurende 24h.

De eerste selectie van *S. bovis* en enterokokken was gebaseerd op de specifieke kolonie morfologie (Tabel 1). De verdere identificatie van *S. bovis* was gebaseerd op een negatieve cultuur van het organisme op Brain Heart Infusion gesupplementeerd met 6,5% NaCl en een positieve amylase test. De amylase activiteit werd aangetoond door middel van een spot-on inoculatie op een Colombia agar die 1% starch bevat. De verdere identificatie van de enterokokken bestond uit een positieve cultuur op Brain Heart Infusion met 6,5% NaCl en het fermentatiepatroon van de suikers arabinose, mannitol, en raffinose.

Voor de identificatie van *E. coli* werden de mestmonsters geënt op een MacConkey agarplaat en geïncubeerd in aërobe omstandigheden bij 37°C gedurende 24h. De lactose-positieve (rode) kolonies werden geënt op een Kligler medium en op een indol medium, en geïncubeerd gedurende 24 h in aërobe omstandigheden (37°C). *E. coli* geeft een volledige geelverkleuring van het Kligler medium met gasvorming, en is indol positief.

3.2.2.3 Gevoeligheidsbepalingen

Er werden gevoeligheidsbepalingen verricht door middel van de disk diffusie test op 1923 *S. bovis* isolaten (1051 isolaten afkomstig van melkvee, 673 isolaten afkomstig van zoogkoeien en 199 isolaten van vleeskalveren), 4174 *E. coli* isolaten (2373 isolaten afkomstig van melkvee, 1295 isolaten afkomstig van zoogkoeien en 506 isolaten van vleeskalveren) en 76 enterokokken isolaten (37 isolaten afkomstig van melkvee, 21 isolaten afkomstig van zoogkoeien en 18 isolaten van vleeskalveren) De gevoeligheidsbepalingen werden verricht volgens de NCCLS-richtlijnen.

De volgende antibiotica werden getest voor de Gram-positieve indicatorkiemen: benzylpenicilline, tetracycline, gentamicine, enrofloxacine, erythromycine en lincomycine. Voor de gevoeligheidsbepalingen van *E. coli* werden volgende antibiotica getest: amoxicilline+clavulaanzuur, ampicilline, ceftiofur, tetracycline, trimethoprim/sulfonamide, neomycine, gentamicine, spectinomycine, streptomycine, nalidixinezuur, flumequine en enrofloxacine. De keuze van de geteste antibiotica was gebaseerd op de aanbevelingen van Caprioli et al. (2000) en op de wetenschap dat deze antibiotica vaak in de praktijk gebruikt worden. Verder werd er ook rekening gehouden met antibiotica die vaak gebruikt worden in de humane geneeskunde voor de behandeling van oa. streptokokkeninfecties. De platen werden bij 37°C geïncubeerd gedurende 18-24 uur. De platen met oxacilline werden geïncubeerd bij 30°C. De resultaten werden afgelezen met behulp van het automatische afleestoestel SIRSCAN® (i2a, Montpellier, France), en de interpretatie was gebaseerd op de fabrikant van de antibioticaschijfjes (Rosco, Taarstrup, Denemarken).

3.2.3 Resultaten

3.2.3.1 Prevalentie

Een overzicht van de fecale indicatorkiemen wordt weergegeven in Tabel 1. Geen enkele van de meststalen van de vleeskalveren was positief voor *Salmonella*. *Escherichia coli* werd op alle bedrijfstypes en in alle leeftijdscategorieën in zeer hoge aantallen geïsoleerd. Als Gram-negatieve indicatorbacterie voor het spijsverteringsstelsel stelt deze kiem wat betreft isolatie geen probleem.

Ook *S. bovis* werd op alle bedrijfstypes geïsoleerd (100%). De prevalentie van de traditionele Gram-positieve indicatorbacteriën (enterokokken) was beduidend lager. Tijdens periode I op de melkveebedrijven werden enterokokken slechts op 11 van de 20 bedrijven gevonden (55%). Voor *E. faecium* en *E. faecalis* bedroegen de prevalenties respectievelijk 15% (3/20) en 50% (10/20).

Op de vleeskalverenbedrijven was de prevalentie van de enterokokken aan het begin van de productieronde beduidend hoger dan op de melkvee- en zoogkoeienbedrijven en konden deze kiemen op alle bedrijven geïsoleerd worden, maar naarmate de dieren ouder werden, daalde de prevalentie beduidend en kon *E. faecalis* op nog slechts 1 bedrijf geisoleerd worden.

In Tabel 1 wordt een overzicht gegeven van de prevalentie van de fecale indicatorkiemen op de 3 bedrijfstypes en in Tabel 2 van de prevalentie bij de verschillende leeftijdscategorieën tijdens periode I op de melkveebedrijven, in het najaar 2002 op de zoogkoeienbedrijven en aan het begin van de productieronde op de vleeskalverenbedrijven.

Tabel 1. Prevalentie van de fecale indicatorkiemen op de 3 bedrijfstypes

Bedrijfstype	N	*Streptococcus bovis*	*Escherichia coli*	*Enterococci spp.*
**Melkveebedrijven**				
I*	453	25.8%	98.7%	1,99%
II	414	38.4%	99.5%	4,11%
III	464	31%	98.3%	1,72%
IV	390	43%	97.2%	1,03%
V	409	44%	95.8%	1,96%
VI	419	73%	92.1%	1,91%
**Zoogkoeienbedrijven**				
Najaar 2002	474	35.2%	95.9%	2,53%
Voorjaar 2003	398	43.5%	98.2%	2,01%
Najaar 2003	354	65%	93.2%	*4,80%*
Voorjaar 2004	242	56%	94.4%	*6,10%*
**Vleeskalverenbedrijven**				
Begin productieronde	289	34.9%	98.3%	12%
Einde productieronde	246	51%	95.9%	0,4%

*Periode I: april 2002-juli 2002, Periode II: augustus 2002-november 2002, Periode III: december 2002-maart 2003, Periode IV: april 2003-juli 2003, Periode V: augustus 2003-november 2003, Periode VI: december 2003-maart 2004

Tabel 2. Prevalentie van *S. bovis, E. faecalis* en *E. faecium* in de verschillende leeftijdscategorieën

Kiem	Bedrijfstype	Leeftijdscategorie			
		**< 6 maand**	**6-12 maand**	**> 12 maand**	**Total**
S. bovis	Melkvee	17/51 (33.3%)	10/64 (15.6%)	90/338 (26.6%)	117/453 (25.8%)
	Zoogkoeien	29/76 (38.2%)	17/94 (18.1%)	121/304 (39.8)	167/474 (35.2%)
	Vleeskalveren	107/289 (37%)	/	/	107/289 (37.0%)
	**Totaal**	**153/416 (36.8%)**	**27/158 (17.1%)**	**211/642 (32.9%)**	**391/1216 (32.2%)**
E. faecalis	Melkvee	1/51 (2.0%)	5/64 (7.8%)	2/338 (0.6%)	8/453 (1.8%)
	Zoogkoeien	*3/76 (3.9%)*	*1/94 (1.1%)*	*5/304 (1.6%)*	*9/474 (1.9%)*
	Vleeskalveren	17/289 (5.9%)	/	/	17/289 (5.9%)
	**Totaal**	**21/416 (5.0%)**	**6/158 (3.8%)**	**7/642 (1.1%)**	**34/1216 (2.8%)**
E. faecium	Melkvee	0/51 (0%)	1/64 (1.6%)	0/338 (0%)	1/453 (0.2%)
	Zoogkoeien	*0/76 (0%)*	*0/94 (0%)*	*3/304 (1.0%)*	*3/474 (0.6%)*
	Vleeskalveren	0/289 (0%)	/	/	0/289 (0%)
	**Totaal**	**0/416 (0%)**	**1/158 (0.6%)**	**3/642 (0.5%)**	**4/1216 (0.3%)**

3.2.3.2 Resistentieprofielen

Gram-positieve indicatorkiemen: *Streptococcus bovis*

Wanneer we de resistentieprofielen van de geïsoleerde *S. bovis* bekijken, valt er een groot verschil op tussen de verschillende bedrijfstypes (Tabel 3). Op de melkvee – en de zoogkoeienbedrijven kwamen er veel minder resistente stammen voor dan op vleeskalverenbedrijven. Op de zoogkoeienbedrijven observeerden we iets meer resistente stammen dan op de melkveebedrijven.

Op de vleeskalverenbedrijven was meer dan 90% van de *S. bovis* isolaten resistent tegenover tetracycline, erythromycine en lincomycine. Op de melkvee– en zoogkoeienbedrijven waren deze percentages veel lager. Penicillineresistentie kwam op geen enkel van de bedrijfstypes voor. Wanneer we de dynamiek van de resistentie vergelijken op de melkvee– en zoogkoeienbedrijven, zien we kleine veranderingen in de loop van de tijd, maar algemeen kan besloten worden dat het dynamisch karakter van de intestinale *S. bovis* isolaten relatief stabiel is. Ook op de vleeskalverenbedrijven zijn de veranderingen klein (Tabel 3).

Tabel 3. Gedetailleerd overzicht van het percentage resistente *Streptococcus bovis* isolaten uitgedrukt per antibioticum

Bedrijf	Periode	N	Peni	Tet	Gen	Enro	Ery	Linc
**MV***	Periode I	102	0	0,98	0	0	2,94	2,94
	Periode II	155	0	0,65	0	0	0,65	0
	Periode III	160	0	0,63	0	0	0	0
	Periode IV	160	0	0	0,63	0	0	0
	Periode V	176	0	0	0	0	0	0
	PeriodeVI	298	0	0	0	0	0	0
**ZK**	Najaar 2002	164	0	2,44	0	0	9,76	9,76
	Voorjaar 2003	161	0	2,48	2,48	0	3,73	3,73
	Najaar 2003	217	0	4,15	0	0,46	3,23	3,69
	Voorjaar 2004	160	0	0,76	0,76	2,29	2,29	2,29
**VK**	Begin	93	0	97,85	4,3	2,15	94,62	92,47
	Eind	106	0	99,06	0,94	0	98,11	96,23

*MV: melkvee, ZK: zoogkoeien; VK: vleeskalveren; Periode I: april 2002-juli 2002, Periode II: augustus 2002-november 2002, Periode III: december 2002-maart 2003, Periode IV: april 2003-juli 2003, Periode V: augustus 2003-november 2003, Periode VI: december 2003-maart 2004

Op de vleeskalverenbedrijven kwam multipele resistentie veelvuldig voor. Aan het begin van een productieronde waren gemiddeld 37,6% van de stammen resistent tegenover 3 of meer antibiotica. Deze multipele resistentie nam af naar het einde van de productieronde toe: nog 28,3% van de stammen was resistent tegenover 3 of meer antibiotica. De meest voorkomende combinatie op dit bedrijfstype was erythromycine/lincomycine/tetracycline (78,5% bij het begin van de ronde en 84,0% aan het einde van de ronde). Op de melkvee – en

zoogkoeienbedrijven kwam voor dezelfde drie moleculen resistentie het frequentst voor, en voornamelijk dan de combinatie erythromycine/lincomycine. Uit deze resultaten kwamen ook duidelijke bedrijfsverschillen naar voor wat betreft antibioticumgevoeligheid. Ook het aantal verschillende fenotypes dat binnen één bedrijf werd opgemeten, varieerde sterk.

Gram-positieve indicatorkiemen: *Enterococcus faecalis*

Zogoed als alle *E. faecalis* isolaten op alle bedrijfstypes waren resistent tegenover 1 of meerdere antibiotica. In Tabel 4 wordt een gedetailleerd overzicht gegeven van de resistentie uitgedrukt per antibioticum. Omwille van de kleine aantallen geïsoleerde enterokokkenisolaten, werden alle periodes van staalname per bedrijfstype samengenomen. Ook bij de enterokokken komt er op de vleeskalverenbedrijven beduidend meer resistentie voor. Alle isolaten op dit bedrijfstype waren resistent tegenover tetracycline, erythromycine en lincomycine.

Tabel 4. Gedetailleerd overzicht van het percentage resistente *Enterococcus faecalis* isolaten uitgedrukt per antibioticum

Bedrijf	N	Peni	Tet	Gen	Enro	Ery	Linc
**Melkvee**	37	2,70	54,05	5,41	0	32,43	97,30
**Zoogkoeien**	21	0	52,38	14,29	0	52,38	100
**Vleeskalveren**	18	11,11	100	5,56	16,67	100	100

Gram-negatieve indicatorkiemen: *Escherichia coli*

Wanneer we het algemeen resistentieprofiel van *E. coli* bekijken, is er ook hier een groot verschil tussen de verschillende bedrijfstypes waarneembaar. Op de vleeskalverenbedrijven waren bijna alle stammen, zowel bij het begin (97,83%) als aan het einde (98,28%) van de productieronde resistent tegenover 1 of meerdere antibiotica. Op de melkvee– en de zoogkoeienbedrijven kwamen er veel minder resistente stammen voor. Op de zoogkoeienbedrijven detecteerden we iets meer resistente stammen dan op de melkveebedrijven. In totaal waren op de zoogkoeienbedrijven 19,0% van de stammen resistent tegenover 1 of meerdere antibiotica terwijl dit percentage op de melkveebedrijven 8,43% bedroeg. Het aantal resistente stammen bleef relatief stabiel in de loop van de tijd (Tabel 5).

Op de vleeskalverenbedrijven werden de hoogste resistentiepercentages waargenomen. Er werd op dit bedrijfstype wel een afname in resistentie geobserveerd naarmate de dieren ouder werden. Op deze bedrijven werd er het meeste resistentie waargenomen tegenover tetracycline, ampicilline, streptomycine en trimethoprim sulfamide combinaties, en dit zowel bij het begin als aan het einde van de productieronde (Tabel 5). Ook op de melkvee- en de zoogkoeienbedrijven werd er het meest resistentie waargenomen tegenover tetracycline, ampicillin en streptomycine, waarbij de resistentiepercentages op de zoogkoeienbedrijven hoger waren dan op de melkveebedrijven.

Wanneer we de dynamiek van de resistentie vergelijken op de melkvee– en de zoogkoeienbedrijven, zien we kleine veranderingen in de loop van de tijd, maar algemeen kan besloten worden dat het dynamisch karakter van de intestinale *E. coli* isolaten qua resistentieprofiel relatief stabiel is (Tabel 5). Op de vleeskalverenbedrijven werd er voor alle antibiotica met uitzondering van tetracycline en ceftiofur, wel een duidelijke afname in

resistentie waargenomen. Op deze bedrijven kwam multipele resistentie echter veelvuldig voor. Aan het begin van een productieronde waren 68,9% van de stammen resistent tegenover 7 of meerdere antibiotica. De meest voorkomende combinatie was tetracycline /ampicilline /streptomycine /neomycine /gentamicine /timethoprim-sulfamide /nalidixinezuur /flumequine /enrofloxacine (24,8%). Deze multipele resistentie nam af naar het einde van de productieronde (Figuur 1): nog slechts 12,4% van de stammen was resistent tegenover 5 of meerdere antibiotica. De meest voorkomende combinatie was tetracycline/ ampicilline/ streptomycine/ trimethoprim-sulfamide (27,9%).

Tabel 5. Gedetailleerd overzicht van het percentage resistente *E. coli* isolaten uitgedrukt per antibioticum

Bedrijf	N	amp	amc	cef	tet	tri	neo	gen	spec	stre	nal	flu	enr
**Melkvee**													
Periode I	447	2,91	0	0	8,05	4,03	0,22	0	0,22	6,26	1,34	0	0
Periode II	396	2,02	0	0	3,54	0,25	1,01	0	0	4,04	0,25	0,25	0,25
Periode III	419	4,30	0	0	4,3	3,58	0,72	0,48	0	6,44	0,72	0,24	0,24
Periode IV	359	2,79	0,28	0	3,06	0,84	0,84	0	0	4,18	0,28	0,28	0,28
Periode V	382	5,24	0,79	0	3,14	3,66	0,26	0,52	0	8,38	0,26	0,26	0,26
Periode VI	370	3,24	0,54	0	4,32	1,89	1,08	0	0	5,68	1,35	0,27	0,27
**Zoogkoeien**													
Najaar ‘02	436	9,17	0,69	0	6,88	4,13	2,52	0,46	0,46	12,38	0,46	0,23	0,23
Voorjaar ‘03	346	12,14	0,87	0	17,05	5,49	4,62	2,31	0,87	16,47	6,07	2,31	1,73
Najaar ‘03	298	9,4	1,01	0,34	12,08	6,71	4,36	0,34	0	12,75	5,03	2,68	2,35
Voorjaar ‘04	215	17,21	0,93	0	15,81	6,05	5,12	2,33	0,47	22,79	7,91	5,12	4,19
**Vleeskalver.**													
Begin	27	93,1	1,09	0,36	94,9	92,0	81,1	44,2	3,62	86,9	76,0	66,6	38,0

Eind	6<br>23<br>0	2<br>79,5<br>7	0,43	0,43	3<br>95,2<br>2	3<br>63,0<br>4	6<br>16,0<br>9	3,04	0,43	6<br>65,2<br>2	9<br>10,8<br>7	7<br>3,04	4<br>1,3

amp: ampicilline; amc: amoxicilline + clavulaanzuur; cef: ceftiofur; tet: tetracycline; tri: trimethoprim/sulfamide; neo: neomycine; gen: gentamicine; spec: spectinomycine; stre: streptomycine; nal: nalidixinezuur; flu: flumequine; enr: enrofloxacine; Periode I: april 2002-juli 2002, Periode II: augustus 2002-november 2002, Periode III: december 2002-maart 2003, PeriodeIV: april 2003-juli 2003, PeriodeV: augustus 2003-november 2003, PeriodeVI: december 2003-maart 2004

Op de melkvee– en de zoogkoeienbedrijven kwam deze multipele resistentie veel minder frequent voor. Op deze bedrijfstypes was respectievelijk 32,0 en 26,0% van de isolaten resistent tegenover 1 antibioticum, en respectievelijk 41,5 en 24,8% tegenover 2 antibiotica. De meest voorkomende resistenties op de melkveebedrijven zijn tetracycline/streptomycine (16,5%), tetracycline (13,5%) en streptomycine (11,5%). Op de zoogkoeienbedrijven kwamen de volgende combinaties meest voor: tetracycline/ampicilline/streptomycine (15,9%), tetracycline/streptomycine (11,4%) en ampicilline/streptomycine (9,8%).

Uit deze resultaten kwamen ook duidelijke bedrijfsverschillen naar voor wat betreft antibioticumgevoeligheid. Ook het aantal verschillende fenotypes dat binnen een bedrijf werd opgemeten, varieerde sterk.

Figuur 1: Vergelijking van het voorkomen van multipele resistentie op vleeskalverenbedrijven in de loop van de tijd.

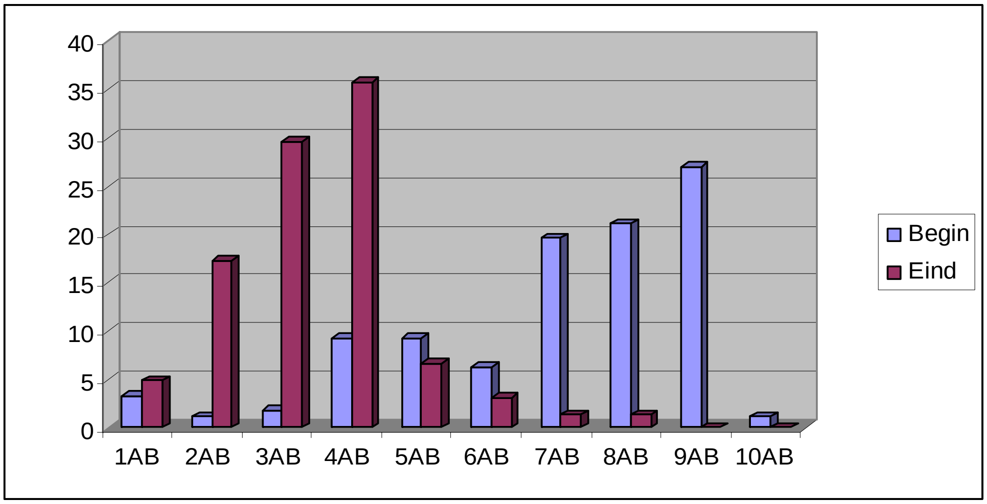

xAB: resistentie aanwezig voor x antibiotica. Begin: circa 3.5-5 weken na opzet productieronde, Eind: 23-25 weken van de productieronde

3.3 Microbiota van het ademhalingsstelsel

3.3.1 Inleiding

Naast de monitoring van de indicatorbacteriën uit het spijsverteringsstelsel, wilden we met deze studie tevens nagaan of er ter hoogte van de andere ecosystemen ook verschillen in antibioticumresistentie op te meten zijn, die in een later onderdeel zouden kunnen worden gerelateerd aan de uitgeoefende selectiedruk van het antibioticumgebruik. Een ander doel van deze uitbreiding naar andere ecosystemen was na te gaan of er overeenkomst was tussen het resistentieprofiel van de indicatorbacteriën ter hoogte van de verschillende orgaansystemen.

Voor het ademhalingsstelsel werd nagegaan of *Pasteurella multocida* en *Mannheimia haemolytica* geschikt zijn als 'indicatorbacteriën'. Van deze facultatief pathogene bacteriën is het geweten dat ze ook bij gezonde dieren in het bovenste deel van het ademhalingsstelsel kunnen voorkomen (Catry et al., 2004). Dit onderzoeksluik werd enkel uitgevoerd op zoogkoeienbedrijven en vleeskalverenbedrijven.

3.3.2 Materiaal en methoden

Op 10 zoogkoeien- en 5 vleeskalverenbedrijven werden regelmatig neusswabs genomen. Op de zoogkoeienbedrijven gebeurde dit om de 6 maanden, op de vleeskalverenbedrijven aan het begin en aan het einde van een productieronde. In het begin werden neusswabs genomen van alle dieren (op 6 van de 10 zoogkoeienbedrijven). Omwille van de lage nasale prevalentie van *Pasteurellaceae* bij volwassen dieren, werden later enkel nog dieren bemonsterd jonger dan 1 jaar, na ontsmetten van de neusspiegel. In totaal werden op de zoogkoeienbedrijven 682 stalen van gezonde dieren onderzocht op de aanwezigheid van *Pasteurellaceae* en op de vleeskalverenbedrijven 535 stalen.

De nasale swabs werden geënt op een bloedplaat gesupplementeerd met 5% schapenbloed en 15mg/L bacitracine (Catry et al., 2005) en geïncubeerd gedurende 24h in aërobe omstandigheden bij 37°C. Kolonies met een typisch uitzicht (rond, grijsachtig met een doormeter van 1 tot 3 mm) en cytochroomoxidase positief werden geselecteerd. *Mannheimia haemolytica* werd gekarakteriseerd door een nauwe hemolysezone, terwijl *Pasteurella multocida* een typische muizengeur produceert. Verdere fenotypische identificatie werd bevestigd door middel van een Kliglermedium, en indolproductie (afwezig voor *M. haemolytica*)

Er werden gevoeligheidsbepalingen verricht door middel van de disk diffusie test op 81 *P. multocida*- en op 38 *M. haemolytica* isolaten afkomstig van de zoogkoeienbedrijven, en op 164 *P. multocida*- en 47 *M. haemolytica* isolaten afkomstig van de vleeskalverenbedrijven. De gevoeligheidsbepalingen werden verricht volgens de NCCLS-richtlijnen zoals hoger beschreven. De volgende antibiotica werden getest: ampicilline, amoxicilline/clavulaanzuur, ceftiofur, tetracycline, trimethoprim/sulfonamide, neomycine, gentamicine, spectinomycine, nalidixinezuur, flumequine, enrofloxacine, en florfenicol. Ook hier werd een automatisch afleestoestel (SIRscan 2000) gebruikt voor de interpretatie van de diffusietesten.

3.3.3 Resultaten

De resultaten van de frequentie van isolatie van de *Pasteurellaceae* tijdens de verschillende staalnames worden weergegeven in Tabel 6. *P. multocida* werd aanzienlijk vaker geïsoleerd dan *M. haemolytica*. De nasale prevalentie van *Pasteurellaceae* bij volwassen dieren bleek zeer laag te zijn. In het najaar van 2002 kon slechts uit 3,5% van de neusswabs van volwassen dieren *P. multocida* geïsoleerd worden.

Tabel 6. Prevalentie van de indicatorkiemen in het ademhalingsstelsel op de twee verschillende bedrijfstypes

	N	*P. multocida*	*M. haemolytica*
**Zoogkoeien**			
Najaar 2002	369	16,8%	1,9%
Voorjaar 2003	110	49,1%	16,4%
Najaar 2003	101	27,7%	11,9%
Voorjaar 2004	102	38,2%	10,8%
**Vleeskalveren**			
Begin ronde	289	33,6%	3,8%
Einde ronde	246	17,8%	11,8%

Wanneer we het algemeen resistentieprofiel van de *P. multocida* isolaten bekijken, is er relatief weinig verschil enerzijds binnen de zoogkoeienbedrijven tussen de verschillende periodes en binnen de vleeskalverenbedrijven tussen het begin en het einde van een productieronde, en anderzijds tussen de 2 verschillende bedrijfstypes (Tabel 7). Er was echter wel een verschil tussen de twee bedrijfstypes wat betreft de resistentie per antibioticum. Op de zoogkoeienbedrijven werd hoofdzakelijk resistentie tegenover spectinomycine waargenomen. Er was 1 zoogkoeienbedrijf waar tijdens de 4 staalnamerondes telkens opnieuw resistente *Pasteurella multocida* isolaten geïsoleerd werden. De meest voorkomende resistentie tijdens de 4 periodes was dan ook spectinomycine. Op de vleeskalverenbedrijven werd er tegenover veel meer antibiotica resistentie waargenomen. Bij het begin van een productieronde werd er voornamelijk resistentie waargenomen tegenover gentamicine (43%), nalidixinezuur (43%), trimethoprim/sulfonamide (42%) en flumequine (37%). Op het einde van een productieronde werd het meest resistentie waargenomen tegenover tetracycline en trimethoprim/sulfonamide (Tabel 7). Over het algemeen waren de resistentiepercentages op het einde van een productieronde lager, behalve voor volgende antibiotica: tetracycline, ampicilline, neomycine, spectinomycine en enrofloxacine.

Drieëntachtig procent van de resistente *P. multocida* isolaten aan het begin van een productieronde waren resistent tegenover 4 of meer antibiotica. Deze multipele resistentie nam af naar het einde van de productieronde. Op dat moment waren 58% van de resistente isolaten resistent tegenover slechts 1 of 2 antibiotica. De meest voorkomende combinatie aan het begin van een productieronde was gentamicine/nalidixinezuur/flumequine/trimethoprim-sulfonamide (58%). Op het einde van de productieronde waren de meest voorkomende

combinaties: tetracycline (27%) en gentamicine/nalidixinezuur/flumequine/trimethoprim-sulfonamide (19%).

Tabel 7. Gedetailleerd overzicht van het percentage resistente *Pasteurella multocida* isolaten uitgedrukt per antibioticum

	N	amp	amc	cef	tet	tri	neo	gen	spec	nal	flu	enr
**Zoogkoeien**												
Najaar '02	**61**	0	0	0	0	18,0	0	0	23,0	0	0	0
Voorjaar '03	**53**	0	0	0	0	0	0	0	13,2	0	0	0
Najaar '03	**29**	0	0	0	0	0	0	0	34,5	0	0	0
Voorjaar '04	**38**	0	0	0	0	0	0	0	29,0	0	0	0
**Vleeskalveren**												
Begin	**86**	1,1	0	0	9,3	41,9	3,5	43,0	0	43,0	37,2	0
Einde	**78**	1,3	0	0	18,0	19,2	7,7	14,1	1,3	14,1	14,1	1,3

amp: ampicilline; amc: amoxicilline + clavulaanzuur; cef: ceftiofur; tet: tetracycline; tri: trimethoprim/sulfamide; neo: neomycine; gen: gentamicine; spec: spectinomycine; nal: nalidixinezuur; flu: flumequine; enr: enrofloxacine

Wat betreft de *M. haemolytica* isolaten was er een gelijkaardig verschil in algemeen resistentieprofiel tussen de 2 bedrijfstypes. Op de vleeskalverenbedrijven werden veel meer resistente isolaten geïsoleerd dan op de zoogkoeienbedrijven. Er was wel weinig verschil tussen de 4 periodes op de zoogkoeienbedrijven. Daar bleef het aantal resistente isolaten relatief constant. Ook tussen het begin en het einde van een productieronde op de vleeskalverenbedrijven werd er praktisch geen verschil waargenomen. In Tabel 8 wordt een overzicht gegeven van de resistentiepercentages per antibioticum.

Tabel 8. Gedetailleerd overzicht van het percentage resistente *Mannheimia haemolytica* isolaten uitgedrukt per antibioticum

	N	amp	amc	cef	tet	tri	neo	gen	spec	nal	flu	enr
**Zoogkoeien**												
**Najaar '02**	**4**	0	0	0	0	25,0	0	0	0	25,0	25,0	0
**Voorjaar '03**	**14**	0	0	0	0	0	0	0	0	0	0	0
**Najaar '03**	**9**	0	0	0	0	0	0	0	11,1	0	0	0
**Voorjaar '04**	**11**	0	0	0	0	0	9,1	0	9,1	0	0	0
**Vleeskalveren**												
**Begin**	**12**	50,0	0	0	75,0	58,3	16,7	58,3	16,7	50,0	50,0	33,3
**Einde**	**36**	75,0	0	0	50,0	25,0	5,6	13,9	0	22,2	19,4	16,7

amp: ampicilline; amc: amoxicilline + clavulaanzuur; cef: ceftiofur; tet: tetracycline; tri: trimethoprim/sulfamide; neo: neomycine; gen: gentamicine; spec: spectinomycine; nal: nalidixinezuur; flu: flumequine; enr: enrofloxacine

Op de zoogkoeienbedrijven komt er weinig resistentie voor. In totaal zijn er twee isolaten resistent ten opzichte van spectinomycine, 1 ten opzichte van neomycine en 1 ten opzichte van trimethoprim-sulfonamide, nalidixinezuur en flumequine samen. Op de vleeskalverenbedrijven zijn de resistentiepercentages beduidend hoger. Aan het begin van de productieronde werd er voornamelijk resistentie waargenomen ten opzichte van volgende antibiotica: tetracycline (75%), trimethoprim sulfonamide combinaties (58%) en gentamicine (58%). Op het einde van de ronde is de resistentie ten opzichte van alle antibiotica gedaald, behalve voor ampicilline. De hoogste resistentiepercentages werden dan ook waargenomen voor ampicilline (75%) en tetracycline (50%). Tachtig procent van de resistente *M. haemolytica* isolaten aan het begin van een productieronde waren resistent tegenover 4 of meer antibiotica. Ook hier nam deze multipele resistentie af aan het einde van de productieronde. Op dat moment was 74% van de resistente isolaten resistent tegenover slechts 1 of 2 antibiotica.

De meest voorkomende combinatie aan het begin van de productieronde was ampicilline/tetracycline/gentamicine/nalidixinezuur/flumequine/enrofloxacine/trimethoprim-sulfonamiden (30%). Op het einde van de productieronde waren de meest voorkomende combinaties: ampicilline/tetracycline (35%) en ampicilline (32%).

3.4 Discussie

3.1.1. *Spijsverteringsstelsel*

We kunnen besluiten dat er zowel voor *S. bovis* als voor *E. coli* veel verschil bestaat tussen de verschillende bedrijfstypes en de verschillende bedrijven wat betreft de prevalentie van resistentie. Op de vleeskalverenbedrijven liggen de resistentiepercentages veel hoger dan op de andere bedrijfstypes en ook multipele resistentie komt op dit bedrijfstype veel meer voor. Op deze bedrijven zien we voor *S. bovis* een daling van de multipele resistentie naarmate de dieren ouder worden, ondanks een stijging van de monoresistentie. Dit werd waargenomen voor alle geteste antibiotica met uitzondering van tetracycline. Voor de *E. coli* isolaten werd zowel een daling van de multipele resistentie als van de monoresistentie waargenomen, met uitzondering voor tetracycline. Op de melkvee- en de zoogkoeienbedrijven zien we weinig of geen verandering in de tijd in het resistentieprofiel van de fecale indicatorkiemen.

Uit de resultaten van de meststaalnames kunnen we besluiten dat voor de Gram-positieve bacteriën van het ecosysteem van het spijsverteringsstelsel, *Streptococcus bovis* frequenter werd geïsoleerd als indicatorbacterie dan *Enterococcus faecium* en *Enterococcus faecalis*. In tegenstelling tot *S. bovis* die op alle bedrijven, in alle bedrijfstypes en alle leeftijdcategorieën voorkwam, konden enterokokken niet op alle bedrijven geïsoleerd worden. Dit komt overeen met de bevindingen in eerdere studies. Ook in een studie van Devriese et al. (1992) kwam *S. bovis* veelvuldig voor in de mest van jongvee en volwassen melkkoeien. Bij jonge kalveren werd in die studie *E. faecalis* het meest geïsoleerd.

Door het vergelijken van eerdere studies (Cain et al., 1995; McGowan, 2000; Teng et al., 2001) met de resultaten van de resistentiebepalingen van de enterokokken en streptokokken door middel van de gebruikte diffusietesten, werd het evenwel duidelijk dat de interpretatie van deze testen onderhevig zou kunnen geweest zijn aan fouten (onzekerheden). Bovendien is het niet duidelijk of deze onzekerheden veroorzaakt zijn door een foutieve identificatie (verschillende intrinsieke gevoeligheid tussen bacteriële species), dan wel door de gebruikte techniek voor de gevoeligheidsbepalingen (agar diffusietechniek). Vermits deze problemen een onderzoek naar het verband tussen het antibioticumgebruik en het voorkomen

van antibioticumresistentie zullen hypothekeren, evenals ook een onderzoek naar de relatie tussen indicatorbacteriën en facultatief pathogene kiemen, is een verder onderzoek naar de nauwkeurigheid van de resistentiemonitoring gewenst.

3.1.2. Ademhalingsstelsel

P. multocida en *M. haemolytica* komen veelvuldig voor in de ademhalingstractus van gezonde kalveren. Bij volwasssen dieren echter is de prevalentie van beide kiemen zeer laag. We kunnen besluiten dat er zowel voor *P. multocida* als voor *M. haemolytica* veel verschil bestaat tussen de verschillende bedrijfstypes en de verschillende bedrijven wat betreft de prevalentie van resistentie. Op de vleeskalverenbedrijven liggen de resistentiepercentages voor de meeste antibiotica veel hoger dan op de zoogkoeienbedrijven en ook multipele resistentie komt op dit bedrijfstype veel meer voor. Op deze bedrijven zien we voor beide kiemen een daling van de multipele resistentie naarmate de dieren ouder worden en een daling van de monoresistentie met uitzondering van tetracycline voor *P. multocida* en ampicilline voor *M. haemolytica.*

Met betrekking tot de identificatie van de *Pasteurellaceae* dient er evenwel rekening mee te worden gehouden dat met de gebruikte fenotypering geen onderscheid mogelijk is tussen *M. haemolytica* en aanverwante species binnen het genus *Mannheimia*. Eerder onderzoek toonde bijvoorbeeld aan dat fenotypisch geïdentificeerde *M. haemolytica* stammen uit de neusholte van gezonde kalveren, niet behoorden tot de species *M. haemolytica senso stricto* (Catry et al., 2005). Moleculaire identificatie tot op het speciesniveau is daarom noodzakelijk. Ook de nauwkeurigheid van de gebruikte diskdiffusietesten is ongekend, hetgeen van belang is voor zowel de practicus, als voor de analytische waarde van dergelijke gegevens in epidemiologische studies.

Omdat elke bacteriële species een kenmerkende intrinsieke gevoeligheid bezit ten opzichte van een bepaald antibioticum, hangt de nauwkeurigheid van resistentieopmetingen enerzijds af van een correcte identificatie van de betrokken bacterie, en anderzijds van de gebruikte gevoeligheidstest. In de loop van de eerste resistentiebepalingen (zie hoger) was gebleken dat voor bepaalde bacterie-antibioticum combinaties geen betrouwbare opmetingen van het resistentiepatroon mogelijk waren wanneer enkel klassieke antibiogrammen (kwalitatieve diffusietesten) aangelegd werden. Ook was het met de toen aangewende fenotypische technieken niet mogelijk om bepaalde kiemen met zekerheid tot op species niveau te identificeren. Dit was vooral het geval voor de *Pasteurellaceae* en bepaalde enterokokken en streptokokken. Dit verhinderde om verder verbanden tussen het antibioticumgebruik en het opgemeten resistentieprofiel enerzijds, en tussen de indicatorbacteriën en pathogene bacteriën anderzijds, te verduidelijken.

Deze studie heeft dan ook als doelstelling om een aantal bijkomende testen uit te voeren, met name kwantitatieve dilutietesten en genotypische speciesidentificatie. De bekomen testkarakteristieken, kunnen vervolgens in een tweede onafhankelijke dataset worden gebruikt om veldgegevens te corrigeren. M.a.w. het laat toe om disk diffusieresultaten te herberekenen met inbegrip van de nauwkeurigheid van de gebruikte testen. Deze zullen het mogelijk maken de globale monitoringgegevens nauwkeuriger te associëren met het antibioticumgebruik.

4.1. Bacteriële identificatie

4.1.1. Materiaal en methoden

Een selectie van 258 indicatorbacteriën geïsoleerd uit het spijsverteringsstelsel en fenotypisch geklasseerd als *Streptococcus* en *Enterococcus* spp. werd onderworpen aan tRNA-intergenic spacer PCR. Daarnaast werden 177 *Pasteurellaceae* geïsoleerd uit het ademhalingsstelsel van gezonde dieren onderworpen aan dezelfde techniek (Baele *et al.*, 2000; 2001; Catry *et al.*, 2004). Deze techniek is gebaseerd op het feit dat de intergenische gebieden tussen aangrenzende tRNA-genen op gebied van lengte verschillen tussen verschillende bacteriën. De verschillende tRNA-genen zijn namelijk behouden binnen de verschillende soorten van een bepaald genus, maar de afstand ertussen varieert en is dus karakteristiek voor een welbepaalde bacteriesoort. De accuraatheid van de identificatie van de *Pasteurellaceae* en enterokokken/*Streptococcus bovis* werd onderzocht door het vergelijken van de toegepaste fenotypering met genotypering.

Vermits *E. coli* fenotypisch een voldoende correct te identificeren organisme is, werd deze genotypische techniek voor deze kiemspecies niet toegepast.

4.1.2. Resultaten

De resultaten voor de verschillende kiemsoorten worden weergegeven in Tabel 1. Hieruit blijkt dat de identificatie van *P. multocida* en *M. haemolytica* sensu lato goed is, terwijl dit voor de enterokokken en streptokokken veel minder gunstig blijkt.

Tabel 1. Overeenkomst tussen fenotypering en genotypering voor *Pasteurellaceae*, enterokokken en streptokokken.

Fenotypering	N isolaten	Genotypering (tDNA PCR)	% Overeenkomst
P. multocida	146	141	96.6 %
M. haemolytica	31	29 (sensu lato)[a]	93.5 %
		14 (sensu stricto)	45.2 %
E. faecalis	26	20	76.9%
S. bovis	232	147 (sensu lato)[b]	63.4%
		104 (sensu stricto)	43.1%

[a] *M. glucosida, M. varigena* en andere hemolytische *Mannheimia* spp.
[b] *S. gallolyticus* (n= 32), *S. alactolyticus, S. equinus, S. infantarius*

Verder moleculair onderzoek aan het laboratorium Microbiologie van de faculteit Wetenschappen, Universiteit Gent, heeft aangetoond dat voor een selectie van 25 stammen die als *S. bovis* sensu lato werden getypeerd met behulp van tDNA-PCR, hiervan 80% (N=20) als *S. bovis* sensu stricto werd bevonden. De hiertoe aangewende techniek betrof het sequeneren van twee huishoudgenen (*rpoA* en pheS) (P. Vandamme, persoonlijke communicatie, 2007). De overige 5 stammen werden met behulp van deze techniek geklasseerd als *S. infantarius* subspecies *infantarius* (n=1) en *S. gallolyticus* subspecies *gallolyticus* (n=4).

4.2. Gevoeligheidsbepalingen

Simultaan met de moleculaire identificatie met behulp van tRNA-intergenic spacer PCR werden enerzijds de fecale streptokokken/enterokokken en anderzijds de respiratoire *Pasteurellaceae* onderworpen aan gevoeligheidstesten voor eenzelfde gamma antibiotica als gebruikt tijdens de diffusietesten beschreven in hoofdstuk 3. In tegenstelling tot de agar diffusietesten waarbij inhibitiezones worden verkregen die gecorreleerd kunnen worden met gevoeligheid of resistentie, worden met dilutietesten minimale inhibitorische concentraties verkregen voor elk getest antibioticum. De minimale inhibitorische concentratie (MIC) van een individuele stam wordt gedefinieerd als de laagste antibioticumconcentratie waarbij op het oog geen bacteriegroei waar te nemen is.

De interpretatie van de MIC bepalingen gebeurde volgens het microbiologisch criterium (mono/bimodaliteit/*tailing*) en/of CLSI-richtlijnen. De diffusietesten werden geïnterpreteerd volgens de fabrikant van de antibiotica-schijfjes. Een overzicht van de interpretatiecriteria voor de *Pasteurellaceae* en enterokokken/streptokken is terug te vinden in respectievelijk Addendum 1 en 2..

De resultaten van de vergelijking tussen de agar dilutie en disk diffusietesten werden vooreerst als een ‘error rate analysis’ geëvalueerd (Jorgensen & Ferraro, 1998). In Tabel 2 (*Pasteurellaceae*) en Tabel 3 (enterokokken/streptokokken) kan het percentage foutief aangeduide resistente kiemen worden afgeleid als het percentage *Major Errors* (ME; disk diffusie resistent en agar dilutie gevoelig). Evenzo kan het percentage foutief aangeduide gevoelige kiemen worden afgeleid uit het percentage *Very Major Errors* (VME; disk diffusie gevoelig en agar dilutie resistent). Een *Minor error* (mE) wordt gedefinieerd als 1 van de twee testen ‘intermediair gevoelig’ aangeeft, terwijl de andere test in gevoelig of resistent resulteert. Traditioneel aanvaardbare grenswaarden voor de VME, ME en totale fouten (som

van VME, ME en mE) zijn respectievelijk ≤ 1,5%, ≤ 3%; en ≤10%. Vetgedrukte waarden in de Tabellen 2 en 3 duiden op het overschrijden van deze richtgetallen.

Tabel 2. Vergelijking tussen de agar dilutie methode en disk diffusie methode voor de *Pasteurellaceae*.

**Product**	**Species** [b]	**Testresultaat en karakteristiek** [a]					
	**(N. stammen)**	**Agar Dilutie resistent**	**Disk diffusie resistent**	**Essential agreement**	**Very major error**	**Major error**	**Minor Error**
**Ampicilline**	*P. multocida* (135)	5,2%	12,6%	92,6 %	0,0 %	3,0 %	4,4 %
	M. haemolytica s.l. (34)	38,2%	32,4%	94,1 %	**5,9 %**	0,0 %	0,0 %
**Ceftiofur**	*P. multocida* (135)	0%	0%	100,0 %	0,0 %	0,0 %	0,0 %
	M. haemolytica s.l. (34)	0%	0%	100,0 %	0,0 %	0,0 %	0,0 %
**Oxytetracycline**	*P. multocida* (135)	20,7%	18,5%	91,9 %	**3,0 %**	0,0 %	5,2%
	M. haemolytica s.l. (34)	35,5%	26,5%	**85,3 %**	**8,8 %**	0,0 %	5,9 %
**Gentamicine**	*P. multocida* (108)	11,1%	9,5%	96,3 %	0,0 %	0,9 %	2,8 %
	M. haemolytica s.l. (31)	9,7%	16,1%	**87,1 %**	**3,2 %**	**3,2 %**	6,5 %
**Florfenicol**	*P. multocida* (135)	0%	0%	100,0 %	0,0 %	0,0 %	0,0 %
	M. haemolytica s.l. (31)	0%	0%	100,0 %	0,0 %	0,0 %	0,0 %
**Enrofloxacine**	*P. multocida* (135)	5,9%	3,7%	97,0 %	0,0 %	0,0 %	3,0 %
	M. haemolytica s.l. (34)	14,7%	14,7%	100,0 %	0,0 %	0,0 %	0,0 %
**TMP/S**[c]	*P. multocida* (135)	33,3%	23,7%	**83,7 %**	**3,7 %**	0,0 %	**12,6 %**
	M. haemolytica s.l. (27)	22,2%	25,9%	**81,5 %**	**7,4 %**	**3,7 %**	7,4 %
	Total (162)	31,5%	24,1%	**83,3 %**	**4,3 %**	0,6 %	**11,7 %**

[a] *Essential agreement*: resultaten van beide technieken zijn identiek; *very major error*: resistente stam volgens de agar dilutie methode die foutief als gevoelig werd bestempeld in de disk diffusie; *major error*: gevoelige stam volgens agar dilutie die foutief als resistent werd aangeduid in de disk diffusie; *minor error*: intermediair gevoelig volgens slechts één van de twee testmethodes. Vetgedrukte resultaten wijken af van de vooropgestelde grenswaarden. [b]s.l.: sensu lato. [c]TMP/S: trimethoprim-sulfonamiden.

Tabel 3. Vergelijking tussen de agar dilutie methode en disk diffusie methode voor de streptokokken en enterokokken.

Product	Species [b] (N. stammen)	Agar Dilutie resistent	Disk diffusie resistent	Essential agreement	Very major error	Major error	Minor Error
		Test result and characteristics [a]					
**Ampi/Penicilline**	*S. bovis* s.l. (115)	0,0%	0,0%	95,7 %	0,0 %	0,0 %	4,3 %
	E. faecalis (15)	0,0%	6,7%	93,3 %	0,0 %	**6,7 %**	0,0 %
**Oxytetracycline**	*S. bovis* s.l. (115)	34,8%	26,1%	91,3 %	**5,2 %**	0,0 %	3,5 %
	E. faecalis (15)	80,0%	73,3%	93,3 %	**6,7 %**	0,0 %	0,0 %
**Lincomycine**	*S. bovis* s.l. (115)	31,3%	29,6%	93,9 %	**1,7 %**	0,0 %	4,3%
**Erythromycine**	*S. bovis* s.l. (115)	29,6%	29,6%	93,0 %	0,9 %	1,7 %	4,3 %
	E. faecalis (15)	86,7%	73,3%	**80,0 %**	**6,72 %**	0,0 %	**13,3 %**

[a] *Essential agreement*: resultaten van beide technieken zijn identiek; *very major error*: resistente stam volgens de agar dilutie methode die foutief als gevoelig werd bestempeld in de disk diffusie; *major error*: gevoelige stam volgens agar dilutie die foutief als resistent werd aangeduid in de disk diffusie; *minor error*: intermediair gevoelig volgens slechts één van de twee testmethodes. Vetgedrukte resultaten wijken af van de vooropgestelde grenswaarden. [b]s.l.: sensu lato.

Naast deze traditionele analyse werden tevens klassieke epidemiologische testkarakteristieken (sensitiviteit en specificiteit, positief en negatief voorspellende waarde) berekend. Voor de berekeningen van de sensitiviteit en specificiteit werd de agar dilutietest als gouden standaard beschouwd en werden de intermediaire resultaten als resistent beschouwd. De sensitiviteit, specificiteit, resistent (positief) en gevoelig (negatief) voorspellende waarden voor de gevoeligheidstesten van respectievelijk de *Pasteurellaceae* en de enterokokken/streptokokken zijn weergegeven in Tabellen 4 en 5. De sensitiviteit werd berekend als het aantal resistente (+intermediaire) stammen volgens disk diffusie gedeeld door het aantal resistente (+intermediaire) stammen volgens agar dilutie. De specificiteit werd berekend als het aantal gevoelige stammen volgens disk diffusie gedeeld door het aantal gevoelige stammen volgens agar dilutie. De resistent (positief) voorspellende waarde (RPV) werd berekend als de probabiliteit dat een stam werkelijk resistent is indien de disk diffusie methode de stam als resistent beschouwt. De gevoelig (negatief) voorspellende waarde (SPV) werd berekend als de probabiliteit dat en stam werkelijk gevoelig is indien door de disk diffusie als gevoelig bestempeld.

Er dient te worden benadrukt dat de traditionele *error rate analysis* (Tabellen 2 en 3) voor sommige antibiotica een vrij verschillende beoordeling van de disk diffusie geeft in vergelijking met de sensitiviteit- en specificiteit-bepalingen (Tabellen 4 en 5). Dit is grotendeels het gevolg van het feit dat de traditionele analyse geen informatie verschaft over de kwaliteit van de test voor een bepaald antibioticum indien resistentie afwezig is (100% agreement) terwijl bij de berekening van de sensitiviteit en specificiteit in dat geval correct wordt aangegeven dat het niet mogelijk is om de sensitiviteit van de test te evalueren. Net daarom werd voor de volgende analyses de sensitiviteit en specificiteit bepaald.

Tabel 4.Testkarakteristieken van de disk diffusietest voor *Pasteurellaceae* in vergelijking met de agar dilutie techniek (gouden standaard).

		Testkarakteristieken [a]					
**Product**	**Species (N. stammen)**	**Sensit.**	**(95% CI)**	**Specif.**	**(95% CI)**	**RPV**	**SPV**
**Ampicilline**	*P. multocida* (135)	100 %	100-100 %	92.2 %	87.8-96.7 %	**41.2 %**	100 %
	M. haemolytica s.l. (34)	84.6 %	72.5-96.7 %	100 %	100-100 %	100 %	91.3 %
**Ceftiofur**	*P. multocida* (135)	NC[b]	NC	100 %	100-100 %	NC	100 %
	M. haemolytica s.l. (34)	NC	NC	100 %	100-100 %	NC	100 %
**Oxytetracycline**	*P. multocida* (135)	85.7 %	79.8-91.6 %	99.1 %	97.4-100 %	96.0 %	96.4 %
	M. haemolytica s.l. (34)	75.0 %	60.4-89.6 %	100 %	96.3-100 %	100 %	88.0 %
**Gentamicine**	*P. multocida* (108)	75.0 %	66.8-83.2 %	99.0 %	97.0-100 %	90.0 %	96.9 %
	M. haemolytica s.l. (31)	66.7 %	50.1-83.3 %	89.3 %	78.4-100 %	**40.0 %**	96.2 %
**Florfenicol**	*P. multocida* (135)	NC	NC	100 %	100-100 %	NC	100 %
	M. haemolytica s.l. (31)	NC	NC	100 %	100-100 %	NC	100 %
**Enrofloxacine**	*P. multocida* (135)	62.5 %	54.3-70.7 %	100 %	100-100 %	100 %	97.7 %
	M. haemolytica s.l. (34)	100 %	100-100 %	100 %	100-100 %	100 %	100 %
**TMP/S[c]**	*P. multocida* (135)	71.1 %	63.5-78.8 %	100 %	100-100 %	100 %	87.4 %
	M. haemolytica s.l. (27)	66.7 %	48.9-84.4 %	85.7 %	72.5-98.9 %	**57.1 %**	90.0 %

[a] Sensitiviteit (Sensit.): aantal resistente (+intermediaire) stammen volgens disk diffusie/aantal resistente (+intermediaire) stammen volgens agar dilutie, specificiteit (Specif.): aantal gevoelige stammen volgens disk diffusie/aantal gevoelige stammen volgens agar dilutie, RPV: resistent (positief) voorspellende waarde; probabiliteit dat een stam werkelijk resistant is indien de disk diffusie methode de stam als resistant beschouwt, SPV: gevoelig (negatief) voorspellende waarde; probabiliteit dat en stam werkelijk gevoelig is indien door de disk diffusie als gevoelig bestempeld. CI 95%: 95% confidentie interval. [b] NC: Niet berekend omdat geen isolaten resistentie vertoonden.. [c]TMP/S: trimethoprim-sulfonamiden. Vetgedrukte waarden zijn voorspellende waarden kleinder dan 75%.

Tabel 5. Testkarakteristieken van de disk diffusietest voor streptokokken/enterokokken in vergelijking met de agar dilutie techniek (gouden standaard).

		Test characteristic [a]					
**Antimicrobial agent**	**Species (No. of strains)**	**Sensit.**	**(95% CI)**	**Specif.**	**(95% CI)**	**RPV**	**SPV**
**Ampi/Penicillin**	*S. bovis* s.l. (115)	0.0 %	0.0-0.0 %	98.2 %	95.8-100 %	**0.0 %**	97.3 %
	E. faecalis (15)	NC[b]	NC	93.3%	80.7-100 %	**0.0 %**	100.0
**Oxytetracycline**	*S. bovis* s.l. (115)	85.0 %	78.5-91.5	100 %	100-100 %	100 %	75.0 %
	E. faecalis (15)	91.7 %	77.7-100.0 %	100 %	100-100 %	NC	100 %
**Lincomycin**	*S. bovis* s.l. (115)	82.9 %	76.0-89.8 %	100 %	100-100 %	100 %	91.4 %
**Erythromycin**	*S. bovis* s.l. (115)	97.1 %	94.0-100 %	92.6 %	87.8-97.4%	84.6 %	98.7 %
	E. faecalis (15)	86.7 %	69.5-100	NC	NC	100 %	**0.0 %**

[a] Sensitiviteit (Sensit.): aantal resistente (+intermediaire) stammen volgens disk diffusie/aantal resistente (+intermediaire) stammen volgens agar dilutie, specificiteit (Specif.): aantal gevoelige stammen volgens disk diffusie/aantal gevoelige stammen volgens agar dilutie, RPV: resistent (positief) voorspellende waarde; probabiliteit dat een stam werkelijk resistent is indien de disk diffusie methode de stam als resistent beschouwt, SPV: gevoelig (negatief) voorspellende waarde; probabiliteit dat en stam werkelijk gevoelig is indien door de disk diffusie als gevoelig bestempeld. CI 95%: 95% confidentie interval. [b] NC: Niet berekend omdat geen isolaten resistentie vertoonden.. [c]TMP/S: trimethoprim-sulfonamiden. Vetgedrukte waarden zijn voorspellende waarden kleinder dan 75%.

Op basis van de sensitiviteit- en specificiteit bepalingen worden de gevonden kritische kiem-antibiotium combinaties samengevat in Tabel 6. Deze tabel somt op welke resultaten (resistent en/of gevoelig) voor welke kiem-antibioticum combinaties niet betrouwbaar zijn. Hierbij dient te worden opgemerkt dat deze resultaten met voorzichtigheid dienen te worden geïnterpreteerd voor *E. faecalis* wegens het beperkt aantal geteste stammen (15). Bovendien werden in de tabellen van de streptokokken/enterokokken (Tabellen 3 & 5) de resultaten voor enrofloxacine, gentamicine en lincomycine voor *E. faecalis* niet opgenomen. De MIC bepalingen illustreerden immers een monomodale verdeling wat aantoont dat het gaat om intrinsieke gevoeligheid, en er bijgevolg geen sprake is van verworven resistentie. Nochtans gaven de afkapwaarden (breakpoints) volgens het internationaal referentiecentrum voor gevoeligheidstesten (NCCLS) aan, dat verschillende van deze stammen als resistent of intermediair resistent geklasseerd kunnen worden (afkapwaarden: zie bijlage).

Tabel 6. Overzicht van diffusietest-uitslagen die onderhevig zijn aan een duidelijke foutmarge.

**Gram-negatief**	**Resultaat**	**Gram-positief**	**Resultaat**
P. multocida en ampicilline	Resistent	*S. bovis* en penicilline	Resistent
M. haemolytica s.l. en gentamicine	Resistent	*E. faecalis* en penicilline	Resistent
M. haemolytica s.l. en TMP/S[a]	Resistent	*E. faecalis* en erythromycine	Gevoelig

[a] TMP/S: trimethoprim-sulfonamides

4.3. Correcties van gevoeligheidsbepalingen in functie van testkarakteristieken

In tegenstelling tot de positief (resistent) en negatief (gevoelig) voorspellende waarden, zijn de sensitiviteit en specificiteit onafhankelijk van de prevalentie. Dit laat toe om de specificiteit en sensitiviteit in een 'onafhankelijke' dataset te gaan gebruiken en om op basis van de schijnbare prevalentie (test resultaat, *Pa =apparent prevalence*) de ware prevalentie (true prevalence, *Pt*) te gaan schatten. M.a.w. de prevalentie van diskdiffusie-uitslagen kan worden bijgestuurd door de eventuele onnauwkeurigheid van deze test in rekening te brengen. Dit werd gedaan door middel van de Rogan-Gladen schatter (1), die een schijnbare prevalentie herberekent naar een ware prevalentie. De Rogan-Gladen schatter wordt links (0.0) en rechts (1.0) gelimiteerd, respectievelijk voor waarden kleiner dan 0 en groter dan 1 (Rogan & Gladen, 1978).

$$P_t = \frac{P_a + Sp - 1}{Se + Sp - 1} \qquad (1)$$

Deze berekeningswijze werd voltrokken voor een tweede dataset *Pasteurellaceae*, waarbij de vergelijking werd gemaakt tussen isolaten afkomstig van de extensief gehouden kalveren (vleesveebedrijven) en de isolaten van de intensief gehouden vleeskalveren (Tabel 7).

Tabel 7. Vergelijking van de schijnbare prevalentie (*apparent prevalence:* P_a) van resistentie op basis van disk diffusieresultaten en de berekende ware prevalentie (*true prevalence:* P_t) van resistentie op twee verschillende bedrijfstypes.

Bedrijfstype	Prevalentie resistent	Amp*	Cef	Tet	Gen	Flor	Enro	Tmp-S
**Extensieve teelt**								
P. multocida (155)	P_a	3,2%	0,0%	0,0%	0,0%	0,0%	0,0%	4,5%
	P_t	0,0%	-[£]	0,0%	0,0%	-[£]	0,0%	6,4%
M. haemolytica sensu lato (41)	P_a	7,3%	0,0%	0,0%	4,9%	0,0%	0,0%	0,0%
	P_t	8,6%	-[£]	0,0%	0,0%	-[£]	0,0%	0,0%
**Vleeskalveren**								
P. multocida (75)	P_a	9,3%	0,0%	38,7%	42,7%	0,0%	36,0%	44,0%
	P_t	1,6%	-[£]	44,5%	56,3%	-[£]	57,6%	61,9%
M. haemolytica sensu lato (24)	P_a	100,0%	0,0%	91,7%	20,8%	0,0%	12,5%	25,0%
	P_t	100,0%	-[£]	100,0%	18,1%	-[£]	12,5%	20,5%

[*] Amp: ampicilline; Cef: ceftiofur; Tet: oxytetracycline; Gen: gentamicine; Flor: florfenicol; Enro: enrofloxacine; Tmp/S: trimethoprim-sulfonamiden.
[£] niet berekenbaar omdat resistentie afwezig is.

4.4. Discussie

Deze studie heeft aangetoond dat monitoring van antibioticumresistentie op rundveebedrijven onderhevig kan zijn aan een grote foutmarge wanneer Gram-positieve indicatorbacteriën van de genera *Enterococcus* en *Streptococcus* louter door fenotypische speciesidentificatie en disk diffusie gevoeligheidsbepalingen worden ingesloten. Zowel de identificatie als de gevoeligheidsbepalingen van deze organismen, met de nadruk op *Streptococcus bovis*, zijn aan een te grote fout onderhevig om met de gebruikte methodologie betrouwbaar te worden ingezet in monitoringsprogramma's. Recent onderzoek heeft dit trouwens bevestigd voor gevoeligheidsbepalingen voor fluoroquinolones en verschillende viridans streptokokken (Rodriguez-Avial *et al.*, 2007). Vermits Gram-positieve bacteriën echter in de humane geneeskunde een toenemend belang kennen met betrekking tot multi-resistente pathologieën, verdient deze groep kiemen verdere onderzoeksmiddelen, met inbegrip van studies rond een mogelijke overdracht van resistentie naar de mens (o.a. *erm* genen).

Met betrekking tot de enterokokken/streptokokken en *Pasteurellaceae*, werd voor de eerste maal de resistent en gevoelig voorspellende waarde bepaald van disk diffusie gevoeligheidsbepalingen. Deze innoverende analyse, bood trouwens de mogelijkheid om tegelijkertijd eerdere onderzoeksresultaten te bevestigen en tegenstrijdige conclusies te verklaren. Meer bepaald verschaften onze analyses een verklaring waarom de disk diffusietest voor *Pasteurellaceae* door Hubert *et al.* (1989) als onvoldoende werd beoordeeld, terwijl dit door Citron *et al.* (2005) meer recent als een goede test werd beschouwd. Onze verklarende analyses hebben aangetoond dat wanneer resistentie weinig voorkomt bij respiratoire *Pasteurellaceae*, deze manier van gevoeligheidsbepalingen zeer betrouwbaar is. Wanneer resistentie veelvuldig voorkomt, dient voor sommige kiem-antibioticumcombinaties enige voorzichtigheid te worden geboden.

Er kan besloten worden dat de diffusietest voor *Pasteurelleceae* goed bruikbaar is indien weinig resistentie wordt verwacht, zoals werd aangetoond in de extensief gehouden kalveren. Wanneer resistentie bij *Pasteurellaceae* veelvuldig voorkomt zoals aangetoond bij de vleeskalveren, dienen de resultaten met enige voorzichtigheid te worden geïnterpreteerd.

5. VERBANDEN TUSSEN ANTIBIOTICUMGEBRUIK EN RESISTENTIE

5.1. Studiepopulatie

Vermits de grote hoeveelheid foutief geïdentificeerde Gram-positieve indicatorbacteriën (streptokokken/enterokokken), werden deze voor de analyse van het verband tussen antibioticumgebruik en resistentie niet gebruikt. Deze analyse werd bijgevolg verricht op enterische *E. coli* afkomstig van 15 bedrijven (5 melkvee, 5 vleesvee-, en 5 vleeskalverenbedrijven) enerzijds, en respiratoire *Pasteurellaceae* afkomstig van 5 zoogkoeien- en 5 vleeskalverenbedrijven anderzijds.

De gebruikte parameters voor het antibioticumgebruik zijn de behandelingsincidenties TI_{ADD} en TI_{UDD}, die respectievelijk de behandelingsincidenties van antibiotica volgens de bijsluiter voor de hoofdindicatie, en de behandelingsincidentie van de antibiotica zoals werkelijk toegediend op het bedrijf weerspiegelen (zie hoofdstuk 2). Een beknopt overzicht van het antibioticumgebruik op de 5 melkvee-, de 5 vleesvee- en de 5 vleeskalverenbedrijven die in dit onderzoeksluik betrokken waren, wordt hernomen in Tabel 1. Een TI_{ADD} van 104.5 (vleeskalverenbedrijven) betekent dat op 1000 dieren, er elke dag van de observatieperiode gemiddeld 104.5 runderen werden behandeld met een standaarddosis. Een TI_{UDD} van 272.0 geeft aan dat in feite 272.0 dieren werden behandeld. Met andere woorden; er werden op dit type bedrijven, met een bepaalde hoeveelheid antibioticum veel meer dieren behandeld dan voorgeschreven volgens de bijsluiter.

Tabel 1. Totale $TI_{ADDcattle}$ en $TI_{UDDcattle}$ (min-max) voor de 3 verschillende bedrijfstypes per toedieningsweg.

	**Melkveebedrijven N=5**		**Zoogkoeienbedrijven N=5**		**Vleeskalverenbedrijven N=5**	
	**$TI_{ADDcattle}$**	**$TI_{UDDcattle}$**	**$TI_{ADDcattle}$**	**$TI_{UDDcattle}$**	**$TI_{ADDcattle}$**	**$TI_{UDDcattle}$**
Oraal	–	–	0.77[a] (0.00-2.42)	0.77[a, b] (0.00-2.42)	97.78 (45.08-365.66)	266.81 (120.30-611.98)
Intramammair	2.83 (1.81-3.70)	2.71 (0.94-3.83)	0.09 (0.00-0.49)	<0.09 [b] (0.00-0.49)	NA[c]	NA[c]
Intra-uterien[a]	0.42 (0.00-1.27)	0.32 (0.00-1.11)	0.78 (0.38-1.08)	1.07 (0.39-1.62)	NA[c]	NA[c]
Parenteraal	3.01 (0.25-7.16)	2.36 (0.25-7.11)	3.78 (1.94 -6.94)	2.15 (1.41-3.76)	6.74 (0.59-17.59)	5.21 (0.48-14.92)
**Totaal**	**6.26 (3.23-10.86)**	**5.39 (2.85-10.94)**	**5.42 (3.02-10.15)**	**4.89 (2.25-7.16)**	**104.52 (45.08-369.66)**	**272.02 (120.30-626.90)**

[a] voor intramammaire en intra-uteriene behandelingen en voor orale bolussen op de zoogkoeienbedrijven werd de $TI_{UDcattle}$ gebruikt ipv de $TI_{ADDcattle}$ [b] UDD kon niet berekende worden voor de orale bolussen en de intramammaire behandelingen op de zoogkoeienbedrijven. Daarom werd de TI_{UD} waarde gebruikt om de totale TI_{UDD} te berekenen. [c] niet van toepassing

Voor de selectie van de bedrijven, de registratie en de gedetailleerde analyse van het antibioticumgebruik wordt verwezen naar hoofdstuk 2.

5.2. Het spijsverteringsstelsel: *E. coli*

Voor wat betreft de staalnames, de isolatie- en identificatieprocedure, alsook de methodiek van de gevoeligheidsbepalingen wordt verwezen naar hoofdstuk 3. De antimicrobiële resistentie index (ARI), die als maatstaf voor het voorkomen van antibioticumresistentie in dit onderzoeksluik werd gebruikt, is gedefinieerd als het aantal resistentiewaarnemingen op het totaal van gevoeligheidsbepalingen per kiem (Catry et al., 2005). Intermediaire resultaten werden hierbij als resistent beschouwd.

Het verband tussen het antibioticumgebruik en resistentie (antimicrobiële resistentie index, ARI= N resistentiewaarnemingen op totaal aantal gevoeligheidsbepalingen) werd op 15 bedrijven geanalyseerd voor *E. coli* (N= 1509, totaal aantal *E. coli* isolaten). Respectievelijk waren deze isolaten afkomstig van 5 melkvee- (3 staalnames, N=635), 5 vleesvee- (2 staalnames, N=367), en 5 vleeskalverenbedrijven (twee tijdstippen T1 en T2, N=507). Een overzicht van het voorkomen van resistentie, inclusief de gemiddelde antimicrobiële resistentie index (ARI) per staalronde van bedrijven die in dit onderzoeksluik werden opgenomen, wordt weergegeven in Tabel 2.

Het verband tussen het antibioticumgebruik, gekarakteriseerd door de totale TI_{ADD} of TI_{UDD} (som van alle toedieningswegen), en het voorkomen van resistentie gekarakteriseerd door de ARI, werd onderzocht d.m.v. een lineair gemengd model, met het bedrijf als random effect en behandelingsincidentie, bedrijfstype en leeftijd als onafhankelijke variabelen.

In de multivariate analyse werd een significant verband aangetoond tussen enerzijds de TI_{ADD} ($P<0.001$) en anderzijds de TI_{UDD} ($P<0.001$) en de ARI van *E. coli.* Hierbij was het bedrijfstype ook steeds significant ($P<0.001$). De leeftijd van de dieren (uitgedrukt in maanden) daarentegen bleek geen significant effect te hebben op de ARI. Deze resultaten tonen aan dat er een groot verschil is tussen de bedrijfstypes in het voorkomen van resistentie maar dat bovendien, los van het bedrijfstype, er een sterk verband bestaat tussen de hoeveelheid toegediende antibiotica en het resistentieniveau.

Tabel 2. Overzicht van resistentiepercentages en ARI van *E. coli* gedurende de verschillende tijdstippen.

Productie-type	staalname (N kuddes)	N. Isolaten	ARI[a]	AMP[b]	AMC	CEF	TET	TMP	NEO	GEN	SPE	STR	NAL	FLU	ENRO
**Melkvee**	I (10)	447	0,04	2,91	0,45	0,45	8,28	4,25	0,67	1,12	0,22	24,83	1,34	0,22	0
	II (10)	396	0,01	2,02	0,25	0	3,79	0,25	1,52	0	0,25	4,55	0,76	0,25	0,25
	III (10)	419	0,02	4,3	0,24	0	4,3	3,58	2,15	0,48	0	7,88	1,19	0,72	0,24
	IV (10)	359	0,01	2,79	0,28	0,28	3,06	0,84	0,84	0	0	4,74	0,28	0,28	0,28
	V (10)	382	0,02	5,24	1,05	0,26	3,66	3,56	0,52	1,05	0,79	8,64	0,79	0,26	0,26
	VI (10)	370	0,02	3,24	0,54	0	5,14	1,89	1,62	1,08	0,54	6,76	1,35	0,54	0,27
**Zoogkoeien**	I (10)	436	0,03	9,17	1,15	0	6,88	4,13	2,52	0,92	0,69	13,3	2,52	0,46	0,46
	II (9)	346	0,06	12,14	1,45	0,58	17,05	5,49	4,91	2,31	0,87	18,21	8,67	4,33	2,89
	III (9)	298	0,05	9,4	1,34	0,34	12,08	6,71	5,03	1,01	0,34	13,76	5,03	4,36	2,35
	IV (7)	215	0,09	17,21	1,4	0	15,81	6,05	7,45	2,33	1,4	24,19	8,37	5,12	4,19
**Vleeskalv.**	T1 (5)	276	0,62	93,12	4,71	0,36	94,93	92,75	83,33	45,29	22,46	89,49	0,79	0,73	0,64
	T2 (5)	230	0,32	79,57	2,61	1,74	95,22	65,22	27,83	5,22	5,65	78,26	0,14	0,06	0,04
**Totaal**	(25)	4174	0,09	17,08	1,24	0,26	19,03	13,75	9,67	4,52	2,37	22,02	8,74	6,70	5,58

[a] ARI: gemiddelde antimicrobial resistance index (ARI) per staalname. [b] AMP, ampicilline; AMC, amoxicilline + clavulaanzuur; CEF, ceftiofur; TET, oxytetracycline; TMP, trimethoprim-sulphonamiden; NEO, neomycine; GEN, gentamicine; SPE, spectinomycine; STR, streptomycine; NAL, nalidixine zuur; FLU, flumequine; ENR, enrofloxacine.

5.3. Het ademhalingsstelsel: respiratoire *Pasteurellaceae*

De relatie tussen behandelingsincidenties (TI_{ADD} en $TI_{UDD)}$ en antimicrobiële resistentie index (ARI= N resistentiewaarnemingen op totaal aantal gevoeligheidsbepalingen) werd onderzocht voor 309 *Pasteurellaceae* van 5 vleesvee- (2 staalnames, N=102) en 5 vleeskalverenbedrijven (T1 and T2, N=207). Een overzicht van het voorkomen van resistentie in deze dataset, inclusief de gemiddelde ARI per momentopname wordt weergegeven in Tabel 3.

Analoog aan de analyse voor de fecale *E. coli's*, werd het verband tussen het antibioticumgebruik en het voorkomen van resistentie onderzocht met behulp van een lineair gemengd model, met het bedrijf als random effect en behandelingsincidentie, bedrijfstype en leeftijd als onafhankelijke variabelen.

Over het bedrijfstype heen werd een significant verband gevonden tussen de TI_{UDD} (P=0.002) en de ARI van de *Pasteurellaceae.* Voor de analyse met de TI_{ADD} werd een borderline niet significant effect gevonden (P=0.093). Ook in deze analyses had het bedrijfstype steeds een significant effect op de ARI, terwijl ook hier voor de leeftijd geen effect werd gevonden.

Het verschil in significantie tussen enerzijds TI_{UDD} en anderzijds TI_{ADD} en de ARI, zoals ondervonden tijdens de analyse in het ademhalingsstelsel, is mogelijks het gevolg van de grotere mate van nauwkeurigheid waarmee de behandelingsincidentie wordt beschreven met behulp van de TI_{UDD} in vergelijking met de $TI_{ADD.}$ Dit komt duidelijker tot expressie in deze analyse gezien het gering aantal stammen in vergelijking met de fecale *E. coli* isolaten.

Tabel 3. Overzicht van resistentiepercentages en ARI van *Pasteurellaceae* gedurende de verschillende tijdstippen.

Productie- type	staalname (N kuddes)	N. Isolaten	ARI[a]	AMP[b]	AMC	CEF	TET	TMP	NEO	GEN	SPE	FLO	NAL	FLU	E[illegible]
**Zoogkoeien**	I (10)	94	0,03	0,00	0,00	0,00	0,00	17,78	1,11	1,11	15,56	0,00	1,11	1,11	[illegible]
	II (9)	78	0,01	1,49	0,00	0,00	0,00	2,99	0,00	0,00	10,45	0,00	0,00	0,00	[illegible]
	III (9)	42	0,03	0,00	0,00	0,00	0,00	2,63	0,00	0,00	28,95	0,00	0,00	0,00	[illegible]
	IV (7)	54	0,02	0,00	0,00	0,00	0,00	0,00	2,04	2,04	24,49	0,00	0,00	0,00	[illegible]
**Vleeskalv.**	T1 (5)	93	0,27	8,60	0,00	0,00	38,71	50,54	45,16	43,01	4,30	0,00	48,39	44,09	3[illegible]
	T2 (5)	113	0,15	29,82	0,00	0,00	42,98	29,82	18,42	14,91	1,75	0,00	19,30	16,67	1[illegible]
**Totaal**	(15)	474	0,11	9,53	0,00	0,00	18,85	22,17	14,41	13,08	11,09	0,00	15,08	13,53	1[illegible]

[a] ARI: gemiddelde antimicrobial resistance index (ARI) per staalname. [b] AMP, ampicilline; AMC, amoxicilline + clavulaanzuur; CEF, ceftiofur; TET, oxytetracycline; TMP, trimethoprim-sulphonamiden; NEO, neomycine; GEN, gentamicine; SPE, spectinomycine; FLO, florfenicol; NAL, nalidixine zuur; FLU, flumequine; ENR, enrofloxacine.

5.4. Het voorkomen van resistentie in twee ecosystemen

Enterokokken en streptokokken werden wegens de lage prevalentie en de onnauwkeurigheid van zowel de identificatie als de gevoeligheidsbepalingen niet betrokken in dit onderzoeksluik.

Om de relatie tussen aanwezigheid van resistentie voor 11 antibiotica te vergelijken tussen gepaarde *E. coli* en *Pasteurellaceae* isolaten uit eenzelfde dier werden Odds ratio's berekend. Hierbij werden de resistenties bij de *Pasteurellaceae* als afhankelijke variabele beschouwd, met andere woorden er werd berekend hoe groot de *Odds* waren om resistentie tegen een bepaald antibioticum te vinden bij de *Pasteurellaceae* uit het ademhalingsstelsel als ook de *E. coli* isolaten uit het spijsverteringsstelsel voor datzelfde product resistentie vertoonden (Figuur 1). Voor nalidixine zuur (NAL; OR=8.5 (3.0-24.4)), flumequine (FLU; OR=9.8 (3.1-30.5)), enrofloxacine (ENR; OR=13.2 (3.7-47.3)), en gentamicine (OR=6.3 (2.6-15.2)) werd een duidelijk significant verband ($P<0.001$) gevonden tussen het voorkomen van resistentie bij de *E. coli*'s en de *Pasteurellaceae*. Voor wat betreft neomycine werd een tendens ($P=0.103$) gezien (OR=2.0 (0.9-4.7). Verbanden tussen *E. coli* en *M. haemolytica*, waren niet significant ($P\geq0.07$), hoewel vergelijkbare tendensen werden aangetoond voor de quinolones (NAL, OR=4.4 (0.9-21.6); FLU, OR=2.8 (0.6-13.8); ENR OR=2.2 (0.4-11.9)). De meerstapsresistenties door mutaties zijn kenmerkend voor quinolones, en kunnen deze resultaten deels verklaren.

Er werd geen significant verband aangetoond tussen het voorkomen van resistentie in de voor wat betreft de resistentie tegen ampicilline, amoxicilline-clavulaanzuur, ceftiofur, tetracycline, trimetoprim-sulfonamiden, en spectinomycine.

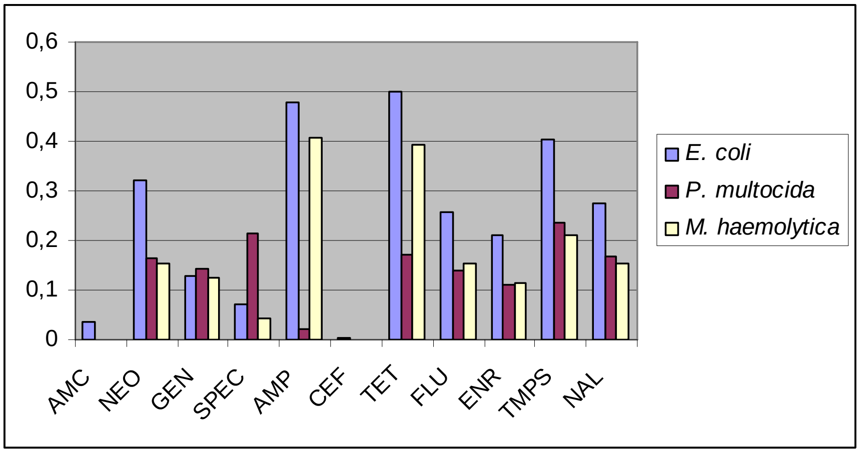

Figuur 1. Overzicht van resistentiepercentages (0.1=10%) van de onderzochte kiemen voor amoxicilline+clavulaanzuur (AMC), neomycine (NEO), gentamicine (GEN), spectinomycine (SPEC), ampicilline (AMP), ceftiofur (CEF), tetracycline (TET), flumequine (FLU), enrofloxacine (ENR), trimethoprim + sulphonamiden (TMPS), nalidixine zuur (NAL).

5.5. Discussie

De gevonden verbanden tussen het antibioticumgebruik en de resistentie bij fecale *E. coli* bevestigen dat ook in de rundveesector, het toedienen van antibiotica wordt weerspiegeld in de graad van antimicrobiële resistentie in de enterische microbiota van de dieren. Dit werd gesuggereerd op basis van andere onderzoeken bij varkens en pluimvee (Catry *et al.*, 2003) en recent ook aangetoond voor tetracycline resistentie bij varkens in een vergelijkbare epidemiologische studie (Dewulf et al., 2006). Door het innoverende luik, dat inhield dat tevens de respiratoire facultatief pathogene *Pasteurellaceae* werden ingesloten, kunnen bijkomende conclusies worden getrokken: 1) ook in het ademhalingsstelsel resulteert de selectiedruk uitgeoefend door het antibioticumgebruik in resistentieontwikkeling bij de aanwezige organismen, 2) het inzetten van een nieuwe parameter om de incidentie van antibioticumbehandelingen te kwantificeren (TI_{UDD}) heeft toegelaten om de verbanden tussen antibioticumgebruik en resistentieontwikkeling nog beter te beschrijven.

Het verband tussen de simultane aanwezigheid van bepaalde resistenties in fecale *E. coli* isolaten en respiratoire *Pasteurellaceae* binnen eenzelfde dier, werd slechts voor een beperkt aantal antibiotica aangetoond. Toch kan voor deze antimicrobiële middelen (bijvoorbeeld voor de quinolones) een gericht advies gegeven worden met betrekking tot de aanwezigheid van resistentie in respiratoire pathogenen wanneer dit wordt vastgesteld in fecale *E. coli*. Voor de meerderheid van de onderzochte antibiotica was dit evenwel niet het geval. Andere factoren, zoals gelinkte resistentiegenen specifiek voor een bepaalde bacterie en een andere farmacokinetiek in het ademhalingsstelsel versus het spijsverteringsstelsel kunnen dit allicht mede verklaren.

6. MINIMAAL BEMONSTERINGSPROTOCOL

Het doel van het ontwikkelen van een minimaal bemonsteringsprotocol op bedrijfsniveau is om een betrouwbare opmeting van de antimicrobiële resistentie te waarborgen, met een minimum aan middelen (dieren, stalen, antibiotica). Voor wat betreft de reductie van het aantal staalnames en het aantal te onderzoeken antimicrobiële middelen, werden de analyses gedaan op de vleeskalverenbedrijven aan het begin van de productieronde. De motivatie hiervoor is het veelvuldig voorkomen van resistentie en multi-resistentie op vleeskalverenbedrijven, terwijl beide schaars waren op de melkvee- en zoogkoeienbedrijven. Bijgevolg wordt dus getracht een minimaal bemonsteringsprotocol te suggereren op die bedrijven waar de resistentieproblematiek zowel in het spijsverteringsstelsel als het ademhalingsstelsel het grootst is. Voor de analyses werd gebruik gemaakt van fecale *E. coli*, vermits zij in nagenoeg alle dieren kunnen worden geïsoleerd, en zowel de identificatie als de gevoeligheidsbepalingen erg betrouwbaar zijn. Bijgevolg kan het aantal dieren gelijkgesteld worden aan het aantal staalnames.

Voor wat betreft de staalnames, de isolatie- en identificatieprocedure, alsook de methodiek van de gevoeligheidsbepalingen wordt verwezen naar hoofdstuk 3. De antimicrobiële resistentie index (ARI), die als maatstaf voor het voorkomen van antibioticumresistentie in dit onderzoeksluik werd gebruikt, is gedefinieerd als het aantal resistentiewaarnemingen op het totaal van gevoeligheidsbepalingen per kiem (Catry et al., 2005a). Intermediaire resultaten werden hierbij als resistent beschouwd.

6.1. Reductie van het aantal staalnames

Op vleeskalverenbedrijven werd initieel telkens 20% van de dieren rectaal bemonsterd (zie hoger), waaruit *E. coli* werd geïsoleerd en een antibiogram voor 12 antibiotica werd aangelegd (Kirby Bauer disk diffusie). Voor elke *E. coli* was bijgevolg een ARI voorhanden, waarna een bedrijfsgemiddelde voor ieder deelnemend beslag kon worden berekend. Het doel van dit onderzoeksluik was na te gaan, in hoeverre de foutenmarge toeneemt, wanneer respectievelijk slechts 10, 5, 2 en 1% van de kalveren zou zijn bemonsterd.

Door middel van bootstrapanalyse (@risk) werd uit de aanwezige dataset van elk bedrijf telkens 1000x een willekeurige steekproef genomen, waarna een 95% betrouwbaarheidsinterval kon worden berekend. In Figuur 1 worden twee kenmerkende voorbeelden gegeven van de bekomen betrouwbaarheidsintervallen. Hieruit kan worden geconcludeerd dat de toename van de onzekerheid bij de schatting van de ARI relatief beperkt is bij een beperking van het aantal stalen tot 5% van de aanwezige dieren. De onzekerheid neemt daarentegen enorm toe wanneer het aantal stalen van 5 naar 2% wordt gereduceerd. Bovendien kan uit de grafiek duidelijk worden opgemaakt dat de onzekerheid rond de ARI niet symmetrisch verdeeld is. Dit houdt in dat de traditionele manier om betrouwbaarheidsintervallen te berekenen eigenlijk niet correct is voor dit soort data. Dit bijzonder aspect wordt echter in de meeste resistentiemonitoringsprogramma’s verwaarloosd met betrekking tot de statistische analyse.

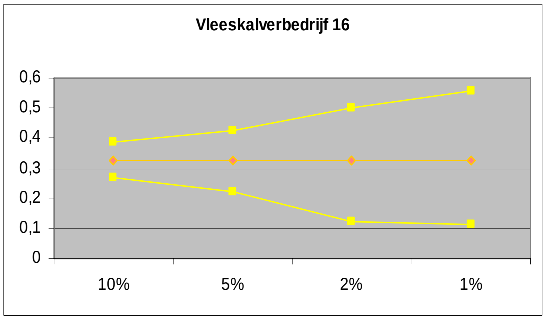

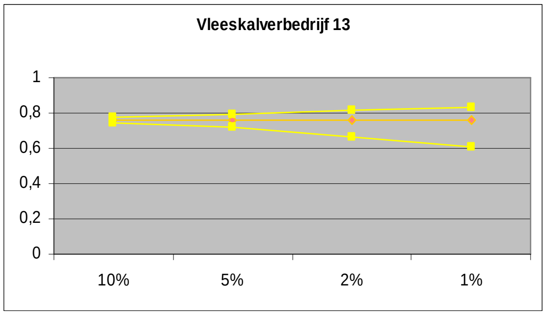

Figuur 1. Invloed van een reductie van het aantal staalnames (10, 5, 2, of 1%) op het betrouwbaarheidsinterval rond de gemiddelde ARI (antimicrobial resistance index) gebaseerd op gevoeligheidsbepalingen van 12 antibiotica voor *E. coli*. Zowel een bedrijf met een hoge (links) als een bedrijf (rechts) met een zeer hoge gemiddelde bedrijfsARI worden getoond.

6.2. Reductie van het aantal te testen antibiotica

De oorspronkelijke monitoring bestond voor elke geïsoleerde *E. coli* uit gevoeligheidsbepalingen voor 12 antibiotica. Praktisch komt dit neer op 2 agarbodems die worden geïnoculeerd. Een efficiënte reductie van het aantal antibiotica is bijgevolg het herleiden van deze 2 agarbodems tot 1, hetgeen impliceert dat slechts 7 antibiotica worden getest. Bijgevolg was de doelstelling van dit luik, de invloed na te gaan van een reductie van 12 naar 7 antibiotica op de gemiddelde ARI per bedrijf op elk tijdstip. Zoals voorheen vermeld, werden ook hier de vleeskalverenbedrijven voor gebruikt. De keuze van 7 uit 12 antibiotica is gebaseerd op het selecteren van een vertegenwoordiger van elke klasse, het uitsluiten van vaak voorkomende kruisresistenties, en de grootste standaarddeviatie op de prevalentie per antibioticum. Volgende antibiotica werden gebruikt: ampicilline (betalactams), oxytetracycline (tetracyclines), trimethoprim-sulphonamiden (gepotentieerde sulfonamiden), enrofloxacine (quinolones), neomycine, gentamicine (aminoglycosiden), spectinomycine (aminocyclitolen).

In Figuur 2 wordt een vergelijking gemaakt van de gemiddelde bedrijfswaarde voor de ARI, enerzijds gebaseerd op 12 antibiotica en anderzijds na reductie tot 7 antibiotica. Hieruit blijkt dat de gemiddelde ARI circa 0.1 afneemt door het verminderen van het aantal antibiotica, maar dat de bedrijven analoog kunnen worden gecatalogeerd.

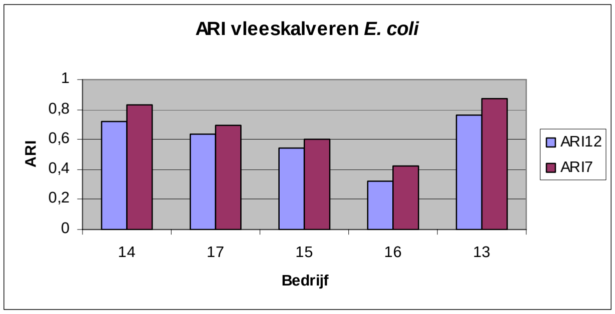

Figuur 2. Invloed van een reductie van 12 (ARI12) naar 7 (ARI7) antibiotica op de gemiddelde ARI op 5 vleeskalverenbedrijven.

6.3. Discussie

De analyses suggereren dat een hoge mate van betrouwbaarheid kan worden gewaarborgd wanneer 5 % van de dieren wordt bemonsterd voor de isolatie van *E. coli*, en wanneer op deze isolaten de volgende 7 antibiotica worden getest: ampicilline, oxytetracycline, trimethoprim-sulphonamiden, enrofloxacine, neomycine, gentamicine, spectinomycine.

Teneinde de betrouwbaarheid van slechts 7 antibiotica voor de monitoring van resistentie verder te bevestigen, werd het verband tussen het antibioticumgebruik (TI_{ADD} en $TI_{UDD)}$ en het voorkomen van resistentie (ARI) bepaald, uitgevoerd op 15 bedrijven zoals hoger beschreven, hernomen wanneer slechts 7 antibiotica de ARI determineren (ongepubliceerde data). Hieruit bleek dat ook met slechts 7 antibiotica, het verband tussen ARI en de behandelingsincidenties over de drie productietypes heen telkens significant was (ARI7 met TI_{ADD}; $P<0.01$, ARI7 met TI_{UDD} ($P<0.01$). Met andere woorden, de voorgestelde reductie van 12 naar 7 antibiotica heeft niet tot gevolg dat het verband met het antibioticumgebruik niet meer kan worden aangetoond. Ook dit onderzoek suggereert dat door slechts deze 7 antibiotica te testen op fecale *E. coli*, toch een hoge mate van betrouwbaarheid kan worden gewaarborgd voor het opmeten van de aanwezige graad van antibioticumresistentie op rundveebedrijven.

Bij het opstellen van een minimaal bemonsteringsprotocol voor resistentiebewaking op rundveebedrijven, dient vooreerst aandacht besteed te worden aan het type van bedrijf. Tot op heden wordt in de design van surveillanceprogramma's geen rekening gehouden met de specifieke verschillen die er zijn tussen de verschillende productietypes. Het onderhavige onderzoek heeft echter heel duidelijk aangetoond dat het bedrijfstype steeds een heel bepalende rol speelt in de mate van voorkomen van resistentie. Vermits binnen de drie bedrijfstypes, de resistentiesituatie duidelijk ongunstig was voor de vleeskalverenbedrijven en dit zowel in het spijsverteringsstelsel als in het ademhalingsstelsel, verdient deze sector bijzondere aandacht in het opstellen van monitoringsprogramma's. Dit onderzoek heeft dan ook duidelijk aangetoond dat de interpretatie van testresultaten en de ontwikkeling van bemonsteringsschema's best specifiek worden gedaan in functie van de te verwachten resistentiesituatie.

Terwijl de prevalentie, identificatie en de gevoeligheidsbepalingen van de indicatorbacterie *S. bovis* zoals onderzocht met de toegepaste methodologie ontoereikend bleken in dit onderzoek, is dit niet het geval voor fecale *E. coli*. Zowel de prevalentie, de nauwkeurigheid van de identificatie en gevoeligheidsbepalingen worden voldoende geacht. De gevonden verbanden met het antibioticumgebruik tonen bovendien aan dat het een bijzonder interessante indicatorkiem is om de epidemiologie van antibioticumresistentie te bestuderen.

Wanneer wordt geopteerd voor *E. coli*, kan het aantal isolaten dat dient te worden geïsoleerd in een monitoringsprogramma op bedrijfsniveau worden gelijkgesteld met het aantal dieren, vanwege de aanwezigheid van *E. coli* in quasi elk dier. Daarnaast suggereren onze resultaten dat het bemonsteren van 20, 10 of 5 % van de dieren een zekere betrouwbaarheid waarborgt voor het opmeten van antibioticumresistentie, die evenwel aanzienlijk daalt bij een reductie naar 2 of 1% van de dieren.

Teneinde de middelen van een monitoringsprogramma zo efficiënt mogelijk in te zetten, wijzen onze resultaten op het feit dat het geselecteerd reduceren van het aantal antibiotica van 12 naar 7 producten, toch nog een grote mate van betrouwbaarheid kan waarborgen. Hierbij dient er voor elke klasse antibiotica een vertegenwoordiger te worden gekozen. De informatie van 7 producten wordt voldoende geacht voor een eerste screening,

terwijl bijkomende onderzoeken kunnen ingezet worden voor het opmeten van opkomende resistentie (emerging resistances, bijvoorbeeld cephalosporines) wanneer multi-resistentie wordt aangetoond tijdens de eerste screening.

Op basis van dit onderzoeksproject, kan ook het insluiten van *Pasteurellaceae* en meerbepaald *P. multocida* in monitoringsprogramma's worden overwogen als alternatief voor *S. bovis*. De volgende aspecten spreken hierbij in het voordeel van de *P. multocida*: 1. de aanzienlijk hogere prevalentie bij gezonde dieren, 2. het facultatief pathogene karakter, voor zowel rundvee als andere diersoorten (varkens). Hierdoor kunnen sensibilisatiecampagnes voor dierenartsen en veehouders gerichter verlopen vermits het verband met economische verliezen concreter kan worden toegelicht. 3. de intrinsieke gevoeligheid voor antibiotica met hoofdzakelijk een Gram-positief spectrum (o.a. penicillines, macroliden). *E. coli* is immers van nature uit ongevoelig voor deze producten, waardoor de resistentieselectiedruk voor deze producten niet kan worden geëvalueerd.

Samenvatting

Antibioticumresistentie van bacteriën kan leiden tot therapiefalen, een langere behandelingsduur en een hogere mortaliteit als gevolg van bacteriële ziekten. De resistentie kan eveneens worden uitgewisseld tussen dier en mens. Om die reden is het van cruciaal belang voor zowel de diergeneeskunde als de volksgezondheid om het niveau van antibioticumresistentie zo laag mogelijk te houden. Ter controle van het volksgezondheidsrisico werden in verschillende landen monitoringprogramma's opgestart. Enerzijds wordt hierin het humaan en veterinair antibioticumgebruik globaal geregistreerd. Anderzijds wordt het antibioticumresistentieniveau bepaald bij humane pathogenen, zoönotische bacteriën en (commensale) indicatorbacteriën van nutsdieren. De globale gegevens van monitoringprogramma's, veelal gebaseerd op verkoopcijfers en ongedetailleerde resistentiepercentages, bieden niet de mogelijkheid om te worden teruggekoppeld naar individuele landbouwbedrijven waar de selectiedruk (antibioticumgebruik) heerst. Dit project trachtte de epidemiologie van antibioticumresistentie op Belgische rundveebedrijven grondig te onderzoeken teneinde een bemonsteringsprotocol op kuddeniveau te ontwikkelen, waardoor op een efficiënte manier bedrijven kunnen worden geklasseerd en opgevolgd in de tijd. Hierbij stonden vier doelstellingen centraal.

De eerste doelstelling betrof het geven van een gedetailleerd overzicht van het antibioticumgebruik in de Vlaamse rundveehouderij, door een viermaandelijkse opmeting in 5 melkvee-, 5 vleesvee-, en 5 vleeskalverenbedrijven. De gebruikte methodologie maakte het mogelijk om een kwantitatieve én kwalitatieve beschrijving van de toedieningen van antibiotica te maken op dierniveau, voortbouwend op een registratiesysteem dat werd ontwikkeld door de wereld gezondheidsorganisatie (WHO, defined daily dose). Hierdoor is een vergelijking op internationaal niveau mogelijk. Op basis van de metingen kan worden besloten dat gemiddeld (min-max) per 1000 aanwezige dieren er dagelijks op melkvee- en vleesveebedrijven, respectievelijk 6,26 (3,23-10,86) en 5,42 (3,02-10,15) runderen worden behandeld (TI_{UDD}). Bovendien werd vastgesteld dat deze toedieningen weinig afweken van de aanbevolen doseringen (TI_{ADD}). Op de vleeskalverenbedrijven, werden per 1000 aanwezige dieren, gemiddeld 272,02 (120,30-626,90) kalveren dagelijks behandeld (TI_{UDD}), hoofdzakelijk via orale groepsbehandelingen ter controle van ademhalingsstoornissen. Deze behandelingen weken echter aanzienlijk af van de aanbevolen doseringen volgens de bijsluiters van de toegediende producten, vermits de aanwezige hoeveelheden slechts bestemd waren voor 104,52 (45,08-369,66) kalveren per 1000 (TI_{ADD}). Met andere woorden, bijna driemaal meer vleeskalveren werden behandeld dan voorzien volgens de aanbevolen regimes.

Een tweede doelstelling was het accuraat opmeten van antibioticumresistentiesituatie op Vlaamse rundveebedrijven in de commensale enterische microbiota door middel van indicatorbacteriën, bij facultatief pathogenen van het ademhalingsstelsel en bij de zoönotische kiem *Salmonella*. De antibiotica die initieel werden ingesloten in het onderzoek waren benzylpenicilline, ampicilline, amoxicilline-clavulaanzuur, ceftiofur, erythromycine, lincomycine, tetracycline, neomycine, gentamicine, spectinomycine, streptomycine, nalidixinezuur, flumequine, enrofloxacine en de combinatie sulphonamiden/trimethoprim.

Uit de oorspronkelijke opmeting van resistentie bleek dat de gebruikelijke Gram positieve indicatorbacteriën *Enterococcus (E.) faecium* en *E. faecalis* aan een te lage frequentie (range van gemiddelde bedrijfsprevalentie: 0,4-12%) werden geïsoleerd om tendensen op te volgen. Als alternatief werd daarom *Streptococcus (S.) bovis* geïntroduceerd als indicatorbacterie voor rundvee. Ondanks een hoge initiële prevalentie op basis van fenotypische identificatie (range van gemiddelde bedrijfsprevalentie: 25,8-73,0%), bleek na

controle door het vergelijken van de kiemtypering met moleculaire technieken (tDNA-PCR) dat slechts 63,4% van de isolaten correct was geïdentificeerd. Eveneens bleek de kwaliteit van de gebruikte gevoeligheidstesten (disk diffusietesten) ontoereikend om enerzijds accuraat tendensen op te volgen en anderzijds verbanden aan te tonen met het antibioticumgebruik. De sensitiviteit en specificiteit van de disk diffusie gevoeligheidstesten werden onderzocht door het vergelijken van de testresultaten met die van de agar dilutiemethode die als *gouden standaard* werd beschouwd. Vervolgens werden respectievelijk de resistent (positief; *resistant predictive value*, RPV) en gevoelig (negatief; *susceptible predictive value*, SPV) voorspellende waarde van de disk diffusietesten berekend. De Gram positieve kiemen uit het spijsverteringsstelsel werden niet betrokken in latere analyses van dit project.

De kwaliteit van zowel de bacteriële identificatie als de gevoeligheidsbepalingen werd analoog tevens gecontroleerd voor de facultatief pathogene *Pasteurellaceae* uit het ademhalingsstelsel (*Pasteurella multocida* en *Mannheimia haemolytica* sensu lato). Hier bleek een goede kwaliteit van de fenotypische identificatie (> 93,5%) en ook het merendeel van de onderzochte bacterie-antibioticumcombinaties van de disk diffusietesten vertoonden een goede betrouwbaarheid. Meerbepaald waren alle 14 berekende RPV groter dan 85 % en 7 van de 10 berekenbare SPV groter dan 90 %.

Uit geen enkel staal van de 278 meststalen genomen aan het begin van een productieronde op 5 vleeskalverenbedrijven, kon *Salmonella* geïsoleerd worden.

Op basis van de opgemeten antibioticumresistenties in zowel de fecale *E. coli* isolaten als de respiratoire *Pasteurellaceae*, kon eenzelfde algemene tendens worden vastgesteld: op melkvee- en vleesveebedrijven is de resistentiesituatie gunstig (weinig voorkomend) en stabiel, terwijl op de vleeskalverenbedrijven de situatie ongunstig is (hoge frequentie van resistentie en multiresistentie) en dynamisch. Het dynamische aspect omvat een aanzienlijke wijziging in het voorkomen van resistentieprofielen aan het einde van de productieronde in vergelijking met het begin van de productieronde.

De derde centrale doelstelling van dit project was om verbanden tussen het antibioticumgebruik en de resistentiesituatie na te gaan. Vanwege de goede accuraatheid van de monitoring van fecale *E. coli* en respiratoire *Pasteurellaceae* werden de analyses op deze twee ecosystemen voltrokken. Er werd telkens een lineair gemengd model gebruikt voor de analyse, met het bedrijf als *random* effect en behandelingsincidentie, bedrijfstype en leeftijd (uitgedrukt in maanden) als onafhankelijke variabelen. Het antibioticumgebruik werd weergegeven door enerzijds de behandelingsincidenties die aangeven wat gebruikt werd indien de aanbevolen behandelingsdosis wordt gerespecteerd (TI_{ADD}), en anderzijds aangepast naar wat werkelijk werd gebruikt op het bedrijf (TI_{UDD}).

Het verband tussen het antibioticumgebruik (totale TI_{ADD} of TI_{UDD}, som van alle toedieningswegen), en de resistentie (ARI = N resistentiewaarnemingen op totaal aantal gevoeligheidsbepalingen) werd op 15 bedrijven geanalyseerd voor fecale *E. coli* isolaten (N= 1509, totaal aantal *E. coli* isolaten). In de multivariate analyse werd een significant verband aangetoond tussen enerzijds de TI_{ADD} (P<0.001) en anderzijds de TI_{UDD} (P<0.001) en de ARI van *E. coli*. Hierbij was het bedrijfstype ook steeds significant (P<0.001), terwijl de leeftijd geen effect vertoonde.

De relatie tussen behandelingsincidenties (totale TI_{ADD} en $TI_{UDD)}$ en antimicrobiële resistentie index (ARI) werd onderzocht voor 309 *Pasteurellaceae* van 5 vleesvee- (2 staalnames, N=102) en 5 vleeskalverenbedrijven (T1 and T2, N=207). Over het bedrijfstype heen werd een significant verband gevonden tussen de TI_{UDD} (P=0.002) en de ARI van de *Pasteurellaceae*. Voor de analyse met de TI_{ADD} werd een borderline niet significant effect gevonden (P=0.093). Ook in deze analyses had het bedrijfstype steeds een significant effect op de ARI, terwijl ook hier voor de leeftijd geen effect werd gevonden.

De vierde centrale doelstelling was het voorstellen van een bemonsteringsstrategie, die moet toelaten om via een accurate en efficiënte (goedkope) methodologie bedrijven te kunnen catalogeren in ongunstige en gunstige beslagen met betrekking tot de antibioticumresistentiesituatie. Hiertoe werden simulaties verricht van reducties van het werkelijk uitgevoerde bemonsteringsprotocol voor fecale *E. coli* op vleeskalverenbedrijven (bootstrap analyse, 1000 iteraties), gebaseerd op zowel rationele, objectieve en praktische motieven.

Onze onderzoeken tonen aan dat een reductie van 20 naar 5 % van de dieren, en een reductie van 12 naar 7 antimicrobiële middelen een zekere betrouwbaarheid waarborgt voor het opmeten van antibioticumresistentie op vleeskalverenbedrijven. Volgende antibiotica werden geselecteerd: ampicilline (betalactams), oxytetracycline (tetracyclines), trimethoprim-sulphonamiden (gepotentieerde sulfonamiden), enrofloxacine (quinolones), neomycine, gentamicine (aminoglycosiden), spectinomycine (aminocyclitolen). De informatie van deze producten wordt voldoende geacht voor een eerste screening, terwijl bijkomende onderzoeken kunnen ingezet worden voor het opmeten van opkomende resistentie (*emerging resistances*, bijvoorbeeld cephalosporines) wanneer multi-resistentie wordt aangetoond tijdens de eerste screening.

Samenvattend kan worden geconcludeerd dat dit onderzoek duidelijk heeft gemaakt dat de vleeskalverenhouderij gekenmerkt wordt door een hoog antibioticumgebruik en een veelvuldig voorkomen van antibioticumresistentie in verschillende orgaansystemen. Op de melkvee- en zoogkoeienbedrijven is de situatie gunstig. De vernieuwende analytische methoden bleken zeer bruikbaar, vermits werd aangetoond dat, los van een determinerede invloed van het bedrijfstype, er een sterk verband bestaat tussen de hoeveelheid toegediende antibiotica en het resistentieniveau.

Zowel uit diergezondheids- als volkgezondheidsoverwegingen verdient de vleeskalverensector meer aandacht in de monitoringsprogramma's en onderzoeken met betrekking tot antibioticumresistentie. Onze resultaten wijzen erop dat het rectaal bemonsteren van 5% van de aanwezige vleeskalveren ter isolatie van de enterische indicatorbacterie *E. coli*, en het hierop verrichten van disk diffusie gevoeligheidsbepalingen voor een selectie van 7 antimicrobiële middelen, een relevante en betrouwbare resistentieopmeting kunnen waarborgen. Op die manier kunnen de middelen van een monitoringsprogramma zo efficiënt mogelijk ingezet worden.

Formatted: French (France)

Résumé

L'antibiorésistance des bactéries peut entraîner des échecs de la thérapie, une durée de traitement plus longue et une morbidité et mortalité accrue des maladies infectieuses. La résistance peut également s'échanger entre l'animal et l'homme. C'est la raison pour laquelle il est d'une importance capitale, tant pour la médecine vétérinaire que pour la santé publique de maintenir le niveau d'antibiorésistance le plus bas possible. Afin de contrôler le risque pour la santé publique, des programmes de surveillance ont été lancés dans différents pays. D'une part, on y enregistre globalement l'usage humain et vétérinaire d'antibiotiques. D'autre part, on y détermine le niveau d'antibiorésistance pour les pathogènes humains, les bactéries zoonotiques et les bactéries sentinelles (commensales) des animaux de rente. Les données globales des programmes de surveillance, habituellement basées sur des chiffres de vente et des pourcentages de résistance non détaillés, n'offrent pas la possibilité d'être reliées aux exploitations agricoles individuelles où règne une pression de sélection (usage des antibiotiques). Ce projet a tenté d'examiner en profondeur l'épidémiologie de l'antibiorésistance dans les élevages bovins en Belgique afin de développer un protocole d'échantillonnage au niveau du troupeau et de permettre ainsi de classer les élevages de manière efficace et d'en assurer le suivi dans le temps. Dans ce contexte, quatre objectifs étaient au centre des préoccupations.

Le premier objectif visait à établir un aperçu détaillé de l'usage d'antibiotiques dans les élevages bovins flamands en réalisant une mesure tous les quatre mois dans 5 élevages de vaches laitières, 5 élevages de vaches viandeuses et 5 élevages de veaux viandeux. La méthodologie utilisée permettait de réaliser une description quantitative et qualitative des administrations d'antibiotiques au niveau de l'animal, en élaborant un système d'enregistrement développé par l'organisation mondiale de la santé (WHO, defined daily dose). Grâce à celui-ci, une comparaison au niveau international est possible. Les mesures permettent de conclure que, en moyenne (min-max) pour 1000 animaux présents, respectivement 6,26 (3,23-10,86) et 5,42 (3,02-10,15) bovins sont traités journellement dans les élevages laitiers et viandeux (TI_{UDD}). On a constaté en outre que ces administrations s'écartaient peu des dosages recommandés (TI_{ADD}). Dans les élevages de veaux viandeux, pour 1000 animaux présents, en moyenne 272,02 (120,30-626,90) veaux étaient traités journellement (TI_{UDD}), principalement par des traitements oraux de groupe afin de contrôler les troubles respiratoires. Ces traitements s'écartaient toutefois considérablement des dosages recommandés selon les notices des produits administrés étant donné que les quantités présentes n'étaient destinées qu'à 104,52 (45,08-369,66) veaux pour 1000 (TI_{ADD}). En d'autres termes, près de trois fois plus de veaux viandeux ont été traités par rapport à ce qui était prévu dans les traitements recommandés.

Un deuxième objectif visait à mesurer avec précision la situation en matière d'antibiorésistance dans les élevages bovins flamands dans la flore entérique commensale grâce à des bactéries sentinelles, pour les pathogènes facultatifs du système respiratoire et le germe zoonotique *Salmonella*. Les antibiotiques inclus au départ dans l'étude étaient la benzylpénicilline, l'ampicilline, l'amoxicilline + acide clavulanique, le ceftiofur, l'érythromycine, la lincomycine, la tétracycline, la néomycine, la gentamicine, la spectinomycine, la streptomycine, l'acide nalidixique, la fluméquine, l'enrofloxacine et la combinaison de sulfamides/triméthoprime.

Dans la première mesure de résistance, il est apparu que les bactéries sentinelles Gram-positives habituelles, *Enterococcus (E.) faecium* et *E. faecalis* étaient isolées à une fréquence trop faible (fourchette moyenne de prévalence dans l'élevage: 0,4-12%) pour

pouvoir assurer un suivi des tendances. C'est pourquoi *Streptococcus (S.) bovis* a été introduit comme bactérie sentinelle alternative pour le cheptel bovin. Malgré une importante prévalence initiale sur base de l'identification phénotypique (fourchette moyenne de prévalence dans l'élevage: 25,8-73,0%), il est apparu après contrôle en comparant le typage des germes avec des techniques moléculaires (tADN-PCR) que seulement 63,4% des isolats avaient été correctement identifiés. La qualité des tests de sensibilité utilisés (tests de diffusion en disque) semblait également insuffisante pour d'une part suivre les tendances de manière précise et d'autre part démontrer un lien avec l'usage d'antibiotiques. La sensibilité et la spécificité des tests de diffusion en disque ont été examinées en comparant les résultats des tests avec ceux de la méthode de dilution en gélose qui a été considérée comme la *norme de référence*. Ensuite, on a calculé respectivement la valeur prédictive résistante (positive; *resistant predictive value*, RPV) et sensible (négative; *susceptible predictive value*, SPV) des tests de diffusion en disque. Les germes Gram-positifs du système digestif ne sont pas pris en compte dans les analyses ultérieures de ce projet.

La qualité tant de l'identification bactérienne que des déterminations de sensibilité a été également contrôlée de manière analogue pour le pathogène facultatif *Pasteurellaceae* dans le système respiratoire (*Pasteurella multocida* et *Mannheimia haemolytica* lato sensu). Il en ressort une bonne qualité de l'identification phénotypique (> 93,5%) et la majorité des combinaisons bactérie-antibiotique examinées lors des tests de diffusion en disque montre une bonne fiabilité. Plus particulièrement, les 14 RPV calculées étaient toutes supérieures à 85 % et les 7 des 10 SPV calculables étaient supérieures à 90 %.

Dans aucun des 278 échantillons de fumier prélevés au début d'un cycle de production dans 5 élevages de veaux viandeux, *Salmonella* n'a pu être isolée.

Sur base des antibiorésistances mesurées tant dans les isolats de *E. coli* fécaux que dans les isolats de *Pasteurellaceae* respiratoires, une même tendance générale a pu être constatée: dans les élevages bovins laitiers et viandeux, la situation en matière de résistance est favorable (peu fréquente) et stable, tandis que dans les élevages de veaux viandeux la situation est défavorable (fréquence élevée de résistance et de multirésistance) et dynamique. L'aspect dynamique comporte une modification importante dans l'apparition des profils de résistance à la fin du cycle de production par rapport au début du cycle de production.

Le troisième objectif principal de ce projet était de vérifier les liens entre l'usage d'antibiotiques et la situation en matière de résistance. Grâce à la bonne précision de la surveillance des *E. coli* fécaux et *Pasteurellaceae* respiratoires, les analyses de ces deux écosystèmes ont été réalisées. Un modèle linéaire mixte a chaque fois été utilisé pour l'analyse, avec l'entreprise comme effet *random* et incidence de traitement, le type d'entreprise et l'âge (exprimé en mois) comme variables indépendantes. L'usage d'antibiotiques est rendu par les incidences de traitement, d'une part qui indiquent ce qui a été utilisé si la dose de traitement recommandée a été respectée (TI_{ADD}), et d'autre part adaptées à ce qui a réellement été utilisé dans l'entreprise (TI_{UDD}).

La relation entre l'usage d'antibiotiques (TI_{ADD} total ou TI_{UDD}, somme de toutes les voies d'administration), et la résistance (IRA = N constatations de résistance sur nombre total de détermination de sensibilité) a été analysé dans 15 élevages pour les isolats de *E. coli* fécaux (N= 1509, nombre total d'isolats *E. coli*). Dans l'analyse multivariante, une relation significative a été démontrée entre d'une part la TI_{ADD} ($P<0.001$) et d'autre part la TI_{UDD} ($P<0.001$) et l'IRA de *E. coli.* Le type d'élevage était ici également significatif ($P<0.001$), tandis que l'âge n'a montré aucune incidence.

La relation entre incidences de traitement (TI_{ADD} totale et $TI_{UDD)}$ et l'indice de résistance antimicrobienne (IRA) a été examinée pour 309 *Pasteurellaceae* de 5 élevages bovins viandeux (2 échantillons, N=102) et 5 élevages de veaux viandeux (T1 and T2,

N=207). Quel que soit le type d'élevage, un lien significatif a été trouvé entre la TI_{UDD} (P=0.002) et l'IRA de *Pasteurellaceae.* Pour l'analyse avec la TI_{ADD} un effet limite non significatif a été trouvé (P=0.093). Dans ces analyses également, le type d'entreprise a toujours un effet significatif sur l'IRA tandis que dans ce cas non plus l'âge ne présente aucune incidence.

Le quatrième objectif principal était de proposer une stratégie d'échantillonnage devant permettre, grâce à une méthodologie précise et efficace (bon marché) de classer les élevages en cheptels défavorables et favorables en ce qui concerne la situation en matière d'antibiorésistance. Dans ce but, ont été réalisées des simulations de réductions du protocole d'échantillonnage réellement utilisé pour les *E. coli* fécaux dans les élevages de veaux viandeux (analyse bootstrap, 1000 itérations), sur base de motifs rationnels, objectifs et pratiques.

Nos recherches montrent qu'une réduction de 20 à 5 % des animaux et une réduction de 12 à 7 des produits antimicrobiens garantissent une certaine fiabilité dans la mesure de l'antibiorésistance dans les élevages de veaux viandeux. Les antibiotiques suivants ont été sélectionnés: ampicilline (bêtalactames), oxytétracycline (tétracyclines), triméthoprime-sulfamides (sulfamides potentialisés), enrofloxacine (quinolones), néomycine, gentamicine (aminoglycosides), spectinomycine (aminocyclitoles). Les informations concernant ces produits sont considérées comme suffisantes pour une première étude, alors que des recherches complémentaires pourraient être entamées afin de mesurer la résistance émergente (*emerging resistances*, par exemple aux céphalosporines) lorsqu'une multirésistance est démontrée durant la première étude.

En résumé, on peut conclure que cette étude a clairement indiqué que l'élevage de veaux viandeux est caractérisé par un important usage d'antibiotiques et une apparition fréquente d'antibiorésistance dans différents systèmes organiques. Dans les élevages bovins laitiers et viandeux, la situation est favorable. Les méthodes analytiques innovantes semblent très utilisables car il a été démontré que, indépendamment d'une influence déterminante du type d'élevage, un lien important existe entre la quantité d'antibiotiques administrés et le niveau de résistance.

Pour des considérations tant de santé animale que de santé publique, le secteur des veaux viandeux mérite une plus grande attention dans les programmes de surveillance et les études relatives à l'antibiorésistance. Nos résultats soulignent que l'échantillonnage rectal de 5% des veaux viandeux présents pour isoler la bactérie sentinelle entérique *E. coli* et la réalisation sur ceux-ci de déterminations de sensibilité par diffusion en disque pour une sélection de 7 agents antimicrobiens peuvent garantir une mesure pertinente et fiable de la résistance. Les moyens d'un programme de surveillance peuvent, de cette manière, être engagés aussi efficacement que possible.

Summary

Antimicrobial resistance can result in therapy failure, a prolonged duration of treatment, and higher morbidity and mortality. Since resistance determinants can be exchanged between animals and humans, it is of the utmost importance for both veterinary and human medicine to keep the level of antimicrobial resistance as low as possible. To control the public health risks several countries have initiated antimicrobial resistance monitoring programmes. Such programmes record human and veterinary antimicrobial consumption. In addition, levels of antimicrobial resistance are monitored for human pathogens, zoonotic bacteria, and (commensal) indicator bacteria of production animals. Despite the ability to follow up regional trends based on national antimicrobial sales figures and crude resistance percentages, no feedback can be provided at the individual farm level where the actual antimicrobial selection pressures are exerted. The purpose of the project was to document the epidemiology of antimicrobial resistance in Belgian cattle herds in order to develop a sampling protocol that allows an efficient follow up in time and is able to categorise different farms. To this end, four central objectives were defined.

The first objective was to give a detailed overview of the antimicrobial use on 5 dairy, 5 beef, and 5 veal farms by applying the WHO supported treatment incidences based on the defined daily dose (DDD). The applied registration system not only permitted international quantitative comparisons (TI_{ADD}) but also a qualitative assessment. From the measurements it can be concluded that per 1000 animals present (at risk) an average of 5.39 (2.85-10.94) and 4.89 (2.25-7.16) daily were treated on the dairy and beef cattle herds, respectively (TI_{UDD}). In these farm types, no major deviations were noted between what should have been given according to the product leaflets (TI_{ADD}) and what was actually administered to the animals (TI_{UDD}). On the veal farms, an average of 272.02 (120.30-626.90) per 1000 animals at risk were daily treated, predominantly by oral group treatments for respiratory disorders. These treatments largely deviated from what actually should have been given to the animals if the product leaflets had been followed, since the amounts present at the farm were recommended (regulatory) for only 104.52 (45.08-369.66) calves per 1000 at risk. In other words, the antimicrobials present at the veal farms were administered to about three times the number of animals they were intended for.

A second objective was to measure accurately the resistance situation in Belgian cattle farms in the commensal enteric microbiota by means of indicator bacteria, in opportunistic pathogenic respiratory pathogens, and in the zoonotic organism *Salmonella*. The antimicrobial compounds included initially were benzylpenicillin, ampicillin, amoxicillin-clavulanic acid, ceftiofur, erythromycin, lincomycin, tetracycline, neomycine, gentamicin, spectinomycin, streptomycin, naladixic acid, flumequine, enrofloxacin and the combination sulphonamides/trimethoprim. From the presumptive resistance percentages it was deduced that the commonly monitored Gram-positive indicator bacteria *Enterococcus (E.) faecium* and *E. faecalis* were insufficiently present (range of average herd prevalence: 0.4-12.0%) to allow the following up of tendencies. Hence, *Streptococcus (S.) bovis* was introduced as an alternative indicator bacterium for cattle. Despite the high initial prevalence based on the phenotypical identification (range of average herd prevalence: 25.8-73.0%), through comparisons with molecular fingerprinting patterns (tDNA-PCR) only 63.4% of these isolates were correctly assigned as *S. bovis*. Moreover, the accurateness of the applied susceptibility tests (Kirby Bauer disk diffusion) were judged to be insufficient to reliably follow up trends and to explore relationships with antimicrobial consumption. This was investigated using sensitivity and specificity analysis, by comparing the disk diffusion test results with the agar

dilution method (golden standard), including a calculation of the *(positive) predictive value* (RPV) *for resistance* and the *(negative) predictive value* (SPV) *for susceptibility*. As a result of the inferior quality of data, the Gram-positive bacteria were not included in further analyses.

The quality of both the bacterial identification and the disk diffusion test results were assessed in the same way for the facultatively opportunistic pathogens belonging to the family *Pasteurellaceae* retrieved from the bovine respiratory tract (*Pasteurella multocida* and *Mannheimia haemolytica* sensu lato). The quality of the presumptive phenotypical identification received a good score (> 93.5%) and the greater part of the investigated bacterium-antimicrobial combinations of the disk diffusion susceptibility testing proved to be reliable as well. All 14 calculated RPVs exceeded 85 % and 7 of the 10 calculable SPVs were in excess of 90 %.

From none of the 278 faecal samples taken at the beginning of the production cycle in 5 veal calves farms, *Salmonella* was isolated.

The monitored resistance prevalence in both faecal *E. coli* and respiratory *Pasteurellaceae* demonstrated a similar pattern: whereas the resistance situation on dairy and beef herds is favourable (relatively low) and stable in time, the resistance situation in the veal calves farms is unfavourable (high frequency including multi-resistance) and in addition dynamic. The latter aspect was reflected by a change in resistance patterns towards the end of the 6 month production cycle.

Objective number three was to find associations between antimicrobial use and resistance. Because of the reliable accuracy of the faecal *E. coli* and respiratory *Pasteurellaceae*, analyses were performed on these two ecosystems. A linear mixed model was used, with herd as random effect and treatment incidence, herd type and age (expressed in months) as independent variables. The antimicrobial consumption was firstly expressed as treatment incidences, in case leaflet recommendations would have been respected (TI_{ADD}), and secondly adjusted to the actually administered regimens (TI_{UDD}).

The association between antimicrobial consumption (total TI_{ADD} of TI_{UDD}, sum of all administrations), and the resistance (expressed as antimicrobial resistance index (ARI) = N resistance observations divided by the total number of susceptibility tests per isolate) was analysed on 15 herds for *E. coli* isolates (N= 1509). In the multivariate analysis both the TI_{ADD} (P<0.001) as well as the TI_{UDD} (P<0.001) significantly affected the ARI of *E. coli.* The herd type was also significant (P<0.001), whereas age did not demonstrate any effect.

The relationship between the total TI_{ADD}/TI_{UDD} and the ARI was investigated for 309 *Pasteurellaceae* originating from 5 beef (2 sample moments, N=102) and 5 veal calves farms (T1 and T2, N=207). An independent association was found between the TI_{UDD} (P=0.002) and the ARI of the *Pasteurellaceae.* The analysis with the TI_{ADD} demonstrate a borderline non-significant effect (P=0.093). Similarly, in these analyses the herd type demonstrated a significant effect on the ARI, whereas no effect of age was observed.

The fourth central objective was to propose a sampling strategy that would allow classifying herds accurately and efficiently into categories with a favourable and an unfavourable resistance situation. For this purpose, simulations were performed while reducing the actual applied sampling strategy for faecal *E. coli* on veal calves farms (bootstrap analysis, 1000 iterations), based on rational, objective and logistic motivations.

The study demonstrated that a reduction from 20 to 5 % of the animals, and a reduction of 12 to 7 antimicrobial compounds guaranteed a reliable measurement of the antimicrobial resistance situation on the veal farms. The following antimicrobial agents were selected: ampicillin (beta-lactams), oxytetracycline (tetracyclines), trimethoprim-

sulphonamides, enrofloxacin (quinolones), neomycine, gentamicin (aminoglycosides), spectinomycin (aminocyclitols). The information obtained by testing these compounds was considered to be sufficient for a first screening, whereas additional testing might be needed to screen for emerging resistances (e.g. cephalosporins) in case multi-resistance results from preliminary testing.

In summary the veal herds were characterised by a high antimicrobial consumption and an abundant occurrence of antibiotic resistance in multiple organ systems. The situation is much more favourable on dairy and beef farms. The newly applied analytic methods proved to be very helpful since a strong relation was found between antimicrobial treatment incidences and the occurrence of resistance, irrespective of the (significant) effect of herd type.

Both from an animal health and public health point of view, the veal calves require more attention in monitoring programmes focusing on antimicrobial resistance. Our results suggest that a reliable resistance prevalence determination can be obtained by sampling 5% of the veal calves present to isolate the faecal indicator bacterium *E. coli*, and using a selection of 7 antimicrobial compounds. This will be helpful in the performance of antimicrobial resistance monitoring programmes.

Referenties

Aarestrup, F.M., Jensen, N.E. 1998. Development of penicillin resistance among *Staphylococcus aureus* isolated from bovine mastitis in Denmark and in other countries. *Microbial Drug Resistance* 3, 247-256.

Anoniem. 1994. Annuaire des taureaux blanc-bleu-belge, C.I.A. Linalux A.S.B.L.

Anoniem. 1997. Eindverslag van de reflectiegroep: productie en vraag, productietechnieken, fokbeleid. Ministry of Agriculture and Middle Classes, World Trade Center III, Boulevard Simon 30, 1000 Brussels.

Anoniem. 2001. Compendium van de farmaceutische specialiteiten voor diergeneeskundig gebruik 2001-2002, 10de uitgave, p.1-1312. Algemene Vereniging van de Geneesmiddelenindustrie, Brussel, België.

Anoniem. 2003. Gecommentarieerd Geneesmiddelen Repertorium voor Diergeneeskundig Gebruik 2003, p. 1-222. Belgisch Centrum voor Farmacotherapeutische Informatie, Brussel, België.

Avorn J.L., Barrett J.F., Davey P.G., McEwen S.A., O'Brien T.F., Levy S.B. 2001. Antibiotic resistance: synthesis of recommendations by expert policy groups. Alliance for the Prudent Use of Antibiotics. World Health Organization (WHO/CDS/CSR/DRS/2001.10)

Baele M., Baele P., Vaneechoutte M., Storms V., Butaye P., Devriese L.A., Verschraegen G., Gillis M., Haesebrouck F. 2000. Application of tDNA-PCR for the identification of enterococci. *Journal of Clinical Microbiology* 38, 4201-4207.

Baele M, Storms V, Haesebrouck F, Devriese LA, Gillis M, Verschraegen G, de Baere T, Vaneechoutte M. 2001. Application and evaluation of the interlaboratory reproducibility of tRNA intergenic length polymorphism analysis (tDNA-PCR) for identification of *Streptococcus* species. *Journal of Clinical Microbiology* 39, 1436-1442.

Bager F. 1999. DANMAP 98 - Consumption of antimicrobial agents and occurrence of antimicrobial resistance in bacteria from food animals, food and humans in Denmark, Danish Zoonosis Centre, 1999.

Barbosa, T.M., Levy, S.B. 2000. The impact of antibiotic use on resistance development and persistence. *Drug Resistance Update* 3, 303-311.

Bennedsgaard, T.W. 2003. Reduced use of veterinary drugs in organic dairy herds. PhD Thesis, The Royal Veterinary and Agricultural University, Frederiksberg, Denmark, pp11-12.

Bywater R.J. 2000 Sense and Nonsense in Surveillance Programs. *Acta Veterinaria Scandinavica*: S93: 119-127.

Cain D., Malouin F., Dargis M., Harel J., Gottschalk M. 1995. Alterations in penicillin binding proteins in strains of *Streptococcus suis* possessing moderate and high levels of resistance to penicllin. *FEMS Microbiology Letters* 130, 121-127.

Catry B., Laevens H., Devriese L.A., Opsomer G., de Kruif A. 2003. Antimicrobial resistance in livestock. Journal of Veterinary Pharmacology and Therapeutics 26, 81-93.

Catry B., Baele M., Opsomer G., de Kruif A., Decostere A., Haesebrouck F. 2004. tRNA-Intergenic Spacer PCR for the Identification of *Pasteurella* and *Mannheimia* spp. *Veterinary Microbiology* 98, 251-260.

Catry B., Haesebrouck F., De Vliegher S., Feyen B., Vanrobaeys M., Opsomer G., Schwarz S., de Kruif A. 2005. Variability in acquired resistance of *Pasteurella* and *Mannheimia* isolates from the nasopharynx of calves with particular reference to different herd types. Microbial Drug Resistance **11**, 387-394.

Caprioli A., Busani L., Martel J.L., Helmuth, R. 2000. Monitoring of antibiotic resistance in bacteria of animal origin: epidemiological and microbiological methodologies. *International Journal of Antimicrobial Agents* 14, 295-301.

Chauvin, C., Madec, F, Guillemot, D., Sanders, P. 2001. The crucial question of standardisation when measuring drug consumption. *Veterinary Research* 32, 533-543.

Chauvin, C., Madec, F. 2004. Quantitative analysis of antibiotic consumption in turkey broiler production. In: Reid S.W.J., Menzies F.D., Russell A.M. (Eds.), Proc. Society for Veterinary Epidemiology and Preventive Medicine Conference, Martigny, 2004, pp. 127-133.

Citron, D.M., Warren, Y.A., Fernandez, H.T., Goldstein, M.A., Tyrrell, K.L., and E.J. Goldstein. 2005. Broth microdilution and disk diffusion tests for susceptibility testing of *Pasteurella* species isolated from human clinical specimens. *Journal of Clinical Microbiology*. 43, 2485-2488.

Coopman, F., Peelman, L., Van Zeveren, A. 2000. De meest voorkomende erfelijke afwijkingen bij het Belgische witblauw vleesvee. *Vlaams Diergeneeskundig Tijdschrift* 69, 323-333.

Coopman, F., Gengler, N., Groen, A.F., De Smet, S., Van Zeveren, A. 2004. Comparison of external morphological traits of newborns to inner morphological traits of the dam in the double-muscled Belgian Blue Beef breed. *Journal of Animal Breeding and Genetics* 121, 128-134.

Devriese L.A., Laurier L., De Herdt P., Haesebrouck F. 1992. Enterococcal and streptococcal species isolated from faeces of calves, young cattle and dairy cows. *Journal of Applied Bacteriology* 92, 29-31.

Devriese, L.A., Haesebrouck, F., Hommez, J., Vandermeersch, R. 1997. A 25-year survey of antibiotic susceptibility testing in *Staphylococcus aureus* from bovine mastitis in Belgium, with special reference to penicillinase. *Vlaams Diergeneeskundig Tijdschrift* 66, 170-173.

Dewulf J., Catry B., Timmerman T., Opsomer G., de Kruif A., Maes D. 2006. Tetracycline-resistance in lactose-positive enteric coliforms originating from Belgian fattening pigs: degree of resistance, multiple resistance and risk factors. *Preventive Veterinary Medicine* 78, 339-351.

Grave, K., Greko, C., Nilsson, L., Odensvik, K., Mørk, T., Rønning, M., 1999. The usage of veterinary antibacterial drugs for mastitis in cattle in Norway and Sweden during 1990-1997. *Preventive Veterinary Medecine* 42, 45-55.

Heinrichs, A.J., Hargrove G.L. 1987. Standards of weight and height for Holstein heifers. *Journal of Dairy Science* 70, 653-666.

Helmuth R., Protz D. 1997. How to Modify Conditions Limiting Resistance in Bacteria in Animals and Other Reservoirs. Clinics of Infectious Diseases S24, 136-138.

Hubert, S.K., Nguyen, P.D., and R.D. Walker. 1998. Evaluation of a computerized antimicrobial susceptibility system with bacteria isolated from animals. *Journal of Veterinary Diagnostics and.Investigations* 10: 164-168.

Huovinen P. 1999. Bacterial Resistance; an Emerging Health Problem. *Acta Veterinaria Scandinavica* S92, 7-13.

Jorgensen, J.H., M.J. Ferraro. 1998. Antimicrobial susceptibility testing: general principles and contemporary practices. *Clinical Infectious Diseases* **26**: 973-980.

Kaneene, J.B., Miller R. 1992. Description and evaluation of the influence of veterinary presence on the use of antibiotics and sulfonamides in dairy herds. *Journal of American Veterinary Medicines Association* 201, 68-76.

Martel A., Baele M., Devriese L.A., Goossens H., Wisselink H.J., Decostere A., Haesebrouck F. 2001. Prevalence and mechanism of resistance against macrolides and lincosamides in *Streptococcus suis* isolates. *Veterinary Microbiology* **83:**, 287-297.

McGowan, J.E. 2000. The Impact of Changing Pathogens of Serious Infections in Hospitalized Patients. *Clinical Infectious Diseases* S31, 124-130.

Mevius D.J., Veldman, K.T., van der Giessen A., van Leeuwen, W.J. 2000. Eerste resultaten van de monitoring van resistentie in Nederland. *Tijdschrift voor Diergeneeskunde* 125, 143-146.

NCCLS-National Committee for Clinical Laboratory Standards. 2002. Performance standards for antimicrobial disk and dilution susceptibility tests for bacteria isolated from animals; approved standard-second edition, M31-A2, National Committee for Clinical Laboratory Standards, Wayne, Pennsylvania, USA.

NCCLS-National Committee for Clinical Laboratory Standards. 2004. Performance standards for antimicrobial disk and dilution susceptibility tests for bacteria isolated from animals; informational supplement, M31-S1, National Committee for Clinical Laboratory Standards, Wayne, Pennsylvania, USA.

Nielsen, J.P., Petersen, H.H., Bak, H. 2002. Antimicrobial consumption in Danish finishing herds. In: Proceedings of the 17th International Pig Veterinary Society Congress, Ames, Iowa, pp 184.

Nollet N., Houf K., Dewulf J., Catry B., De Zutter L., de Kruif A., Maes D. 2005. Variability in antimicrobial resistance among *Salmonella enterica* strains from fattening pigs and sows. Microbial Drug Resistance **12**, 74-81.

Rodriguez-Avial I, Rodriguez-Avial C, Culebras E, Picazo JJ. 2007. Fluoroquinolone resistance among invasive viridans group streptococci and Streptococcus bovis isolated in Spain. *International Journal of Antimicrobial Agents* 29:478-480.

Rogan, W.J., B. Gladen. 1978. Estimating prevalence from the results of screening test. Am. J. Epidemiol. **107**: 71-76.

Schwarz S., Kehrenberg C., Walsh T.R. 2001. Use of antimicrobial agents in veterinary medecine and food animal production. *International Journal of Antimicrobial Agents* 17, 431-437.

Teng L.J, Hsueh P.R, Ho S.W, Luh K.T. 2001. High prevalence of inducible erythromycin resistance among *Streptococcus bovis* isolates in Taiwan. *Antimicrobial Agents and Chemotherapy* 45, 3362-3365.

Timmerman T., Dewulf J., Catry B., Feyen B., Opsomer G., de Kruif A., Maes D. 2006. Quantification and evaluation of antimicrobial-drug use in group treatments for fattening pigs in Belgium. *Preventive Veterinary Medicine* **74:** 251-263.

Ungemach, F.R. 2000. Figures on quantities of antibacterials used for different purposes in the EU countries and interpretation. *Acta Veterinaria Scandinavica* S93, 89-98.

Van den Bogaard A.E.J.M., Stobberingh E.E. 2000. Epidemiology of resistance to antibiotics – Links between animals and humans. *International Journal of Antimicrobial Agents* 14, 327-335.

WHO 2002a. World Health Organization - Collaborating Centre for Drug Statistics Methodology, Guidelines for ATCvet Classification and DDD assignment, Oslo, Norway, [online] (2002a) http://www.whocc.no/atcvet [Consulted 3 August 2004].

WHO 2002b. World Health Organization - Collaborating Centre for Drug Statistics Methodology, Guidelines for ATC Classification and DDD assignment, Oslo, Norway, [online] (2002b) http://www.whocc.no/filearchive/word/guidelines_dddanimal.doc [consulted 31 May 2004]

Witte W. 2000. Ecological impact of antibiotic use in animals on different complex microflora: environment. *International Journal of Antimicrobial Agents* 14, 321-325.

Wray C., Gnanou J.C. 2000. Antibiotic resistance monitoring in bacteria of animal origin: analysis of national monitoring programmes. *International Journal of Antimicrobial Agents* 14, 291-294.

Lijst met Publicaties i.v.m. Antibioticumgebruik en –resistentie bij Rundvee

Artikels in internationale tijdschriften:

Catry B., Laevens H., Devriese L.A., Opsomer G., de Kruif A. 2002. Ontwikkeling en epidemiologie van antibioticumresistentie. *Vlaams Diergeneeskundig Tijdschrift* **71:** 53-62.

Catry B., Laevens H., Devriese L.A., Opsomer G., de Kruif A. 2003. Antimicrobial resistance in livestock. *Journal of Veterinary Pharmacology and Therapeutics* **26:** 81-93.

Catry B., Govaere J.L.J., Devriese L., Laevens H., Haesebrouck F., de Kruif A. 2002. Boviene enzoötische bronchopneumonie: prevalentie van pathogenen en hun antibioticumgevoeligheid. *Vlaams Diergeneeskundig Tijdschrift* **71:** 348-354.

Catry B., Baele M., Opsomer G., de Kruif A., Decostere A., Haesebrouck F. 2004. tRNA-intergenic spacer PCR for the identification of *Pasteurella* and *Mannheimia* spp. *Veterinary Microbiology* **98:** 251-260.

Catry B., Haesebrouck F., De Vliegher S., Feyen B., Vanrobaeys M., Opsomer G., Schwarz S., de Kruif A. 2005. Variability in acquired resistance of *Pasteurella* and *Mannheimia* isolates from the nasopharynx of calves with particular reference to different herd types. *Microbial Drug Resistance* **11:** 387-394.

Catry B., Chiers K., Schwarz S., Kehrenberg C., Decostere A., de Kruif A. 2005. Fatal peritonitis caused by *Pasteurella multocida* capsular type F in calves. *Journal of Clinical Microbiology* **43:** 1480-1483.

Kehrenberg C., Catry B., Haesebrouck F., de Kruif A., Schwarz S. 2005. A novel spectinomycin/streptomycin resistance gene, *aadA14*, from *Pasteurella multocida*. *Antimicrobial Agents and Chemotherapy* **49:** 3046-3049.

Kehrenberg C., Catry B., Haesebrouck F., de Kruif A., Schwarz S. 2005. *tet*(L)-mediated tetracycline resistance in bovine *Mannheimia* and *Pasteurella* isolates. *Journal of Antimicrobial Chemotherapy* **56:** 403-406.

Catry B., Govaere J.L.J., Vanholder T., Opsomer G., Decostere A., Haesebrouck F., de Kruif A. 2005. Nieuwe inzichten in Boviene enzoötische bronchopneumonie. *Vlaams Diergeneeskundig Tijdschrift* **74:** 412-419.

Casteleyn C., Dewulf J., Catry B., de Kruif A., Maes D. 2006. Antibioticumresistentie in *Escherichia coli* bij landbouwhuisdieren, hazen, septisch materiaal en oppervlaktewater in Vlaanderen. *Vlaams Diergeneeskundig Tijdschrift* **75:** 23-30.

Catry B., Decostere A., Schwarz S., Kehrenberg C., de Kruif A., Haesebrouck F. 2006. Detection of tetracycline resistant and susceptible *Pasteurellaceae* in the nasopharynx of loose group housed calves. *Veterinary Research Communications* **30:** 707-715.

Catry B. Use of nasal swabs in diagnosis of respiratory disease. 2006. *The Veterinary Record* **158**: 455-456.

Catry B., Boyen F., Baele M., Dewulf J., de Kruif A., Vaneechoutte M., Haesebrouck F., Decostere A. Recovery of *Moraxella ovis* from the bovine respiratory tract and differentiation of *Moraxella* species by tDNA-intergenic spacer PCR. 2007. *Veterinary Microbiology* **120:** 375-380.

Catry B., Dewulf J., Goffin T., Decostere A., Haesebrouck F., de Kruif A. 2007. Antimicrobial resistance patterns of *E. coli* through the digestive tract of veal calves. *Microbial Drug Resistance* **13:** 147-150.

Catry B., Dewulf J., de Kruif A., Vanrobaeys M., Haesebrouck F., Decostere A. 2007. Accuracy of susceptibility testing of *Pasteurella multocida* and *Mannheimia haemolytica*. *Microbial Drug Resistance* **13:** in druk.

Proceedings in Internationale congressen:

Catry B., Devriese L.A., Laevens H., De Vliegher S., Vaneechoutte M., Opsomer G., de Kruif A. 2001. Antibiotic susceptibility and resistance of *Staphylococcus chromogenes* from bovine mastitis. Proceedings of the 11th International Conference on Production Diseases in Farm Animals, Copenhagen, Denmark. Acta Veterinaria Scandinavica, **98** (Suppl.), pp. 266-267.

Catry B., Govaere J.L.J., Laevens H., Devriese L.A., de Kruif A. 2002. Low prevalence of antimicrobial resistance in bovine respiratory pathogens in Flanders, Belgium. Proceedings of the International Congress on Antimicrobial Agents in Veterinary Medicine, 4-8 augustus, Helsinki, Finland, p. 88.

Feyen B., Catry B., Opsomer G., Vanrobaeys M., de Kruif A. 2003. *Streptococcus bovis* as indicator bacterium for cattle. Proceedings of the 11th Annual Conference of the Flemish Society for Veterinary Epidemiology and Economics, December 11, 2003, Torhout, Belgium, pp. 55-58.

Catry B., Feyen B., Raeymaekers T., Opsomer G., Timmerman T., Schwarz S., Haesebrouck F., de Kruif A. (2003). Antimicrobial resistance in bovine commensal nasal *Pasteurellaceae,* Proceedings of the 11th Annual Conference of the Flemish Society for Veterinary Epidemiology and Economics, Torhout, Belgium, December 11, pp. 59-61.

Catry B., Verloo D., Feyen B., de Kruif A. 2003. Distributional considerations in sampling indicator bacteria for antimicrobial resistance. Proceedings of the 11th Annual Conference of the Flemish Society for Veterinary Epidemiology and Economics, Torhout, Belgium, December 11, pp. 63-65.

Feyen B., Catry B., Timmerman T. 2004. Targeted studies on antimicrobial use and antimicrobial resistance. Pre-AAVM Discussion on Monitoring and Quantifying Antimicrobial Use in Agriculture and Veterinary Medicine. Ottawa, Canada, June 13.

Catry B., Feyen B., Timerman T., Baele M., de Kruif A., Haesebrouck F. 2004. Susceptibility profiles of bovine *Pasteurellaceae* under different housing conditions. Proceedings of the 2nd International Conference on Antimicrobial Agents in Veterinary Medicine (AAVM), Ottawa, Canada, June 13-17. p. 42.

Feyen B., Catry B., Laevens H., Opsomer G., Vanrobaeys M., de Kruif A. 2004. Antibiotic usage on cattle farms in Flanders. Proceedings of the 2nd International Conference on Antimicrobial Agents in Veterinary Medicine (AAVM), Ottawa, Canada, June 13-17. p. 77.

Catry B., Feyen B., Opsomer G., Haesebrouck F., de Kruif A. 2004. Antimicrobial resistance in commensal respiratory *Pasteurellaceae*. Proceedings 23rd World Buiatrics Congress, Quebec, Canada; Le Médecin Vétérinaire du Québec, Vol 34, Nos 1 et 2.

Feyen B., Catry B., Opsomer G., Vanrobaeys M., de Kruif A. 2004. *Streptococcus bovis* as indicator bacterium for antimicrobial resistance in cattle**.** Proceedings 23rd World Buiatrics Congress, Quebec, Canada; Le Médecin Vétérinaire du Québec, Vol 34, Nos 1 et 2.

Kehrenberg C., Catry B., Haesebrouck F., de Kruif A., Schwarz S. 2005. A novel spectinomycin/streptomycin resistance gene, *aadA14*, from *Pasteurella multocida*. Proceedings of the 45th Interscience Conference on Antimicrobial Agents and Chemotherapy, New Orleans, Louisiana, USA, September 21 – 24.

Catry B., Schwarz S., Deprez P., Kehrenberg C., Croubels S., Opsomer G., Haesebrouck F. 2005. Effect of enrofloxacin on the presence of *Pasteurella multocida* in the bovine nasopharynx. Proceedings of the ASM conference on *Pasteurellaceae*, Big Island, Hawaii, October 23-26, p. 74-75.

Catry B., Dewulf J., Feyen B., Vanrobaeys M., Opsomer G., de Kruif A., Decostere A, Schwarz S. 2005. Dynamics of antimicrobial susceptibilities of *Pasteurella multocida* and *Mannheimia haemolytica* sensu lato from veal calves. Proceedings of the ASM conference on *Pasteurellaceae*, Big Island, Hawaii, October 23-26, p. 76.

Schwarz S. Kehrenberg C., Catry B., Haesebrouck F., de Kruif A. 2005. Occurence of *tet*(L), a tetracycline resistance gene from Gram-positive bacteria, in bovine *Pasteurella* and *Mannheimia* isolates. Proceedings of the ASM conference on *Pasteurellaceae*, Big Island, Hawaii, October 23-26, p. 50-51.

Kehrenberg C., Catry B., Haesebrouck F., de Kruif A., Schwarz S. 2005. *aadA14*, a novel adenylyltransferase gene from *Pasteurella multocida* that mediates resistance to spectinomycin and streptomycin. Proceedings of the ASM conference on *Pasteurellaceae*, Big Island, Hawaii, October 23-26, p. 50.

Catry B., Dewulf J., Vanrobaeys M., Opsomer G., Haesebrouck F., Decostere A. 2006. Accuracy of bovine *Pasteurellaceae*. Proceedings of the Third international conference on Antimicrobial Agents in Veterinary Medicine (AAVM), Orlando, Florida, USA, May 16-20, p. 37.

Catry B., Croubels S., Schwarz S., Deprez P., Kehrenberg C., Opsomer G., De Backer P., Decostere A., Haesebrouck F. 2006. Effect of Enrofloxacin Administration on the Occurrence of *P. multocida* in the Bovine Nasopharynx. Proceedings of the Third international conference on Antimicrobial Agents in Veterinary Medicine (AAVM), Orlando, Florida, USA, May 16-20. p. 79.

Dewulf J., Casteleyn C., Catry B., de Kruif A., Maes D. 2006. Occurrence of Antimicrobial Resistance in *Echerichia Coli* in Faeces samples of dairy cattle, pigs, poultry, humans, hare, and surface water in flanders (Belgium). Proceedings of the Third international conference on Antimicrobial Agents in Veterinary Medicine (AAVM), Orlando, Florida, USA, May 16-20. p. 38.

Catry B., Haesebrouck F., Opsomer G., Decostere A., de Kruif A. 2006. An update on bovine pasteurellosis. Proceedings of the 24th World Buiatrics Congress, Nice, France, October 17-19.

Catry B., Dewulf J., Vanrobaeys M., de Kruif A., Haesebrouck F., Decostere A. 2006. Relationship between antimicrobial treatment incidence and antimicrobial resistance of *E. coli* in veal calves. Proceedings of the 14th Annual Conference of the Flemish Society for Veterinary Epidemiology and Economics, Merelbeke, Belgium, September 22.

Cools S., Catry B., Vanrobaeys M., de Kruif A. , Opsomer G. 2007. Antimicrobial resistance of staphylococci isolated out of subclinical and clinical cases of mastitis. Heifer Mastitis Conference, Faculty of Veterinary Medicine, Ghent University, Merelbeke, June 25-27.

Bijlage

Addendum 1. Interpretatiecriteria voor de gevoeligheidstesten van *Pasteurellaceae*

	Disk diffusie				Agar dilutie		
**Antibioticum**		**Inhibitie zone** (mm)			**Minimale inhibitorische concentratie** (µg/mL)		
	Lading	S[a]	I	R	Range	S	R
**Ampicilline**	33 µg	≥28	27-24	≤23	0.03-128	≤1	≥4
**Ceftiofur**	30 µg	≥21	20-18	≤17	0.03-128	≤2	≥8
**Oxytetracycline**	80 µg	≥23	22-20	≤19	0.06-128	≤4	≥16
**Gentamicine**	40 µg	≥23	22-20	≤19	0.06-128	≤4	≥16
**Florfenicol**	30 µg	≥19	18-15	≤14	0.03-128	≤2	≥8
**Enrofloxacine**	10 µg	≥23	22-17	≤16	0.03-64	≤0.25	≥2
**TMP/S[b]**	5.2+240 µg	≥28	27-24	≤23	0.06/1.14-128/2432	≤2/38	≥8/76

[a] S: gevoelig; I: intermediair resistent; R: resistent, [b] TMP/S: trimethoprim-sulfonamiden. Rechthoeken: referentiewaarden volgens CLSI 2004. Alle andere grenswaarden zijn afkomstig van de producent van de antibioticumtabletten (Rosco, Taarstrup, Denmark).

Addendum 2. Interpretatiecriteria voor de gevoeligheidstesten van enterokokken (ENT) en streptokokken (STREP).

	Disk diffusie				Agar dilutie		
**Antibioticum**		**Inhibitie zone** (mm)			**Minimale inhibitorische concentratie** (µg/mL)		
	Lading	S[a]	I	R	Range	S	R
**Ampicilline *ENT***	(33 µg)	(≥20)	(-)	(-)	0.03-256	**≤8**	**≥16**
**Ampicilline *STREP***	(33 µg)	(≥30)	(29-21)	(≤20)	0.03-256	**≤0.25**	**≥8**
**Penicilline *ENT***	5 µg	**≥10**	-	-			(≥16)
**Penicilline *STREP***	5 µg	**≥26**	**25-13**	**≤12**		(≤0.12)	(≥4)
**Oxytetracycline *ENT***	80 µg	**≥23**	**22-20**	**≤19**	0.03-256	**≤4**	**≥16**
**Oxytetracycline *STREP***	80 µg	**≥26**	**25-23**	**≤22**	0.03-256	**≤2**	**≥8**
**Lincomycine**	19 µg	**≥26**	**25-23**	**≤22**	0.03-256	**≤2**	**≥8**
**Erythromycine *ENT***	78 µg	**≥26**	**25-19**	**≤18**	0.03-256	**≤0.5**	**≥8**
**Erythromycine *STREP***	78 µg	**≥28**	**27-24**	**≤23**	0.03-256	**≤0.25**	**≥1**

[a] S: gevoelig; I: intermediair resistent; R: resistent, [b] TMP/S: trimethoprim-sulfonamiden. Rechthoeken: referentiewaarden volgens CLSI 2004. Alle andere grenswaarden zijn afkomstig van de producent van de antibioticumtabletten (Rosco, Taarstrup, Denmark).

federale overheidsdienst
VOLKSGEZONDHEID, VEILIGHEID VAN DE VOEDSELKETEN EN LEEFMILIEU
